# Targeting autophagy with natural products as a potential therapeutic approach for diabetic microangiopathy

**DOI:** 10.3389/fphar.2024.1364616

**Published:** 2024-04-10

**Authors:** Fengzhao Liu, Lijuan Zhao, Tao Wu, Wenfei Yu, Jixin Li, Wenru Wang, Chengcheng Huang, Zhihao Diao, Yunsheng Xu

**Affiliations:** ^1^ First Clinical Medical College, Shandong University of Traditional Chinese Medicine, Jinan, China; ^2^ College of Traditional Chinese Medicine, Shandong University of Traditional Chinese Medicine, Jinan, China; ^3^ Xi yuan Hospital, China Academy of Chinese Medical Sciences, Beijing, China; ^4^ Department of Endocrinology, Shandong University of Traditional Chinese Medicine Affiliated Hospital, Jinan, China; ^5^ College of Acupuncture and Massage, Shandong University of Traditional Chinese Medicine, Jinan, China; ^6^ Department of Endocrinology, Second Affiliated Hospital of Shandong University of Traditional Chinese Medicine, Jinan, China

**Keywords:** autophagy, natural products, diabetic microangiopathy, diabetic kidney disease, diabetic retinopathy, diabetic cardiomyopathy, diabetic peripheral neuropathy

## Abstract

As the quality of life improves, the incidence of diabetes mellitus and its microvascular complications (DMC) continues to increase, posing a threat to people’s health and wellbeing. Given the limitations of existing treatment, there is an urgent need for novel approaches to prevent and treat DMC. Autophagy, a pivotal mechanism governing metabolic regulation in organisms, facilitates the removal of dysfunctional proteins and organelles, thereby sustaining cellular homeostasis and energy generation. Anomalous states in pancreatic β-cells, podocytes, Müller cells, cardiomyocytes, and Schwann cells in DMC are closely linked to autophagic dysregulation. Natural products have the property of being multi-targeted and can affect autophagy and hence DMC progression in terms of nutrient perception, oxidative stress, endoplasmic reticulum stress, inflammation, and apoptosis. This review consolidates recent advancements in understanding DMC pathogenesis via autophagy and proposes novel perspectives on treating DMC by either stimulating or inhibiting autophagy using natural products.

## 1 Introduction

Diabetes, a chronic metabolic disease, has seen an increasing global prevalence. Prolonged exposure of diabetic patients to high levels of glucose and fat in the environment triggers a sequence of lesions within the body’s microvasculature, termed diabetic microangiopathy (DMC). DMC is mainly categorized into diabetic kidney disease (DKD), diabetic retinopathy (DR), diabetic cardiomyopathy (DCM), and diabetic peripheral neuropathy (DPN) (Schiborn and Schulze, 2022), and primarily affects the kidney, retina, myocardium, nerve tissue, and toes. Diabetic foot complications often manifest in the advanced stages of DPN. Presently, the precise pathogenesis of DMC remains elusive in modern medicine and is likely associated with inflammatory responses, oxidative stress, vascular endothelial cell damage, and alterations in vascular permeability due to elevated glucose levels (Singh and Kulkarni, 2022; Zhu et al., 2023). Current clinical treatments for DMC primarily target these factors but exhibit limited efficacy (Nellaiappan et al., 2022). Hence, there is an urgent need to identify therapeutic targets and innovate new drugs for DMC.

Autophagy, akin to apoptosis and senescence, is a critical biological phenomenon. It degrades misfolded proteins and damaged organelles into smaller components, providing cells with necessary nutrients and materials for self-renewal (Maiuri et al., 2007). This process serves as a self-protective mechanism enabling cells to sense external stimuli or abnormal energy metabolism, crucial for maintaining cellular homeostasis (Dikic and Elazar, 2018; Bharath et al., 2021). Conversely, dysfunctional autophagy fails to maintain normal cellular protection and may, in turn, exert a dual effect, damaging cells and leading to various diseases such as heart disease, neurodegeneration, and metabolic disorders (Parzych and Klionsky, 2014). Hence, autophagy holds promise as a potential alternative for DMC therapy.

In recent decades, natural products (NPs) sourced from plants, animals, and microorganisms have attracted increased attention given their multifaceted components, ability to target multiple pathways, accessibility, and low toxicity levels (Newman and Cragg, 2016; Newman and Cragg, 2020). Recent studies have increasingly demonstrated the therapeutic potential of NPs in DR, DKD, DC, and DPN by modulating autophagy (Yao et al., 2018; Zhou P. et al., 2019; Yin et al., 2021; Liu P. et al., 2023; Liu T. et al., 2023). This article reviews recent research on autophagy and DMC, exploring the role and mechanism of NPs in enhancing DMC efficacy by regulating autophagy. Furthermore, NPs with autophagy-modulating abilities are highlighted as a promising therapeutic strategy for treating DMC.

## 2 Autophagy

The key to autophagy lies in the formation of autophagosomes and their subsequent fusion with lysosomes (Bharath et al., 2021). When the signal to regulate autophagy is received by the cell, various organelles within the cytoplasm generate an isolation membrane, also known as the phagophore. This membrane participates in the formation of phagosomes (Sanchez-Wandelmer et al., 2015; Morishita et al., 2017; Jung et al., 2020). The phagophore continues to elongate, encompassing the cell’s cytoplasm, damaged organelles, and long-lived proteins, eventually maturing into an autophagosome with a bimodal structure. Autophagosomes and lysosomes are transported by microtubules (MTs) to form autolysosomes (Korolchuk et al., 2011; Korolchuk and Rubinsztein, 2011). These autolysosomes degrade the inner membrane and contents, thereby providing for the cell’s needs and facilitating organelle renewal.

### 2.1 Classification of autophagy

**FIGURE 1:**
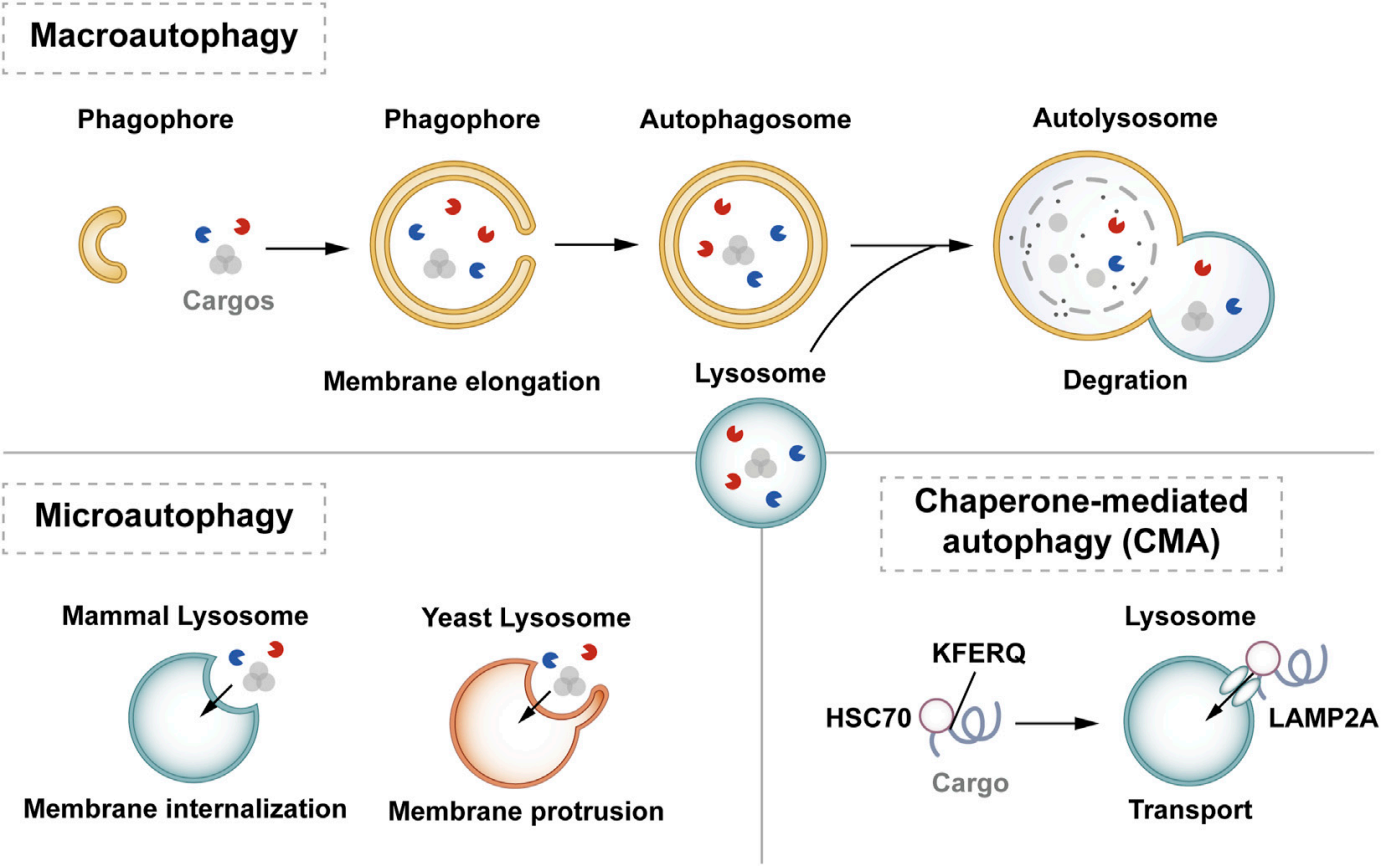
Classification of autophagy. Autophagy can be classified into three categories according to its cargos delivery pathway and membrane dynamics: macroautophagy, microautophagy, and CMA. Macroautophagy involves membrane elongation, microautophagy involves membrane invagination, and CMA does not entail membrane deformation. Hsc70 specifically recognises the KFERQ motif and then binds to the lysosomal membrane receptor LAMP2A to complete the autophagy process.

In mammalian cells, autophagy can be classified into macrophage-, microautophagy-, and chaperone-mediated autophagy (CMA) according to different modes of entry into the lysosome (Habshi et al., 2023). Macrophages are characterized by the presence of a large autophagosome measuring about 500 nm. The phagosomal membrane under macrophagy extends, forming autophagosomes with a two-layer vesicular structure that encapsulates cytoplasmic components and fuses with lysosomes. Conversely, microautophagy directly segregates and internalizes cytoplasmic components by inwardly invaginating the lysosomal membrane (Schuck, 2020). Two types of microautophagy have been identified: 1) lysosomal membrane invagination (or endosomal membrane in the nucleus microautophagy) and 2) lysosomal membrane protrusion (Wang et al., 2023). While the former predominates in mammals, *Komagataella phaffii* illustrates the typical lysosomal membrane protrusion in microautophagy. CMA involves the specific recognition of substrate protein molecules containing the KFERQ group by the molecular chaperone protein Hsc70 (also known as HSPA8). These complexes then bind to the lysosomal membrane receptor LAMP2A, ultimately resulting in substrate degradation within lysosomes (Hubert et al., 2022). Macroautophagy involves membrane elongation, and microautophagy involves membrane invagination. Unlike macroautophagy and microautophagy, CMA does not entail membrane deformation (Kaushik and Cuervo, 2018) (Figure 1).

According to substrate selectivity, autophagy can be divided into non-selective autophagy and selective autophagy (Jing and Lim, 2012; Vargas et al., 2023). Non-selective autophagy mainly refers to autophagy that is induced in most cases, such as autophagy induced by mammalian target of rapamycin (mTOR) (Glick et al., 2010). In selective autophagy, specific autophagy adapter proteins like SQSTM1/p62, interact with microtubule-associated protein light chain 3 (Lc3) located in the phagolysosome membrane, delivering substrates directly to autophagosomes for degradation (Lee et al., 2023). Identified types of selective autophagy are mitophagy, ribosomal autophagy, endoplasmic reticulum (ER) autophagy, and peroxisome autophagy (Adriaenssens et al., 2022). Both macroautophagy and microautophagy can exhibit selective or non-selective behavior. Macroautophagy is considered the main type of autophagy and is the most widely studied type. Therefore, hereafter in this review, we refer to macroautophagy as “autophagy”.

### 2.2 The process of autophagy

**FIGURE 2:**
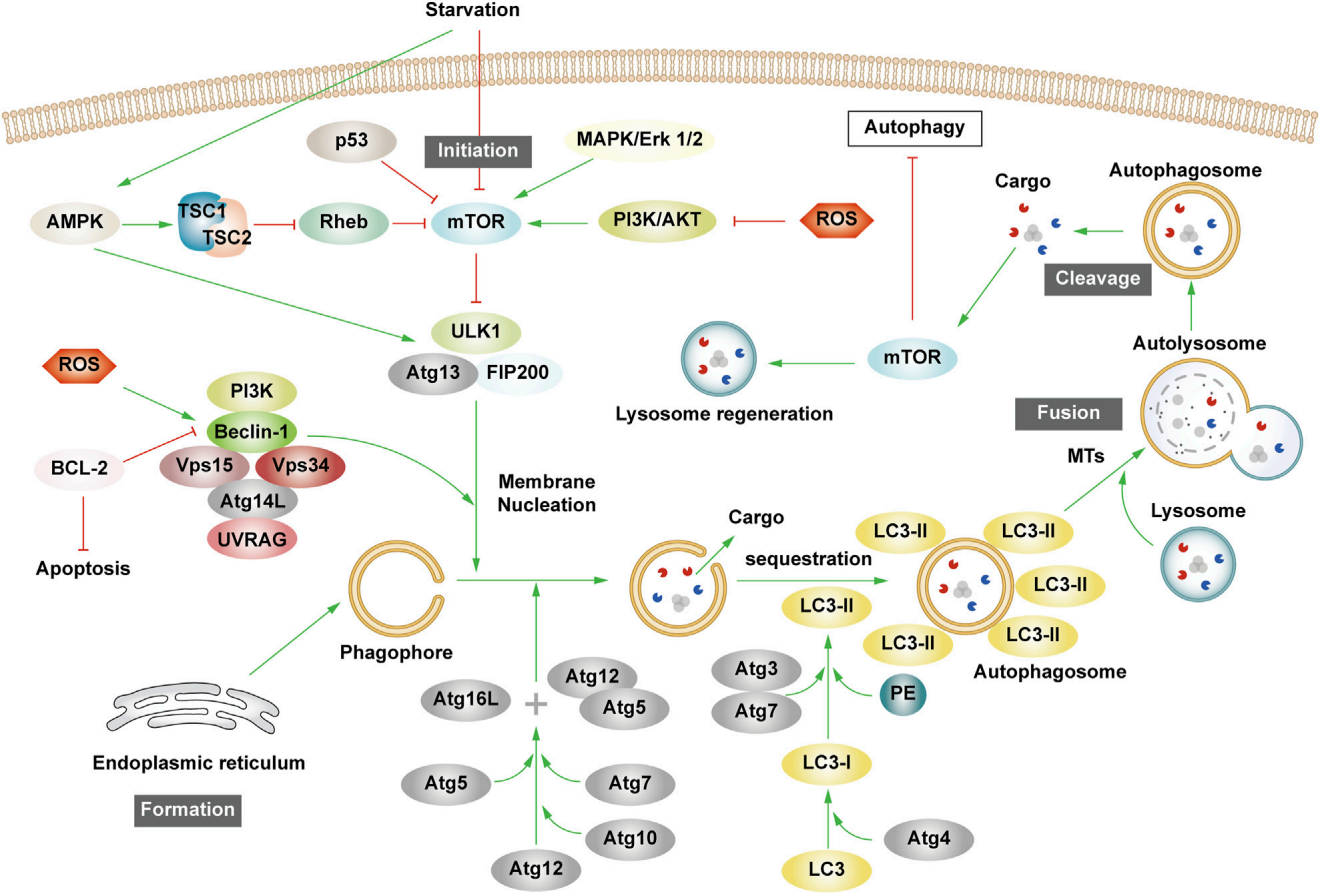
The process of autophagy. Green arrows indicate downstream cellular events; red lines indicate inhibition. Four major steps are involved in the process of autophagy: initiation, formation, fusion, and cleavage. Initiation: External stimuli such as starvation can affect the AMPK and mTOR pathways, leading to the activation of the ULK1 complex. Formation: The autophagosome formation is aided by two ubiquitin-like Atg-coupled systems and the PI3K complex. Fusion: Autophagosomes and lysosomes undergo transport via MTs to form autolysosomes. Cleavage: Degradation of cargos triggers autophagic lysosomal reorganization.

The essence of autophagy is actually intracellular membrane rearrangement, which occurs as a dynamic process called autophagic flux and occurs in the following four broad stages: initiation of autophagy → formation of phagosomes and autophagosomes → fusion of autophagosomes with lysosomes → cleavage of autophagosomes (Figure 2).

#### 2.2.1 Autophagy initiation

Autophagy is triggered in response to cell stress and can be considered as a coping mechanism to maintain homeostasis (Kume and Koya, 2015). The Ser/Thr signaling kinase mTOR regulates autophagosome formation. Pathways such as Akt and mitogen-activated protein kinase (MAPK) signaling pathways that activate mTOR inhibit autophagy, while those negatively regulating mTOR, such as the adenosine 5′-monophosphate (AMP)-activated protein kinase (AMPK) and p53-signaling pathways, promote autophagy (Alers et al., 2012). The ULK1 complex, comprising ULK1 or ULK2, FIP200, and mAtg13, forms a bridge *in vivo* between the upstream nutrient or energy receptors mTOR and AMPK and the downstream formation of autophagosomes (Leventhal et al., 2017). Under starvation conditions, AMPK is activated, mTOR is inactivated, and AMPK activation catalyzes ULK1 phosphorylation, thereby promoting autophagy. Conversely, in nutrient-rich conditions, AMPK is inactivated and mTOR binds to ULK1 serine 757 to inhibit the ULK1-AMPK interaction, leading to the inactivation of ULK1 and the eventual inhibition of autophagy (Alers et al., 2012). Interestingly, AMPK can inhibit Rheb, an mTORC1 activator, by activating Tuberous Sclerosis 1/2 (TSC1/2), which can then initiate autophagy (Inoki et al., 2006). Reactive oxygen species (ROS) can inhibit autophagy by suppressing TSC1/2 via the phosphatidylinositol-3-kinase (PI3K)/AKT pathway.

#### 2.2.2 Phagosome and autophagosome formation

Upon receiving autophagy regulation signals generated by cellular stress, phagosomes nucleate, expand, surround the cytoplasm, and finally form autophagosomes. Although the mechanism of phagosome formation is well understood, the constituent lipids and membrane modeling proteins involved in determining the shape and size of phagosomes have been at the center of controversy for decades (Tooze, 2013; Carlsson and Simonsen, 2015). It is now widely accepted that phagosomes originate near or on the ER, where several organelles including the mitochondria, Golgi complex, plasma membrane, and endosomes provide membranes for phagosome formation (Graef et al., 2013; Shibutani and Yoshimori, 2014; Sanchez-Wandelmer et al., 2015; Li J. et al., 2021). It has also been found that the ER exit site works synergy Atg9 in the autophagy-related gene (Atg) family to promote the assembly of autophagy mechanism, and may provide membranes for phagophore nucleation, maturation, and growth (Graef et al., 2013; Tooze, 2013; Sanchez-Wandelmer et al., 2015).

Phagosome nucleation hinges on local phosphatidylinositol 3-phosphate (PI3P) production. Regulated ULK complex recruitment to the phagocyte nucleation site phosphorylates beclin-1, activating the PI3K complex involving class III PI3K (PIK3C3), Beclin-1, VPS15, and Atg14L (Funderburk et al., 2010; Dooley et al., 2014).

Post-phagosome nucleation, membrane expansion involves two ubiquitin-like coupling systems: the Atg12 coupling system and Lc3-phosphatidylethanolamine (PE) coupling system (Pohl and Dikic, 2019). The Atg12 coupling system links Atg12 to Atg5 through Atg7 and Atg10 (E1-and E2-like enzymes), subsequently forming the Atg12-Atg5-Atg16 complex that contributes to LC3- and PE-coupling reactions. After the synthesis of LC3 protein, the C-terminal 5 peptide was cut-off by Atg4, resulting in cytoplasmic localization of LC3-I. LC3-I of LC3 was coupled with PE by ubiquitin-like reaction of Atg7 and Atg3 (E1-and E2-like enzymes, respectively) to form LC3-II. LC3-II stays on the inner and outer membrane of autophagosomes and serves as a marker of autophagy. Its upregulation is an indicator of autophagy activation and autophagosome formation (Glick et al., 2010). Post-autophagosome-lysosome fusion, LC3-II on the inner membrane is lysosomally degraded, and LC3-II on the outer membrane is cleaved by Atg4 to produce LC3-I for recycling (Mizushima and Komatsu, 2011).

#### 2.2.3 Autolysosome production and cleavage

MTs, as an internetwork of intracellular movement, are driven by specific kinesins, including the kinesin and cytoplasmic dynein (Kast and Dominguez, 2017). The interaction between autophagosomes dispersed in the cytoplasm and lysosomes enriched in the perikaryon depends on their two-way movement on microtubules. After maturation in the cytoplasm, autophagosomes are regulated by dynein to move to the perinuclear region. Under various stress conditions, intracellular pH rises, leading to the migration of lysosomes to the perinuclear region (Gross et al., 2007; Kimura et al., 2008). Autophagosomes and lysosomes in the same region fuse to form an autolysosome.

Upon autolysosome formation, lysosomal hydrolases degrade substrates, generating amino acids that reactivate mTOR via negative feedback, inhibit autophagy, regenerate the lysosome, and restore lysosomal levels—a process termed autophagic lysosome reorganization (Chen and Yu, 2018).

## 3 The molecular mechanism of autophagy and diabetic microvascular complications

### 3.1 Interaction between autophagy and cellular biological processes related to diabetic microvascular complications

**FIGURE 3:**
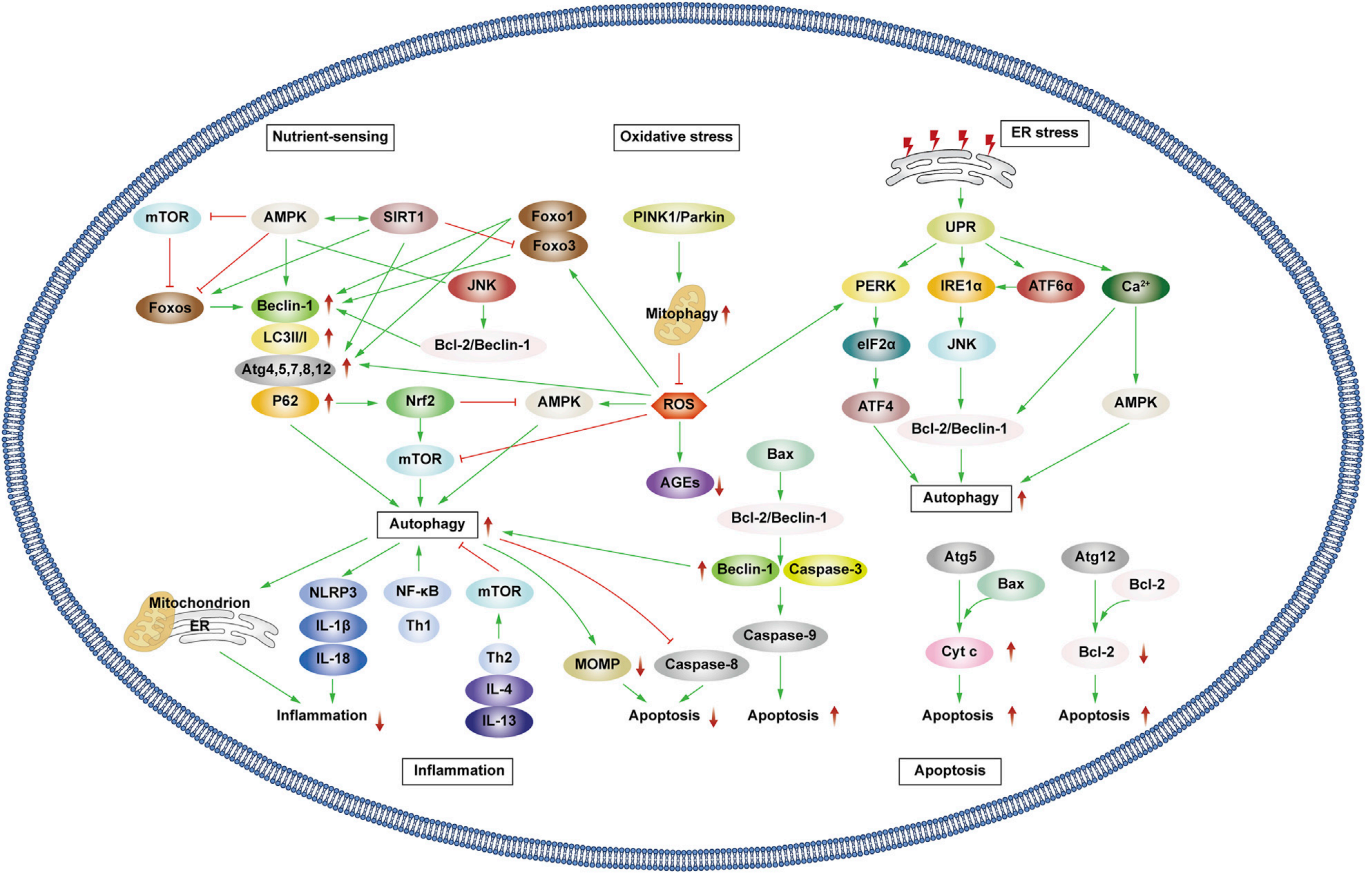
Autophagy and cellular biological processes. Green arrows indicate downstream cellular events; red lines indicate inhibition. Cellular biological processes mainly include nutrient sensing, oxidative stress, ER stress, inflammation and apoptosis. Cellular biological processes can regulate autophagy, which in turn can influence intracellular oxidative stress, inflammation, and apoptosis.

Patients experiencing DMC endure chronic hyperglycemia, fostering abnormalities in nutrient perception, ER stress, oxidative stress, inflammation, and apoptosis. This persistent state leads to the accumulation of damaged proteins and organelles over time, jeopardizing cellular physiological functions. Autophagy, a scavenging mechanism, plays a pivotal role in maintaining cellular homeostasis and combating DMC by influencing these biological processes. (Figure 3).

#### 3.1.1 Interaction between autophagy and nutrient sensing

Hunger is known to promote autophagy through nutrient-sensing pathways, including mTOR, AMPK, and Silent Information Regulator 1 (SIRT1) (Oza et al., 2021). Briefly, as outlined earlier, mTOR and AMPK exhibit inhibitory and promotional effects on autophagy. SIRT1, a histone deacetylase, senses cellular energy levels and has the potential to slow cellular aging, withstand external stress, and enhance metabolism. SIRT1 activates autophagy via deacetylation of FOXOs. Additionally, it forms molecular complexes by directly binding to Atg5, Atg7, and Atg8, restoring autophagy (Ganesan et al., 2017; Gautam et al., 2020; Zhou et al., 2021). Moreover, SIRT1 deacetylates p53, reducing p53 expression, hence activating autophagy. The regulation of p53 in autophagy depends on its cellular localization, contributing to its dual role (Lee, 2019; Cui et al., 2021). Additionally, SIRT1 activates AMPK by deacetylating liver kinase B1 (LKB1). In turn, AMPK promotes the activation of SIRT1. Under starvation conditions, SIRT1 expression is elevated, activating autophagy in response to nutrient deprivation.

#### 3.1.2 Interaction between autophagy and oxidative stress

Prolonged hyperglycemia results in oxidative stress within DMC-associated cells, leading to ROS accumulation and cellular oxidative damage. ROS accumulation triggers autophagy to reduce ROS levels, maintaining cellular homeostasis. ROS upregulate AMPK, FOXOs, MAPK, and c-JunN-terminal kinase (JNK) pathways or inhibit the PI3K/AKT/mTOR pathways, activating autophagy (Wong et al., 2010; Li et al., 2015; Hinchy et al., 2018; Kim et al., 2018). Notably, ROS influences Atg4 and ER stress pathways, promoting autophagy (Scherz-Shouval et al., 2007; Wible and Bratton, 2018). Primarily produced by damaged mitochondria, ROS levels are reduced by autophagy, particularly mitophagy, which selectively eliminates dysfunctional mitochondria, and help in maintaining cellular homeostasis. Additionally, p62 affects the Kelch-like ECH-associated protein 1 (Keap1)/Nuclear factor erythroid 2 related factor 2 (Nrf2) pathway, stimulating Nrf2 expression and enhancing mitophagy to counteract ROS (Wible and Bratton, 2018).

Mitophagy, chiefly regulated by the PTEN-induced kinase 1 (PINK1)/Parkin pathway in mammals, selectively removes damaged or redundant mitochondria. Upon mitochondrial depolarization, PINK1 rapidly recruits and activates Parkin to ubiquitinate the mitochondrial membrane. This ubiquitination, identified by p62, facilitates transportation to autophagosomes via LC3, ultimately leading to degradation (Lu et al., 2023). Beyond mitophagy, FOXOs associated with oxidative stress also impact autophagy.

The FOXO transcription factor family regulates various cellular physiological processes such as apoptosis, glucose metabolism, oxidative stress resistance, and lifespan extension (Ou et al., 2021). FOXO1 and FOXO3 are the most widely utilized members of the FOXO family, which can combine with promoter regions to activate autophagy genes such as *Vps34, Beclin-1 and Rab7* (Ferdous et al., 2010; Webb and Brunet, 2014). Furthermore, they directly interact with autophagy proteins such as Atg7 and Atg4 (Cheng, 2019; Zhang X. et al., 2022). AMPK and PI3K/AKT promote FOXO phosphorylation, prompting their translocation from the nucleus to the cytoplasm, impeding normal transcriptional activity and thereby affecting autophagy (Puthanveetil et al., 2013; Zhou G. et al., 2019). Additionally, STRT1’s deacetylation of FOXOs triggers autophagy (Zhou et al., 2021).

#### 3.1.3 Interaction between autophagy and ER stress

Abnormal mitochondrial function and ER stress response are potential causes of insulin resistance (IR) in DMC patients. Autophagy, crucial in maintaining mitochondrial function and influencing ER stability, plays a pivotal role (Jung and Lee, 2010). The ER is the main part of protein folding as well as the main storage organelle of intracellular Ca^2+^. In response to the persistent hyperglycemic state and the corresponding cellular damage, cells in DMC patients, such as pancreatic β cells, produce large amounts of associated stress proteins, including insulin, which exceeds the ability of the ER to eliminate misfolded proteins, leading to the accumulation of a large number of unfolded or misfolded proteins that can trigger the unfolded protein response (UPR), thus resulting in ER stress and even DMC-related cell apoptosis (Yong et al., 2021). The UPR defends cells by degrading misfolded proteins, halting protein translation, and enhancing molecular chaperones for protein folding through endoplasmic reticulum-associated degradation (ERAD), and ER autophagy (inclusive of ER stress-mediated autophagy and ER-phagy) (Yong et al., 2021). UPR is mainly regulated by three ER-transmembrane proteins: PKR-like eukaryotic initiation factor 2α kinase (PERK), inositol-requiring enzyme 1α (IRE1α), and activating transcription factor 6α (ATF6α). PERK phosphorylates the eukaryotic translation initiation factor 2α (eIF2α), curbing protein synthesis and reducing the amount of protein entering the ER. IIRE1α and ATF6α facilitate UPR target gene transcription such as foldase (Park et al., 2018). Autophagy regulation involves PERK’s phosphorylation of eIF2α, ATF4 induction, and increased Atg5 and Atg12 expression, thereby enhancing autophagy. IRE1α activates JNK, phosphorylates anti-apoptotic Bcl-2, ruptures Beclin-1/Bcl-2 complexes, and activates autophagy. ATF6α indirectly triggers autophagy by affecting IRE1α. Furthermore, Ca^2+^ activates the CaMKK/AMPK pathway during ER stress, promoting Beclin-1 dissociation from Bcl-2, thus enhancing autophagy. ER-phagy is a form of selective autophagy that relies on LC3 and Atg8 to eliminate damaged portions of the ER to sustain ER homeostasis (Senft and Ronai, 2015).

#### 3.1.4 Interplay between autophagy and inflammation

Autophagy interacts with inflammation. The cytoplasmic clearance of autophagy exhibits anti-inflammatory effects in any cell capable of activating autonomic inflammation. Moreover, the regulation of mitochondrial and ER function by autophagy affects the functioning of immune cells and influences the onset and resolution of inflammation (Deretic, 2021). Conversely, inflammation can activate or inhibit autophagy through different pathways, resulting in the suppression or exacerbation of inflammatory responses.

Inflammasomes are protein complexes activated by external or internal stimuli and can trigger inflammatory responses. The NLRP3 inflammasome is the most extensively researched inflammasome. In obese mice and humans, the NLRP3 inflammasome is upregulated, leading to the expression of TNF-α, a cytokine that is well-known to promote IR. Thus, NLRP3 inflammasome expression levels correspond with Type 2 diabetes mellitus (T2DM) severity (Komalla et al., 2020; Yang et al., 2020). The production of NLRP3 is closely related to the excessive accumulation of ROS, ER dysfunction, and the corresponding calcium outflow. Autophagy regulates these processes to inhibit the activation of inflammasomes and the pro-inflammatory cytokines IL-1β and IL-18. Specific NLRP3 components targeted by P62/SQSTM1 affect inflammatory mediator expression, thereby controlling inflammation. Studies have shown that NLRP3 production can interact with Beclin-1 to promote LC3-II expression and activate autophagy (Biasizzo and Kopitar-Jerala, 2020). Various inflammatory mediators, including NF-κB, Toll-like receptors, ROS, and Th1 cytokines enhance autophagy. Conversely, Th2 and anti-inflammatory cytokines like IL4 and IL13 inhibit autophagy by activating mTOR (Lapaquette et al., 2015).

In patients with DKD, the accumulation of advanced glycation end products (AGEs) and IL-1β accumulation incite inflammation, exacerbating disease progression. Transcription factor EB (TFEB) is a regulator of lysosomal biogenesis. Autophagy regulates the nuclear translocation of TFEB, enhancing lysosomal biogenesis and stimulating the degradation of AGEs in renal tubular cells (Kimura et al., 2017). In addition, numerous studies have shown that autophagy impairment in retinal cells also activates the inflammasome, leading to DR-related symptoms (Lin and Xu, 2019).

#### 3.1.5 Interplay between autophagy and apoptosis

Type I cell death is the direct result of apoptosis, while type II programmed cell death occurs when autophagy overconsumes most of the organelles. Mitochondrial outer membrane permeabilization (MOMP) is a crucial step in the apoptosis process. Upon external stimulation, the level of anti-apoptotic protein Bcl-2 decreases, leading to the recruitment of pro-apoptotic protein Bax to the mitochondrial membrane that in turn activates MOMP. This results in the release of apoptotic proteins such as cytochrome c (Cyt c), apoptosis-inducing factor (AIF), and downstream-related apoptotic protein family caspase, which are necessary to complete the apoptosis process (Han et al., 2019). Autophagy can work alongside apoptosis to promote cell death. Additionally, autophagy is involved in some ATP-dependent apoptosis processes. However, there is also an antagonistic relationship between autophagy and apoptosis. Autophagy maintains mitochondrial homeostasis, scavenges ROS, reduces ER stress, and mitigates oxidative stress and inflammation to protect cells (D'Arcy, 2019).

Various stress stimuli can co-activate autophagy and apoptosis, and they share multiple regulatory molecules that can even coordinate their transformation. Bcl-2 binds to Beclin-1 to form complexes that inhibit autophagy while maintaining anti-apoptotic effects. However, when Bax competitively binds Bcl-2, Beclin-1 is released, leading to the activation of autophagy. Caspase-3 inactivates Beclin-1 to inhibit autophagy, while Beclin-1 promotes apoptosis by increasing caspase-9 activity. Following cleavage, Atg5 can bind to Bax, inducing the production of cytochrome c and promoting apoptosis. Atg12 can bind to Bcl-2, inhibiting its content and enhancing apoptosis. Additionally, autophagy can eliminate damaged mitochondria, preventing MOMP and the removal of caspase8, which also prevents apoptosis (Mariño et al., 2014).

Research has shown that apoptosis of damaged nerve cells such as Schwann cells is modified following peripheral nerve injury. After treatment, there was an observed upregulation of autophagy, inhibition of apoptosis, and reduction of nerve pain. Proinflammatory cytokines may promote apoptosis and induce nerve pain, while autophagy can inhibit proinflammatory cytokine activity to target these processes (Liao et al., 2022). Excessive apoptosis contributes to coronary atherosclerosis and myocardial ischemic injury. Regulation of autophagy protects cardiomyocytes. Thus, regulation of the relationship between autophagy and apoptosis may be a mechanism by which the progression of heart disease is slowed (Dong Y. et al., 2019).

### 3.2 Autophagy and diabetic kidney disease

DKD is the renal impairment caused by prolonged hyperglycemia which can affect the entire kidney. It is one of the most perilous complications of diabetes. DKD is characterized by impaired glomerular filtration rate (GFR), proteinuria, and structural abnormalities like glomerular basement membrane thickening and mesangial dilatation. The intricate pathogenesis involves apoptosis, inflammation, oxidative stress, ER stress, and nutrient-sensing pathways (Al Mamun et al., 2021). The treatment options for DKD are presently limited (Dorotea et al., 2022). Considerable evidence has shown that autophagy plays a pivotal role in blood sugar stability and renal protection. Targeted autophagy offers potential DKD treatment avenues (Liu et al., 2019; Erekat, 2022).

#### 3.2.1 Podocytes

Podocytes, namely glomerular epithelial cells, play a key role in maintaining glomerular filtration barrier and preventing proteinuria. As terminally differentiated cells, podocytes generally do not replicate, and there are no new cells to repair and replace after injury, which leads to glomerular damage. Hence, autophagy is essential for the maintenance of podocyte homeostasis (Liu et al., 2019). Early in diabetes, autophagy protects podocytes by removing damaged components. Various stress reactions affect autophagy, leading to podocyte damage and DKD development. Experiments have observed changes in autophagy in diabetic foot cells, which provide a basis for this conclusion (Gonzalez et al., 2021).

Research by Jin et al. identified microRNA-486 (miR-486) in adipose-derived stem cell exosomes, inhibiting mTOR via Smad1 downregulation, elevating autophagy, and reducing podocyte apoptosis (Jin et al., 2019). Xiao et al. discovered that after administering the mTOR inhibitor rpapamycin to streptozotocin (STZ)-induced DM mice, there was a significant increase in LC3 expression in podocytes, upregulation of autophagy activity, and reduction in kidney injury (Xiao et al., 2014). Metformin, an AMPK agonist, restores autophagic activity in podocytes, protecting against DM injury (Song A. et al., 2021). Similarly, according to Xu et al., SIRT1/FOXO1 pathways influenced by metformin enhance autophagy, mitigating diabetic kidney injury (Xu J. et al., 2020). Su et al. found that the levels of podocyte autophagy-associated factors (Beclin-1, LC3), as well as the LC3B II/I ratio, decreased with time after high glucose (HG) stimulation and were particularly significant after 24 h. Overexpressed LncRNA AK044604 (regulator of insulin sensitivity and autophagy [Risa]) can reduce the levels of downstream autophagy-related proteins (Beclin-1 and LC3B) through the SIRT1/glycogen synthase kinase-3β (GSK-3β) axis in the HG environment, leading to podocyte damage, thereby accelerating the development of DKD (Su PP. et al., 2022).

#### 3.2.2 Renal tubular epithelial cells

The above-described experimental results confirm that podocyte autophagy will be damaged under long-term HG environment, which ultimately leads to DKD. In addition to podocytes, renal tubular epithelial cells (RTECs) play an important role in maintaining renal function. RTECs are the key part of renal reabsorption activity, and their active transport function consumes a large amount of energy, which makes them more susceptible to hypoxia or energy deprivation. Therefore, maintaining a normal level of autophagy ensures the survival of RTECs in nutrient-poor environments. Experimentally, Atg7 knockout mice demonstrated exacerbated DKD progression, highlighting autophagy’s protective role in RTECs. miR-214 suppression of ULK1 via proximal renal tubule knockout promotes autophagy, reducing renal hypertrophy and albuminuria (Ma et al., 2020). In Liu L. et al., 2022 research, RTECs were one of the most abundant cell types in the mitochondria, so it is more susceptible to mitochondrial disorder. PHB2 is a kind of mitophagy receptor. In STZ-induced DKD mice, PHB2 degradation via TNF-α promoted DKD progression by impairing mitophagy. Kitada et al., 2016 observed that providing obese Wistar rats a very low protein diet could inhibit the mTOR activity of RTECs, reduce renal inflammation, and slows the progression of advanced DKD.

### 3.3 Autophagy and diabetic retinopathy

DR, one of the most common microvascular complications of diabetes, encompasses neurovascular changes leading to irreversible blindness. Today, DR is not just considered a simple microvascular disease, rather one that involves neurodegenerative changes. The American Diabetes Association redefines DR as a neurovascular complication of diabetes. The progression involves pericyte loss, microangiomas, capillary non-perfusion zones, vascular endothelial growth factor (VEGF) secretion, and fragile capillary formation, thereby contributing to vitreous hemorrhage and retinal detachment (Yang et al., 2022). Under the induction of long-term HG level, oxidative stress overload, inflammation, ER stress, and autophagy disorder in the retina lead to retinal microvascular damage and glial cell degeneration, including blood-retinal barrier (BRB) changes, retinal neuron dysfunction and neuronal apoptosis, finally developing into DR (Adornetto et al., 2021).

Autophagy can protect the retina by eliminating ROS, reducing ER stress, apoptosis, and the production of proinflammatory factors (Russo et al., 2013). In fact, neuron cells, podocytes, and RTECs share high similarities. Neurons and podocytes, as highly differentiated cells, cannot undergo the next step of mitosis. At the same time, neurons are metabolically as active as RTECs. Therefore, autophagy disorder is also closely related to neurotoxicity and neurodegeneration. Autophagic activity under mild stress plays a protective role for cell survival, whereas dysregulation of autophagic activity leads to massive cell death, giving rise to the deterioration of DR (Gong et al., 2021).

#### 3.3.1 Blood-retinal barrier

A key feature of DR is the decomposition of the BRB, which is composed of the retinal vascular system (inner layer) and retinal pigment epithelium (RPE) cells (outer layer). The former comprises retinal capillary endothelial cells, peripheral cells, and the basement membrane. The latter is conductive to maintaining retinal fluid balance and photoreceptor function (Gong et al., 2021). After human retinal pigment epithelial cells (ARPE-19) were exposed to HG, treatment with arbutin (a naturally occurring soluble glycosylated phenol) increased SIRT1 expression, enhanced cellular autophagy, and inhibited inflammation and apoptosis (Ma et al., 2021). However, some researchers have found that the inhibition of autophagy can delay DR. Fen et al. found that impairment of autophagic degradation is usually accompanied by the accumulation of p62. The level of p62 in the retina of STZ-induced diabetic rats was significantly higher than that of non-diabetic rats, suggesting errors in autophagic degradation. They suggested that this was caused by the induction of lysosomal membrane permeability (LMP) in HG, resulting in the inability of lysosomes to function properly. Under HG conditions, the downregulation of high mobility group box 1 (HMGB1) rescued LMP, increased mitochondrial electrochemical potential (ΔΨm), restored autophagic degradation, reduced ROS production and VEGF and inflammatory factor expression, and ultimately protected RPE cells (Feng et al., 2022). In another experiment, exposure to HG conditions decreased ARPE-19 cells viability, increased apoptosis and LC3-II/LC3-I, p-p53 protein expression, decreased p62 and p-mTOR expression, and increased autophagic flux. Treatment with proanthocyanidins, a polyphenol compound, weakened the above changes. When rapamycin was added, apoptosis was again increased in the proanthocyanidins-treated ARPE-19 cells, suggesting that increased autophagy in the HG environment impairs ARPE-19 cells and that proanthocyanidins reverse this injury (Li et al., 2022).

#### 3.3.2 Retinal ganglion cells

Retinal ganglion cells (RGCs), the only afferent neurons responsible for transmitting visual information to the visual center, are one of the main cells that maintain visual function. In DR, the levels of p-mTOR and its downstream Ps6 in RGCs were upregulated in STZ-induced mice in the first month, and the cells were slightly lysed. Then, p-mTOR and Ps6 showed a downward trend after 2 months, and their expression reached the lowest level after 6 months, while the number of apoptotic cells peaked. This trend could be changed by adding rapamycin, indicating that mTOR inactivation is an important factor in RGCs damage and that RGCs damage aggravates the course of DR (Madrakhimov et al., 2021). Another study also proved that autophagy damage induced by HG led to RGCs apoptosis and aggravated DR. Compared with the normal group of rat retinal precursor R28 cells, the late apoptosis level in the HG group was significantly higher, and the early apoptosis level in the HG group was also higher than that in normal group (Peng et al., 2022). Most glial cells are Müller cells that exist in all retinal layers; they can provide energy for neurons and are also the main source of VEGF. Wang Y. et al., 2020 showed that the expression of Beclin-1, Atg7, and LC3-IIof Müller cells in the retina of STZ-induced diabetic mice were lower than those in the control group. Autophagy could be promoted by inhibiting the mTOR pathway, which could inhibit the secretion of inflammatory factors and retard the progression of DR. Other studies show that the expression of Beclin-1 and LC3-II were upregulated in Müller cells under HG, which indicated the existence of autophagosomes. However, the subsequent increase of ER stress and the accumulation of p62 indicated that lysosomal function was imbalanced under stress, and the cargos could not be degraded, leading to the release of VEGF and the apoptosis of Müller cells. After 3-methyladenine (3-MA) was used to inhibit autophagy, the number of apoptosed Müller cells increased. On the contrary, after rapamycin was used to restore autophagy degradation and prevent VEGF release from increasing to improve lysosomal proteolytic activity, Beclin-1 was upregulated and the number of apoptosed Müller cells decreased significantly (Lopes de Faria et al., 2016). According to Fu et al.*,* the activation of AMPK after HOG-LDL stimulation in Müller cells led to a significant increase in the levels of autophagy-related proteins Atg5, Beclin-1, and LC3II/LC3I. At the same time, the expression of caspase-3 was further increased, indicating that the activation of autophagy would lead to apoptosis (Fu et al., 2016). In addition, studies have also shown that activation of AMPK can reduce the expression of apoptosis-related proteins Bax, increase Bcl-2, and decrease LC3 expression, and restore autophagy and mitochondrial function to delay DR-induced photoreceptor cell degeneration (Song S. et al., 2021).

### 3.4 Autophagy and diabetic cardiomyopathy

Prolonged diabetes exacerbates diastolic and systolic heart failure post-myocardial infarction, resulting in myocardium-specific microvascular complications, significantly elevating mortality rates among diabetic individuals. DCM, with mild symptoms in the early stage, is difficult to treat in the later stage. Thus, comprehending DCM’s pathogenesis holds significant importance for early diagnosis. Accumulation of ROS is a pivotal pathogenic factor in DCM development, impeding myocardial contractility, and normal cardiac function through redox injury, mitochondrial dysfunction, apoptosis, and myocardial cell fibrosis (Tan et al., 2020). ROS accumulation also prompts aberrant autophagy, crucial for cardiac homeostasis across various cardiovascular cells. Studies indicate abnormal cellular metabolism and accumulation of damaged organelles in DCM rat cardiomyocytes. Similarly, preclinical trials involving DCM patients have demonstrated dysregulated autophagy (Kobayashi and Liang, 2015; Jia et al., 2018). Consequently, autophagy assumes a critical role in DCM. However, the precise influence of autophagy on DCM remains poorly understood. Various researchers have delineated both protective and pathogenic roles of autophagy in type 1 and type 2 diabetes-induced cardiomyopathy, respectively. Autophagy’s role may hinge on diabetes type, stage, and severity (Lee et al., 2018; Dewanjee et al., 2021). Some studies have proposed an “optimal interval” for autophagy in DCM, where optimal therapeutic effects are achieved (Xie et al., 2011; Russo et al., 2012).

#### 3.4.1 Type 1 diabetic heart

In type 1 diabetes mellitus, insulin deficiency hampers signal transduction-regulated glucose and fatty acid transport, leading to cellular starvation, reduced ATP levels, and subsequent AMPK activation initiating autophagy. Mice studies that used chloroquine (CQ) to inhibit autophagy showed cardiomyocyte damage (Kanamori et al., 2015). However, contradictory findings also exist. Xu et al. propose autophagy inhibition as an adaptive response limiting heart damage in type 1 diabetes (Xu et al., 2013). Similarly, Yuan et al. reported alleviated myocardial apoptosis and cardiac fibrosis following CQ-induced autophagy inhibition in mice (Yuan et al., 2016). Thus, further experiments are warranted to elucidate the impact of autophagy in a type 1 diabetic heart.

#### 3.4.2 Type 2 diabetic heart

In contrast to type 1 diabetes, studies suggest that inhibiting myocardial cell autophagy in T2DM can enhance myocardial cell survival. Kanamori et al. found that although the expression of LC3-II was upregulated in db/db mice with T2DM, degenerated mitochondria and autophagosomes were observed in the heart of mice, and neither mature autolysosomes and nor a small amount of lysosomes were detected, which indicated the delay of autophagy flux. Thus, the increase of LC3-II accumulation was considered related to autophagy damage in the last digestive step (Kanamori et al., 2015). Additional research supports these findings. Kobayashi et al. observed reduced autophagic flux in cardiomyocytes cultured in HG, with decreased LC3-II expression. Inhibition of autophagy with 3-MA reduced cardiomyocyte mortality, while upregulating Beclin-1 or Atg7 rendered cardiomyocytes susceptible to HG toxicity (Kobayashi et al., 2012).

Some studies highlight the cardioprotective effects of enhancing autophagy in T2DM. Metformin’s ability to activate AMPK enhances autophagy, reducing myocardial fibrosis and hypertrophy while partially reversing left ventricular dilatation (Kanamori et al., 2019). Zinc supplementation in diabetic rat cardiomyocytes led to reduced LC3-II levels and restored cardiac function, indicating a potential protective role of zinc by inhibiting autophagy (Lu et al., 2015). Chen et al. demonstrated that AMPK pathway activation via the helix B surface peptide (HBSP) in STZ-induced mice increased the LC3-II/LC3-I ratio, reduced p62 levels, inhibited myocardial cell apoptosis, and restored cardiac function. Conversely, using 3-MA diminished the HBSP-induced benefits (Lin et al., 2017).

This conflicting perspective on autophagy-induced DCM represents the previously mentioned “optimal interval.” While autophagy activation is often deemed cardioprotective, excessive autophagy can culminate in cell death. Hence, careful timing and dosage consideration of autophagy as a therapeutic target for DCM are pivotal for optimal therapeutic effects.

### 3.5 Autophagy and diabetic peripheral neuropathy

DPN is a chronic diabetes-related complication characterized by symmetrical distal limb numbness, tingling, muscle weakness, and nociceptive hypersensitivity. As the disease progresses, it can lead to severe consequences such as diabetic foot ulcers, gangrene, and amputation. The neural tissue in patients with DPN undergoes various alterations including hyperglycemia, ROS-induced oxidative stress and inflammation. These changes lead to dysfunction of the mitochondria and ER, which exacerbates the accumulation of harmful substances in neuronal cells and can even lead to apoptosis (Ardeleanu et al., 2020). Neuronal cells, being highly sensitive, are deeply affected by autophagy. Thus, autophagic clearance of harmful neuronal substances remains a key pathway for maintaining neuronal homeostasis.

Schwann cells (SCs) are nerve fibers in the peripheral nervous system that rely heavily on autophagy under normal physiological conditions to eliminate damaged myelinated cell fragments, aiding in nerve regeneration and repair. In DPN, demyelination and SC dysfunction resulting from exposure to an HG environment is noted, which induces critical pathological changes (Wang L. et al., 2020). HG-induced DPN mice and SCs showed elevated ROS levels, reduced LC3-II/LC3-I ratio, elevated p62 expression, and altered myelin thickness; moreover, axon contraction in the sciatic nerve indicated ROS-induced autophagy dysfunction in SCs as a potential cause of DPN (Choi et al., 2023). Yang et al. corroborated decreased autophagosome formation and downregulated sciatic nerve Beclin-1 in STZ-induced rats, correlating with axon end expansion and Purkinje cell degeneration. Adding an autophagy inducer increased Beclin-1 expression in the sciatic nerve, enhancing SCs autophagy, ameliorating myelin degeneration, and alleviating axon atrophy (Yang et al., 2014). Moreover, Yuan et al. revealed decreased Beclin-1 and Atg3 expression in HG-stimulated SCs, yet stilbene lycorine via the AMPK pathway boosted autophagy, slowing DPN progression (Yuan et al., 2022). Additionally, translocator protein (TSPO) agonists displayed therapeutic effects on DPN, although their exact mechanism remains unclear. STZ-induced rats in the control group exhibited reduced Beclin-1 and LC3-II/LC3-I ratios in SCs. TSPO treatment increased Beclin-1 and LC3-II/LC3-I ratios while reducing p62 accumulation. However, these beneficial effects were nullified when using 3-MA, suggesting TSPO’s therapeutic role through autophagy modulation in SCs (Gao et al., 2023).

Some studies highlight that excessive autophagy can induce apoptosis and neuronal damage. In the STZ-induced DPN rat model, the expression of Lipin1 (a phosphatidic acid phosphatase, which is involved in maintaining normal peripheral nerve conduction function) was downregulated, and excessive autophagy was observed, which may lead to the increase of SCs apoptosis and the demyelination of sciatic nerves in DPN rats. Conversely, Lipin1 overexpression curtailed hyperglycemic-induced autophagy hyperactivity and apoptosis, ameliorating sciatic nerve pathology and motor nerve conduction velocity, thereby alleviating DPN (Wang et al., 2021). It is evident that while moderate autophagy exerts a positive influence on nerve repair and regeneration, excessive autophagy results in adverse effects.

## 4 Natural products for regulating diabetic microvascular complications

Owing to the continued explorations of the mechanism of autophagy, its relationship with DMC is progressively becoming clearer. Despite claims of inadequate drug efficacy, investigating NPs with autophagy-regulating abilities offers new avenues for treating DMC. In recent years, NPs like flavonoids (e.g., apigenin, puerarin); polyphenols (e.g., paeonol, resveratrol); alkaloids (e.g., berberine [BBR]); disaccharide (e.g., trehalose); and amines (e.g., melatonin) have exhibited therapeutic potential in diseases via autophagy regulation. However, recognizing autophagy’s bidirectional regulatory effect on diseases mandates stringent evaluation and prediction of autophagy mechanisms, NP dosage, and action timing to ensure positive and stable therapeutic outcomes. This section elaborates on how NPs regulate autophagy through the mTOR, AMPK, and SIRT1 pathways. Additionally, it delves into NPs-mediated autophagy regulation in the mitochondria and ER, along with their roles in reducing oxidative stress, inflammation, and apoptosis.

### 4.1 Natural products and DKD

**FIGURE 4:**
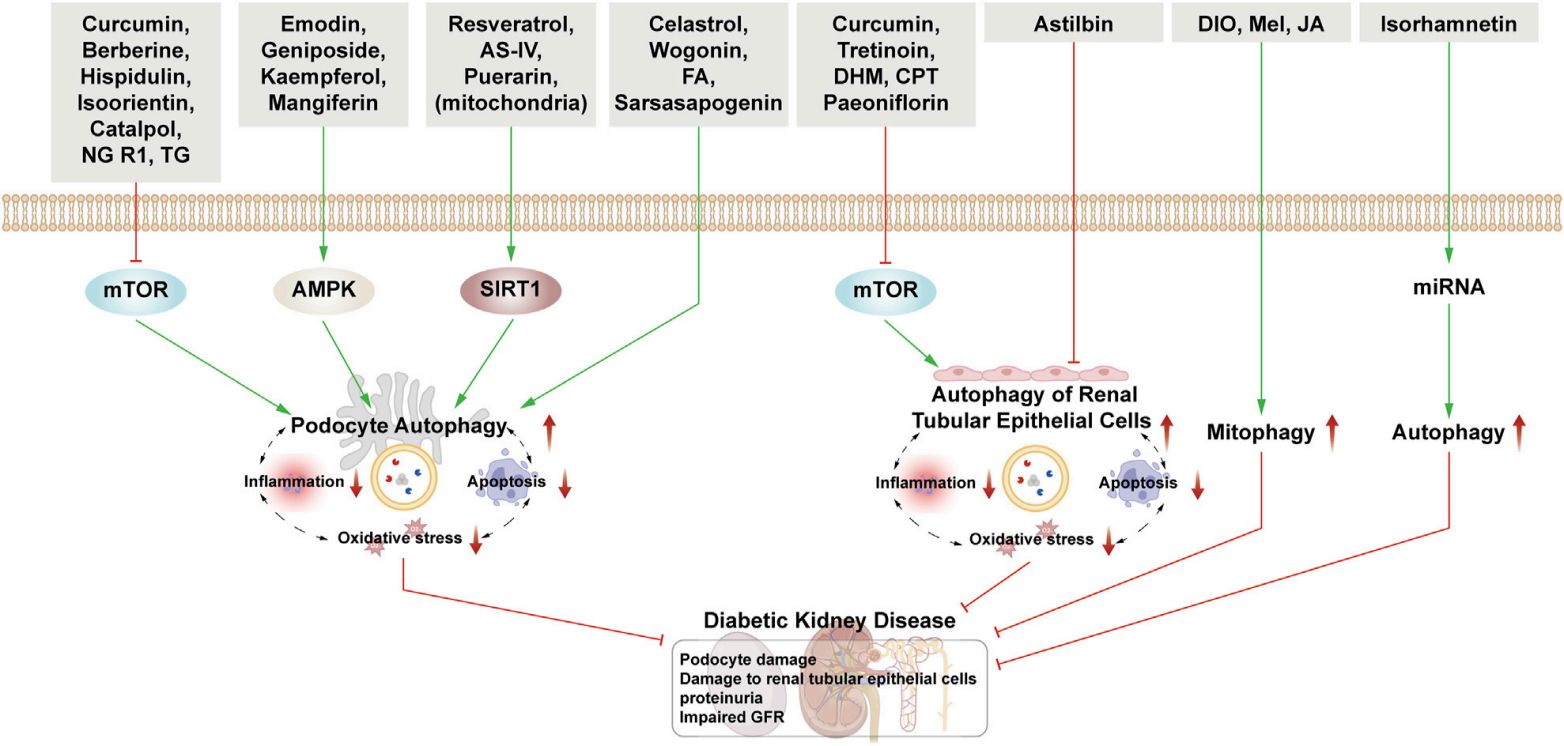
NPs target autophagy to fight DKD. Green arrows indicate downstream cellular events; red lines indicate inhibition. Related NPs target different nutrient-sensing and other pathways to activate autophagy in podocytes and renal epithelial cells, reducing oxidative stress, inflammation, and apoptosis. This helps alleviate the symptoms of DKD.

This section examines the use of NPs to target autophagy as a means of combating DKD. It covers various aspects of autophagy, including podocyte autophagy, renal epithelial cell autophagy, mitophagy, and other autophagic pathways. Additionally, it explores the interplay between autophagy, inflammation, and apoptosis (Figure 4; Table 1).

**TABLE 1:** NPs to target autophagy as a means of combating DKD.

Name	Sources	*In vitro/In vivo*	Model	Dose and duration	Correlated target	Mechanisms	Activation/Inhibition of autophagy	Reference
Curcumin	Turmeric	*In vitro*	MPC5 cells	40 μM 24h	PI3K/AKT/mTOR	↓PI3K/AKT	Activation	Tu et al. (2019)
		*In vivo*	Male SD rats	300 mg/kg 8 weeks		↓mTOR ↓TWIST1 ↑Beclin1, LC3 ↓p62		
Curcumin	Turmeric	*In vitro*	MPC5 cells	20,40,80μM48,78h	Beclin-1/UVRAG/Bcl-2	↑Beclin1, LC3	Activation	Zhang et al. (2020)
						↑UVRAG, Atg5 ↓p62 ↑Bcl-2		
Berberine	Coptis chinensis	*In vitro*	Mouse podocytes	30,60,90 μM 24h	mTOR/P70S6K/4EBP1	↑mTOR	Activation	Li et al. (2020a)
						↑P70S6K/4EBP1		
						↑LC3II/I ↓p62 ↓caspase-3		
Berberine	Coptis chinensis	*In vitro*	MPC5 cells	2.5 μM 20h	AMPK/mTOR	↑AMPK ↓mTOR ↑Beclin-1,LC3II/I ↓p62	Activation	Jin et al. (2017)
Hispidulin	Plantago asiatica	*In vitro*	MPC5 cells	2.5 μM 24h	Pim1/p21/mTOR	↓Pim1/p21	Activation	Wu et al. (2018)
						↓mTOR ↑Beclin-1		
Isoorientin	Fenugreek	*In vitro*	MPC5 cells	40 μM 72h	PI3K/AKT/TSC2/mTOR	↓PI3K/AKT ↓TSC2	Activation	Kong et al. (2023b)
		*In vivo*	C57BL/6J mice	10,20,40 mg/kg 2 months		↓mTOR ↑Beclin-1, LC3II/I ↓p62		
Catalpol	Rehmannia glutinosa	*In vitro*	Mouse podocytes	1,5,10 μM 48h	mTOR/TFEB	↓mTOR ↑TFEB	Activation	Chen et al. (2019b)
		*In vivo*	C57BL/6J mice	30,60,120 mg/kg 8 weeks		↑mRFP		
						↑LC3B		
						↓p62		
NGR1	Panax notoginseng	*In vitro*	Human podocyte	20 μM 24h	PI3K/AKT/mTOR	↓PI3K/AKT	Activation	Huang et al. (2017a)
						↓mTOR ↑Beclin1, LC3II ↓caspase-3, 9		
Tripterygium glycoside	Tripterygium	*In vitro*	MPC5 cells	1.25 μM	β-arrestin-1	↓β-arrestin-1	Activation	Zhan et al. (2019)
				24h		↑LC3II/I		
						↓p62		
Tripterygium glycoside	Tripterygium	*In vitro*	MPC5 cells	1.25 μM	mTOR/Twist1	↓mTOR	Activation	Tao et al. (2021)
				24h		↓Twist1 ↑Beclin1,LC3II/I ↓EMT,N-cadherin		
Emodin	Rhubarb	*In vivo*	Male SD rats	20,40 mg/kg 8 weeks	AMPK/mTOR	↑AMPK ↓mTOR ↑Beclin-1,LC3II/I ↓p62	Activation	Liu et al. (2021)
Geniposide	Gardenia	*In vivo*	C57BL/6J mice	50 mg/kg	AMPK/AKT	↑AMPK ↓AKT ↑Beclin-1,LC3II/I ↓PGC-1α	Activation	Dusabimana et al. (2021)
				5 weeks		↓TNFα		
Kaempferol	broccoli	*In vivo*	db/db mice	50,100 mg/kg	AMPK/mTOR	↑AMPK ↓mTOR ↑Beclin-1, LC3II ↓p62 ↑Bcl-2	Activation	Sheng et al. (2022)
				12 weeks		↓Bax,caspase-3		
Mangiferin	Mangifera indica L	*In vivo*	Male SD rats	12.5,25,50 mg/kg	AMPK/mTOR	↑AMPK ↓mTOR	Activation	Wang et al. (2018b)
				12 weeks		↑p-ULK1		
						↑Beclin-1, LC3II ↓p62		
Resveratrol	Grape leaves	*In vivo*	Male SD rats	5 mg/kg	SIRT1	↑SIRT1	Activation	Ma et al. (2016)
				4 months		↑Beclin-1,LC3II/I ↓p62 ↑Atg5,7		
						↓TNFα,IL-6		
Resveratrol	Grape leaves	*In vitro*	Human podocytes	0,5,10,15 μM 48h	miR-383-5p	↓miR-383-5p	Activation	Gowd et al. (2020)
		*In vivo*	db/db mice	10 mg/kg		↑Beclin-1, LC3II ↓p62		
				12 weeks		↑Atg5		
						↓Bax,caspase-3		
Resveratrol	Grape leaves	*In vitro*	Mouse podocytes	1,10,100 μM 12h	miR-18a-5p	↑miR-18a-5p	Activation	Huang et al. (2017b)
		*In vivo*	db/db mice	100 mg/kg		↓ATM		
				12 weeks		↑Beclin-1,LC3II/I ↓p62		
AS-IV	Astragalus	*In vitro*	Mouse podocytes	20,50,100 μM 48h	SIRT1/NF-κB	↑SIRT1/NF-κB	Activation	Wang et al. (2019c)
		*In vivo*	Polygenic KK-Ay mice	40 mg/kg		↑Beclin-1, LC3II ↓ECM		
				12 weeks				
AS-IV	Astragalus	*In vivo*	C57BL/6J mice	3,6,12 mg/kg	SERCA2b, AMPK	↑SERCA2b	Activation	Guo et al. (2017)
				8 weeks		↑AMPK		
						↑Beclin-1,LC3II/I ↓ATF6,PERK, ↓IRE1α, p-eIF2α		
						↓caspase-3,12		
Puerarin	Radix puerariae	*In vivo*	C57BL/6J mice	5,10,20,40 mg/kg	HMOX-1, SIRT1/AMPK	↑HMOX-1	Activation	Li et al. (2020b)
				12 weeks		↑SIRT1/AMPK		
						↑Beclin-1,LC3II/I		
Puerarin	Radix puerariae	*In vivo*	C57BL/6J mice	40,80 mg/kg	PERK/eIF2α/ATF4	↑PERK/eIF2α ↑ATF4	Activation	Xu et al. (2020b)
				8 weeks		↑Beclin-1,LC3II/I		
						↑Atg5 ↓p62		
Celastrol	Tripterygium	*In vitro*	Mouse podocytes	0.1, 0.2, 0.6, 1.0,1.5,2μM5h	HO-1	↑HO-1	Activation	Zhan et al. (2018)
						↑Beclin-1,LC3II/I		
						↓p62		
						↓TNFα,IL-6		
Ferulic acid	Many plants	*In vivo*	C57BL/6J mice	200 mg/kg	Beclin-1, NLRP3	↑Beclin-1,LC3II/I	Activation	Ma et al. (2022)
				8 weeks		↓p62		
						↓NLRP3,IL-1β		
Cordyceps Militaris Polycystide	Cordyceps militaris	*In vivo*	C57BL/6J mice	200,400 mg/kg	Beclin-1, NLRP3	↑Beclin-1,LC3	Activation	Chen et al. (2019a)
				6 weeks		↑Atg5 ↓p62 ↓MCP-1,IL-1β		
Wogonin	Scutellaria baicalensis Georgi	*In vitro*	MPC5 cells	4,8,16Μm	Bcl-2	↑Bcl-2	Activation	Liu et al. (2022b)
				24h		↑Beclin-1,LC3		
		*In vivo*	C57BL/6J mice	10,20,40 mg/kg		↑Atg7 ↓p62		
				12 weeks		↓Bax,caspase-3		
						↓TNFα,IL-6		
Sinensetin	Citri Reticulatae Pericapium	*In vitro*	MPC5 cells	80 µM 72h	LC3, P62	↑LC3	Activation	Kong et al. (2023a)
		*In vivo*	C57BL/6J mice	10,20,40 mg/kg		↓p62		
				8 weeks				
Sarsasapogenin	Anemarrhena asphodeloides Bunge	*In vitro*	MPC5 cells	20,40 µM 24h	GSK-3β	↓pTyr216-GSK3β	Activation	Li et al. (2021c)
		*In vivo*	Male SD rats	20,60 mg kg		↑pSer9- GSK3β		
				10 weeks		↑Beclin-1,LC3		
						↑Atg5		
						↓p62		
Ginsenoside Rg1	Ginseng	*In vitro*	MPC5 cells	40 µM 48h	AKT/GSK-3β/β-catenin	↑AKT/GSK-3β	Activation	Shi et al. (2020)
		*In vivo*	Male SD rats	50 mg/kg		↓β-catenin		
				8 weeks		↑Beclin-1,LC3Ⅱ		
						↓p62		
Curcumin	Turmeric	*In vitro*	NRK-52E cells	10 µM 48h	PI3K/AKT	↑PI3K/AKT ↑Beclin-1,LC3II/I	Activation	Wei et al. (2017b)
						↓p62		
						↓Bax,caspase-3		
Tretinoin	Tripterygium	*In vitro*	HMCs	10 µM 48h	miR-141-3p/PTEN/Akt/mTOR	↓miR-141-3p	Activation	Li et al. (2017b)
		*In vivo*	Male SD rats	200 μg/kg		↑PTEN		
				12 weeks		↓Akt/mTOR		
						↑LC3 ↓p62		
						↓RIF		
DHM	Ampelopsis Michx	*In vitro*	NRK-52E cells	1 μM	miR-155-5p/PTEN	↓miR-155-5p	Activation	Guo et al. (2020a)
				24h, 48h	PI3K/AKT/mTOR	↑PTEN		
		*In vivo*	Male SD rats	100 mg/kg		↓AKT/mTOR		
				10 weeks		↑Beclin-1,LC3II/I ↓p62 ↓RIF		
CPT	Cyclocarya paliurus	*In vitro*	HK-2 cells	5.20 μM	AMPK/mTOR	↑AMPK/mTOR	Activation	Zhang et al. (2019)
				48h		↑Beclin-1,LC3II/I ↓p62		
		*In vivo*	Male SD rats	40,160 mg/kg		↓caspase-3		
				10 weeks		↓RIF		
Asiatic acid	Cyclocarya paliurus	*In vitro*	HK-2 cells	1.5 μM	TGFβ1/smad3	↓TGFβ1/smad3	Activation	Zhang et al. (2022c)
				24h		↑LC3,LAMP1		
		*In vivo*	Male SD rats	10,30 mg/kg		↓p62		
				15 weeks		↓α-SMA		
						↓EMT		
Syringic acid	Many plants	*In vitro*	NRK- 52E cells	10,20 μM	Nrf2	↑Nrf2,NQO-1	Activation	Sherkhane et al. (2023)
				48h		↑Beclin-1,LC3		
		*In vivo*	Male SD rats	20,50 mg/kg		↑Atg3,5,7		
				4 weeks		↓p62		
						↓ROS		
Astilbin	Glabrous greenbrier rhizome	*In vitro*	HK-2 cells	10,20 μM	PI3K/AKT	↑PI3K/AKT	Inhibition	Chen et al. (2018a)
				24h		↓Beclin-1,LC3II/I		
						↑p62		
						↓Bax,caspase-3		
Paeoniflorin	Radix Paeonia rubra	*In vitro*	Rat glomerular mesangial cells	25,50 μM	RAGE, mTOR	↓RAGE ↑mTOR	Inhibition	Chen et al. (2017)
				24h		↓Beclin-1,LC3II/I		
						↑p62		
Diosgenin	Rhizoma dioscoreae	*In vivo*	Male SD rats	20 mg/kg	PINK1/Parkin, AMPK/mTOR	↑PINK1/Parkin	Activation	Zhong et al. (2022a)
				8 weeks		↑AMPK/mTOR		
						↓PERK/eIF2α ↓ATF4,CHOP		
						↑Beclin-1,LC3		
						↓Bax,caspase-12		
Diosgenin	Rhizoma dioscoreae	*In vitro*	HK-2 cells	1,2,4 μM	CaMKK2, PINK1/Parkin, AMPK/mTOR	↑CaMKK2, ↑PINK1/Parkin	Activation	Zhong et al. (2023)
				24h		↑AMPK/mTOR		
		*In vivo*	Male SD rats	10,20 mg/kg		↓PERK/eIF2α ↓ATF4,CHOP		
				8 weeks		↑Beclin-1,LC3		
						↓Bax,caspase-12		
Jujuboside A	Jujube	*In vivo*	Male SD rats	20 mg/kg	CaMKK2, PINK1/Parkin, AMPK/mTOR	↑CaMKK2, ↑PINK1/Parkin	Activation	Zhong et al. (2022b)
				8 weeks		↑AMPK/mTOR		
						↓PERK/eIF2α ↓ATF4,CHOP		
						↑Beclin-1,LC3		
						↓Bax,caspase-9		
Melatonin	Pineal body	*In vitro*	HK-2 cells	100 μM	PINK1/Parkin, AMPK	↑PINK1/Parkin	Activation	Tang et al. (2022)
				24h		↑AMPK		
		*In vivo*	C57BL/6J mice	0.2 mg/kg		↑LC3 ↓p62		
				12 weeks		↓α-SMA		
						↓TNFα,IL-1β		
Isorhamnetin	Hippophae rhamnoides L	*In vivo*	Male Wistar rats	50 mg/kg	miR-15b, miR-34a, miR-633	↓miR-15b ↓miR-34a ↓miR-633	Activation	Matboli et al. (2021)
				4.8 weeks		↑ULK1, WIPI ↑FYCO1,TECPR		
						↑LC3II/I ↓p62		

#### 4.1.1 Natural products mediate podocyte autophagy to alleviate renal injury

##### 4.1.1.1 Podocyte autophagy regulated by the mTOR pathway

Curcumin is derived from *Curcuma longa* and exhibits anti-inflammatory and anti-cancer properties. Podocyte epithelial mesenchymal transition (EMT) causes podocytes to fall off and be eliminated from the body. When EMT occurs, it is accompanied by the decrease of epithelial marker E-cadherin. In DKD rats, curcumin treatment significantly reduces blood glucose, urinary protein, and urea nitrogen levels. On a molecular level, it increases E-cadherin and LC3 protein expression while decreasing PI3K, p-Akt, and TWIST1. The expression of these proteins was reversed by the application of mTOR inducers. Therefore, it is suggested that curcumin can induce autophagy and reduce the changes of EMT in podocytes through the PI3k/Akt/mTOR pathway to treat DKD (Tu et al., 2019). Furthermore, Zhang et al., 2020 proved that after curcumin treatment, the expression of LC3, Beclin-1, UVRAG, ATG5, and Bcl-2 increased, while the expression of Bax and caspase-3 decreased, indicating that curcumin promoted podocyte autophagy and inhibited podocyte apoptosis by regulating Beclin-1/UVRAG/Bcl-2.

Berberine, a quaternary alkaloid isolated from *Berberis vulgaris*, is widely distributed in the plant kingdom and has antibacterial, anti-inflammatory, and antitumor properties. In HG-cultured mouse podocytes clone 5 (MPC5) cells, BBR activates autophagy by inhibiting the mTOR/P70S6K/4EBP1 signaling pathway, thereby safeguarding podocytes (Li C. et al., 2020). Moreover, BBR’s protective effect on podocytes involves increased Beclin-1 expression, LC3II/LC3I ratio, and autophagosome count via AMPK activation and mTOR inhibition. This effect is attenuated after 3-MA treatment, affirming BBR’s protective action on podocytes through autophagy (Jin et al., 2017).

Hispidulin is a natural flavonoid mainly extracted from *Artemisia pilosula*. Hispidulin possesses anti-inflammatory, hypoglycemic, and anti-angiogenesis properties. It induces autophagy by activating the Pim1/p21/mTOR signal axis, thereby mitigating HG-induced podocyte damage (Wu et al., 2018). Isoorientin, a flavonoid derived from the leaves of Bamboo and Charcot, has anti-oxidant and anti-inflammatory properties and improves IR. Under HG conditions, isoorientin stimulates autophagy via the PI3K/AKT/TSC2/mTOR pathway, significantly improving the damaged mitochondria’s autophagic clearance rate to protect podocytes. (Kong et al., 2023b).

Catalpol is a natural iridoid glycoside compound derived from Chinese medicinal herb *Rehmannia glutinosa*. It relieves renal pathological damage in DKD mice and rescues foot cytoskeletal destruction induced by HG. Catalpol enhances autophagy by inhibiting mTOR activity and promoting TFEB nuclear translocation, thus stabilizing podocyte cytoskeleton (Chen Y. et al., 2019). Notoginsenoside R1 (NGR1), derived from the dried roots and rhizomes of *Panax notoginseng*, is also a terpenoid. NGR1 protects podocytes from HG-induced injury by increasing autophagy, inhibiting apoptosis, and promoting cytoskeletal restoration via activation of the PI3K/Akt/mTOR pathway (Huang G. et al., 2017).

Tripterygium glycoside (TG), one of the active substances extracted from *Tripterygium*, has immunosuppressive and anti-inflammatory properties and has been used to reduce proteinuria in DKD patients. Zhan et al. showed that the effect of TG on podocytes is via both the activation of autophagy and the downregulation of β-arrestin-1 (Zhan et al., 2019). Mei et al. reported that overexpression of Twist1 could aggravate podocyte damage, and the podocyte damage was relieved after TG treatment. After adding mTORC1 activator, its therapeutic effect on podocytes was reversed. Therefore, TG can also activate autophagy through mTOR/Twist1 signaling pathway to alleviate EMT and podocyte apoptosis (Tao et al., 2021).

##### 4.1.1.2 Podocyte autophagy regulated by AMPK pathway

Emodin is extracted from *Pinelliae Rhizoma* and *Palmariae Rhizoma*, and it reduces proteinuria and mitigates podocyte foot process fusion in DKD rats. Treatment with emodin increases LC3-II/I, p-AMPK, and Beclin-1 while reducing p62 and p-mTOR expression, suggesting rhodopsin-induced podocyte protection through enhanced autophagy via the AMPK/mTOR pathway (Liu et al., 2021).

Geniposide extracted from *Gardenia* is used to treat cardiovascular and cerebrovascular diseases as well as diabetes. In DKD mice, geniposide reduces podocyte loss, improves renal function, increases AMPK activity, and enhances autophagy. Moreover, it mitigates oxidative stress and inflammation in the kidney by reducing AKT activity (Dusabimana et al., 2021).

Kaempferol is a flavonoid and is widely present in fruits and vegetables. It alleviates mesangial matrix expansion and podocyte loss or fusion in DKD. It slows DKD progression by upregulating podocyte autophagy via the AMPK/mTOR pathway, evidenced by increased LC3II, Beclin-1, p-AMPK, Bcl-2, Atg7, and Atg5, and decreased p-mTOR, Bax, caspase-3, and p62 expression (Sheng et al., 2022). Mangiferin and phenolics from *Physalis peruviana* fruits have both been shown to exert a protective effect on the kidney by enhancing autophagy through the AMPK/mTOR pathway (Wang X. et al., 2018; Ezzat et al., 2021).

##### 4.1.1.3 Podocyte autophagy regulated by SIRT1 pathway

Resveratrol (RSV), a non-flavonoid polyphenol, can be synthesized in the leaves and skins of grapes and has antioxidant and anticancer properties and offers cardiovascular protection. RSV, as an activator of SIRT1 and AMPK, promotes the expression of SIRT1 under hypoxic conditions, up-regulating the expression of LC3, Atg7, and Atg5, and activates hypoxia-induced autophagy (Ma et al., 2016). Interestingly, RSV can inhibit ER stress by reducing the production of ROS and AGEs and activate AMPK to promote autophagy (Gowd et al., 2020). According to Huang et al. and Xu et al., RSV can inhibit miR-383-5p and upregulate miR-18a-5p, resulting in an increase in the ratio of LC3II to LC3I and a decrease in the expression of caspase-3 in podocytes, to inhibit foot cell apoptosis. The above protective effect of RSV can be inhibited by 3-MA, which further proves that RSV exerts therapeutic effect in DKD through autophagy (Huang SS. et al., 2017; Xu et al., 2017).

Astragaloside IV (AS-IV), extracted from *Astragalus membranaceus*, has good anti-virus and hypoglycemic effects. After intervention with astragaloside IV, the expression of SIRT1 is increased and the acetylation of NF-κB subunit p65 is decreased, reversing the EMT of podocyte, enhancing autophagy and protecting podocyte (Wang X. et al., 2019). Guo et al. also found that astragaloside IV improved the expression of sarcoendoplasmic reticulum Ca^2+^ ATPase 2b (SERCA2b) and activated AMPK, improving ER stress, activating autophagy and preventing the progress of DKD (Guo et al., 2017).

Puerarin extracted from radix puerariae, a leguminous plant, is often used in patients with coronary heart disease and hypertension. On the one hand, puerarin upregulates autophagy through HMOX1 and SIRT1, protecting podocytes and delaying the progression of DKD (Li X. et al., 2020). On the other hand, under conditions of ER stress, puerarin upregulates the PERK/eIF2α/ATF4 signaling pathway; increases the expression of Beclin-1, LC3II, and Atg5; downregulates the expression of p62, and alleviates renal injury (Xu X. et al., 2020).

##### 4.1.1.4 Podocyte autophagy regulated by other pathways

Celastrol, another natural compound extracted from *Tripterygium*, activates heme oxygenase-1 (HO-1) expression, thus increasing LC3-II expression; activates autophagy; and reduces apoptosis, inflammatory reaction, and IR of lower podocytes induced by HG (Zhan et al., 2018).

Ferulic acid (FA) is a phenolic acid widely existing in plants. After intervention with FA, a series of clinical indices of DKD mice showed improvement, wherein the expression of LC3 was upregulated and the expression of p62 and interleukin-1β (IL-1β) were inhibited, confirming that FA plays a therapeutic role in DKD by increasing autophagy and inhibiting inflammation (Ma et al., 2022).

After the treatment of DKD mice with Cordyceps militaris polysaccharides, the autophagy rate was increased, and the expression of Atg5, Beclin-1, and LC3 proteins increased, thus promoting the recovery of renal function (Chen DD. et al., 2019).

Wogonin is a flavonoid from the root of Scutellaria baicalensis Georgi. Scutellarin promotes autophagy by regulating the expression of Bcl-2, inhibiting apoptosis of MPC5 cells induced by HG, and relieving glomerular injury (Liu XQ. et al., 2022). Sinensetin is a flavonoid compound existing in Citri Reticulatae Pericarpium. Treatment with sinensetin results in the upregulation of LC3 expression and downregulation of p62 expression in MPC5 cells under HG conditions, and the activation of autophagy significantly increases the viability of podocytes (Kong et al., 2023a).

Experiments have further confirmed the therapeutic effect of stimulating the GSK-3β signaling pathway in DKD. Sarsasapogenin, one of the main active components of lily, restores podocyte autophagy by activating the GSK-3β signal pathway (Li XZ. et al., 2021). Ginsenoside Rg1, one of the active components of ginseng, can alleviate EMT by enhancing autophagy mediated by the AKT/GSK-3β/β-catenin pathway (Shi et al., 2020).

#### 4.1.2 Natural products’ intervention in the autophagy of RTECs to alleviate renal injury

Curcumin not only acts on podocytes but also participates in the regulation of epithelial cells. Curcumin upregulates autophagy by inducing the PI3K/AKT pathway in rat epithelial cells (NRK-52E), which reverses the increase of apoptosis of epithelial cells after AGEs treatment, and thus plays a renoprotective role (Wei Y. et al., 2017).

Tretinoin is a natural compound extracted from *Tripterygium*. TG and celastrol exert their therapeutic effects on DKD mainly by affecting autophagy in podocytes, while triptolide plays a role by restoring autophagy in human mesangial cells (HMCs) induced by HG and reducing fibrosis. The mechanism is mainly related to inhibition of the miR-141-3p/PTEN/Akt/mTOR pathway (Li XY. et al., 2017).

Dihydromyricetin (DHM), a flavonoid, is the main active ingredient in Rattan tea. In DKD rats and HG-induced NRK-52E cells, DHM regulates the miR-155-5p/PTEN signaling pathway and the PI3K/AKT/mTOR signaling pathway to promote autophagy and attenuate renal interstitial fibrosis (Guo L. et al., 2020).

*Cyclocarya paliurus* (CP), the fraction of which enriches triterpenic acids (CPT) alleviates renal injury by regulating the AMPK/mTOR pathway and activating autophagy (Zhang et al., 2019). In later research, Zhang et al., 2022c also found that asiatic acid (a further extract of CPT) in DKD rats and HG-induced human RTECs (HK-2) inhibited transforming growth factor-b type I receptor (TGF-bRI) and activated the autophagy-lysosomal system, thus altering the progression of EMT and inhibiting renal tubulointerstitial fibrosis.

Syringic acid exists in many kinds of dried fruits and plants. In DKD rats and NRK-52E cells, syringic acid increases the expression of Atg3, Atg5, and Atg7 and improves the level of Nrf2 in diabetic rats, to exert renoprotective effects (Sherkhane et al., 2023).

Some NPs have been used in the treatment of DKD by inhibiting the activity of autophagy. Astilbin is extracted from the Glabrous Greenbrier Rhizome, which has hypoglycemic and antioxidant effects. In HG-induced HK-2 cells, astilbin inhibits autophagy and apoptosis in HK-2 cells through the PI3K/Akt pathway (Chen F. et al., 2018). Paeoniflorin, isolated from radix Paeonia rubra and the root of herbaceous peony, has antioxidant, anti-platelet aggregation, and anti-inflammatory effects. Paeoniflorin inhibits autophagy by inhibiting the receptor for advanced glycation endproducts (RAGE) and up-regulating p-mTOR levels to counteract mesangial cell dysfunction induced by AGEs (Chen et al., 2017).

#### 4.1.3 Natural products interfere with mitophagy to alleviate renal injury

Diosgenin (DIO), an important basic raw material for the production of steroid hormone drugs, is widely found in leguminous and *Dioscoreaceae* plants. Mitophagy is mediated by PINK1-PARKIN. DIO can reverse disorders of the mitochondrial respiratory chain; enhance the activity of antioxidant enzymes; and downregulate the expression of mitochondrial apoptosis proteins Bax, CytC, Apaf-1, and caspase-9 and that of ER-related kinases p-PERK, p-IRE1, and ATF4. This results in inhibition of apoptosis caused by mitochondrial and ER stress. DIO also enhances autophagy through the AMPK-mTOR pathway (Zhong et al., 2022a). In subsequent experiments, they further proposed that DIO targeted CaMKK2 to regulate the AMPK-mTOR and PINK1-Parkin pathways to improve autophagy and mitophagy to alleviate the progression of DKD (Zhong et al., 2023). Jujuboside A is a glycoside isolated from the seeds of jujube, and its therapeutic mechanism for DKD is the same as that of Diosgenin, which reaffirms that improvement of mitophagy and ER stress can play a therapeutic role in DKD (Zhong et al., 2022b).

Melatonin (Mel) is an indole heterocyclic compound produced by the pineal gland that scavenges ROS accumulation and has a potent antioxidant capacity. Mel promotes AMPK, PINK1, and Parkin activation, thereby activating mitophagy. Therefore, Mel protects the kidney through the enhancement of mitophagy, reduction of oxidative stress, and inhibition of inflammation (Tang et al., 2022).

#### 4.1.4 Natural products interfere with autophagy through other ways to alleviate renal injury

Epigenetic modulation of non-coding RNAs plays a key role in the pathogenesis of diabetes and kidney injury, and in pancreatic β-cells of DM patients, several miRNAs such as miR-633 are dysregulated. As mentioned above, RSV (Huang SS. et al., 2017; Xu et al., 2017) which affects podocyte autophagy, as well as ryanodine (Li XY. et al., 2017) and DHM (Guo L. et al., 2020) that affect epithelial cell autophagy, all involve miRNA regulation. Derived from Hippophae rhamnoides L., isorhamnetin administration in DKD rats enhances LC3 II/I protein expression and autophagosome count, improving renal conditions. WIPI and FYCO1 are involved in the PI3K complex formation during nucleation, and TECPR is involved in autophagosome-lysosome fusion. By reducing miR-15b, miR-34a, and miR-633 expressions, isorhamnetin increases downstream autophagy transcription signals ULK1, WIPI, FYCO1, and TECPR mRNA expression, thereby bolstering the protective role of autophagy (Matboli et al., 2021)

### 4.2 Natural products and diabetic retinopathy

**FIGURE 5:**
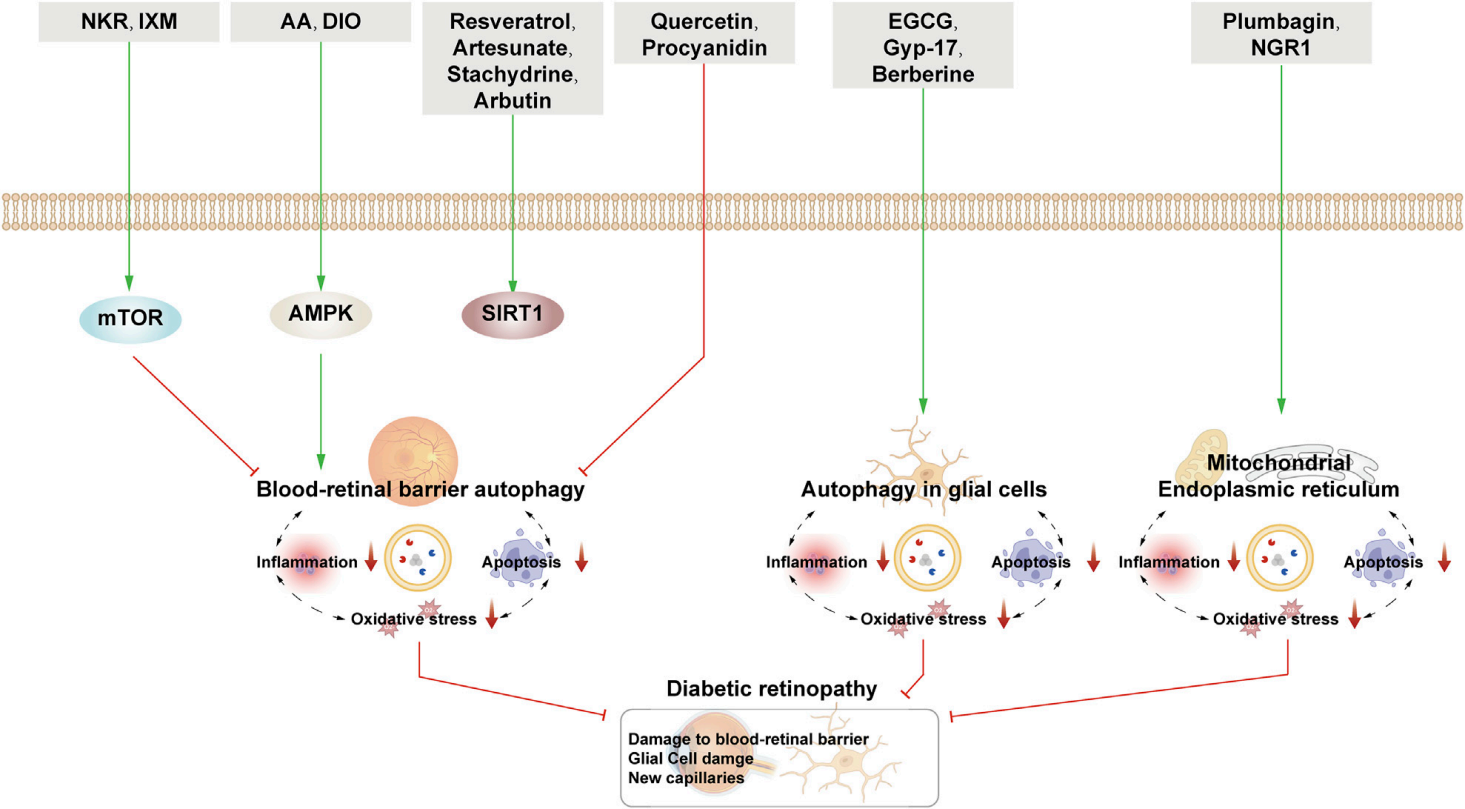
NPs target autophagy to fight DR. Green arrows indicate downstream cellular events; red lines indicate inhibition. Related natural products target different nutrient-sensing pathways and other pathways, activate BRB autophagy, and autophagy in glial cells and ER autophagy and reduce oxidative stress, inflammation, and apoptosis, thus alleviating the symptoms of DR.

This section examines the use of NPs to target autophagy as a means of combating DR. It covers various aspects of autophagy, including autophagy of the BRB, glial cell autophagy, and mitophagy. Additionally, it explores the interplay between autophagy, inflammation, and apoptosis (Figure 5; Table 2).

**TABLE 2:** NPs to target autophagy as a means of combating DR.

Name	Sources	*In vitro*/*In vivo*	Model	Dose and duration	Correlated target	Mechanisms	Activation/Inhibition of autophagy	Reference
Norkurarinone and Isoxanthohu-mol	Sophora flavescens Aiton	*In vitro*	HRMECs	15,20,40,80 μM 24,48h	PI3K/AKT/mTOR	↓PI3K/AKT	Inhibition	Zhao et al. (2022)
						↓mTOR ↓Beclin-1,LC3Ⅱ ↓ATG5 ↑P62 ↓ROS		
Arjunolic acid	Cyclocarya paliurus	*In vitro*	ARPE- 19 cells	5.10 μm 24h	AMPK/mTOR/HO-1	↑AMPK,HO-1 ↓mTOR, p62 ↑Beclin-1, LC3II/I ↓TNF-α,IL-1β ↓Bax,caspase-3	Activation	Zhang et al. (2022b)
		*In vivo*	Male SD rats	30 mg/kg 10 weeks				
Diosgenin	Rhizoma dioscoreae	*In vitro*	ARPE- 19 cells	10,50,100 μM 24h	AMPK/Nrf2/HO-1	↑AMPK,HO-1 ↑Nrf2 ↑Beclin-1,LC3II/I ↑SOD,GSH-Px ↓IL-1β,TNF-α ↓Bax, caspase-3	Activation	Hao and Gao (2022)
Resveratrol	Grape leaves *In vitro*	*In vitro*	Bovine REC	20 μm	AMPK/SIRT1/PGC-1α	↑AMPK/SIRT1 ↑PGC-1α ↓caspase-3 ↓ROS	Activation	Li et al. (2017a)
				48h				
Artesunate	Artemisinin	*In vivo*	Male SD rats	intravitreal injection	AMPK/SIRT1	↑AMPK, SIRT1 ↑Beclin-1, LC3II/I ↓p62 ↓ROS ↓IL-6,MCP-1	Activation	Li et al. (2023)
				2 or 10 lg in 1 lL vehicle				
Stachydrine	Leonurus heterophyllus	*In vitro*	HRMECs	6.25,12.5.25 μM	AMPK/SIRT1	↑AMPK, SIRT1 ↑Beclin-1, LC3II/I ↓p62 ↓TNF-α,IL-1β ↓ROS	Activation	Yu et al. (2023)
				24h				
		*In vivo*	Male SD rats	40 mg/kg 2 weeks				
Arbutin	Bearberry	*In vitro*	ARPE- 19 cells	25,50,100 μM 24,48h	SIRT1	↑SIRT1	Activation	Ma et al. (2021)
						↑Beclin-1, LC3II/I		
						↓Bax,caspase-3 ↓TNF-α,IL-1β		
Melatonin	Pineal body	*In vitro*	HRMECs	100 μM 24h	Wnt/β-catenin	↑Beclin-1,LC3II/I ↓p62 ↓IL-1β,TNF-α	Activation	Dehdashtian et al. (2018)
		*In vivo*	Male SD rats	10 mg/kg 3 weeks				
Quercetin	Apple	*In vitro*	HRMECs	20,40,80 μM	NLRP3	↓NLRP3, caspase1 ↓Beclin-1, LC3 ↓IL-1, IL-18	Inhibition	Li et al. (2021b)
				48h				
Procyanidin	Grape seeds	*In vitro*	ARPE- 19 cells	10 μM 48h	P53/mTOR	↓p53 ↑p-mTOR, p62 ↓LC3II/I ↓Bax, caspase-3	Inhibition	Li et al. (2022)
Epigallocatechin-3- gallate	Green tea	*In vitro*	Müller cell	20 μm 24h	mTOR	↓mTOR ↓p62	Activation	Wang et al. (2019a)
						↑Beclin-1, LC3II ↓Caspase-3		
Gypenoside XVII	GynOstemma	*In vivo*	db/db mice	10,20 mg/kg 5 weeks	Beclin-1, LC3-II	↑Beclin-1,LC3-II ↑ATG-5,Bcl-2 ↓p62 ↓Bax,caspase-3 ↑SOD,GSH-px	Activation	Luo et al. (2021)
Berberine	Coptis chinensis	*In vitro*	Müller cell	2.5,5,10,20 μM 48h	AMPK/mTOR	↑p-AMPK ↓p-mTOR ↑Beclin-1,Bcl-2 ↓Bax,caspase-3	Activation	Chen et al. (2018b)
NGR1	Notoginseng	*In vitro*	Müller cell	5,10,20,40 μM 24h	PINK1/Parkin	↑PINK1/Parkin ↑LC3II/I ↓p62/SQSTM1 ↓ROS,4-HNE	Activation	Zhou et al. (2019b)
		*In vivo*	db/db mice	30 mg/kg 12 weeks				
Plumbagin	Plumbago zeylanica	*In vivo*	Female and male adult *D. melanogaster*	30 nM 10 days	LC3-II, Nrf2	↑LC3II ↓p62 ↓caspase-3 ↓ATF4 ↓ROS, Nrf2	Activation	Catalani et al. (2023)

#### 4.2.1 Natural products interfere with autophagy of the BRB to alleviate retinal damage

##### 4.2.1.1 Autophagy of retinal vascular cells regulated by the mTOR pathway

Norkurarinone and isoxanthohumol are the ethyl acetate extracts of *Sophora flavescens Aiton*. In addition, these compounds can also inhibit cell migration and the generation of tubular structure of human retinal microvascular endothelial cells (HRMECs) induced by HG and hypoxia (HGY) to inhibit the formation of new blood vessels. In molecular studies, norkurarinone and isoxanthohumol could increase the mRNA levels of PI3K, AKT, and mTOR and inhibit autophagy, thereby leading to the downregulation of Beclin-1, LC3-Ⅱ, and ATG5 expression and the upregulation of P62 expression; this reduces the production of ROS and protects the mitochondrial structure. In summary, norkurarinone and isoxanthohumol inhibit autophagy by activating the PI3K/AKT/mTOR signaling pathway to prevent the formation of new blood vessels (Zhao et al., 2022).

##### 4.2.1.2 Autophagy of the BRB regulated by the AMPK pathway

Arjunolic acid (AA) is also a triterpenoid isolated from *C. paliurus* with antioxidant and antibacterial and anti-inflammatory effects. Like CPT, AA can exert therapeutic effects on diseases by activating autophagy through the AMPK/mTOR pathway (Zhang et al., 2019). Interestingly, AA can lower ROS by promoting the expression of HO-1 in DR rats and HG-induced ARPE cells, further affecting autophagy and protecting RPE cells (Zhang et al., 2022b)

Besides kidney injury, DIO can also be used to treat retinal injury (Zhong et al., 2022a; Zhong et al., 2023). After DIO intervention, the viability of ARPE-19 cells induced by HG is enhanced and the apoptosis of ARPE-19 cells is inhibited. The activation of AMPK can not only inhibit mTOR but also increase Nr2 nuclear translocation, thereby increasing the expression of downstream HO-1. When AMPK inhibitor is added, the above therapeutic effects of DIO are reversed. Therefore, the mechanism of action of DIO is similar to that of AA, which is to promote the increase of autophagy and reduce oxidative stress by activating the AMPK/Nrf2/HO-1 pathway to protect PRE cells (Hao and Gao, 2022).

##### 4.2.1.3 Autophagy of the BRB regulated by the SIRT1 pathway

In addition to DKD, RSV influences DR through autophagy regulation. It reduces apoptosis, ROS, and caspase-3 expression in HG-induced bovine retinal capillary endothelial cells by activating AMPK/Sirt1/PGC-1α signaling, enhancing autophagy, and reducing ROS-induced apoptosis (Li J. et al., 2017).

Artesunate is derived from artemisinin, and it boosts autophagy via the AMPK/SIRT1 pathway; lowers IL-6, MCP-1, and ROS levels; reduces BRB permeability; and inhibits retinal thickness increase in DR rats (Li et al., 2023).

Stachydrine is an alkaloid extracted from *Leonurus heterophyllus*. In DR rats and HG-treated HRMECs, stachydrine significantly increases p-AMPK, SIRT1, Beclin-1, and LC3-II protein levels, decreases levels of p62, and inhibits ROS and inflammatory factors. When SIRT1 inhibitors are applied, the ability of stachydrine to repair and protect the retinal fiber layer and protect HRMECs is limited. Therefore, stachydrine exerts therapeutic effects by activating autophagy and reducing inflammation through the AMPK/SIRT1 pathway (Yu et al., 2023).

Arbutin, a polyphenol extracted from arbutus leaves, has the effects of sterilization and anti-inflammation. Arbutin has been described above to protect ARPE cells by activating autophagy and inhibiting inflammation via the SIRT1 pathway (Ma et al., 2021).

Mel inhibits the Wnt/β-catenin pathway, reduces IL-1β and TNF-α expression, and restores Beclin-1 and LC3-II/I levels, protecting HRMECs from HG-induced damage by reducing inflammation and partially restoring autophagy dysfunction (Dehdashtian et al., 2018).

##### 4.2.1.4 Inhibition of autophagy to protect blood-retinal barrier

Quercetin, a flavonol compound present in various plants such as apples and potatoes, inhibits angiogenesis in HRMECs induced by HG. Its effects involve reduced LC3, Beclin-1, NLRP3, and IL-1 expression, leading to inhibition of autophagy and inflammation, potentially contributing to inhibition of RPE cell angiogenesis (Li R. et al., 2021).

Procyanidin is a polyphenol compound widely found in plants. It has strong inoxidizability and is mostly used to promote blood circulation and protect vision. Proanthocyanidins protect RPE cells by inhibiting autophagy through the p53/mTOR pathway (Li et al., 2022).

#### 4.2.2 Natural products interfere with glial cell autophagy to alleviate retinal damage

Epigallocatechin-3-gallate (EGCG), the major polyphenol in green tea, has the effects of anti-oxidation, anti-angiogenesis, and anti-thrombosis. In HG-induced Müller cells, apoptosis appears increased, and there is an increase in caspase-3 expression, a decrease in Beclin-1 and LC3-II expression and an increase in p62 expression. This change may be caused by lysosomal dysfunction. EGCG can regulate the formation of autophagosomes and autophagic lysosomes through the mTOR pathway, increasing autophagic flux and reversing the above situation. After the addition of 3-MA or rapamycin, the effect of EGCG is inhibited, which further proves the above-stated conclusion (Wang L. et al., 2019).

Gypenoside XVII (Gyp-17), an extract from the dried above-ground parts of the plant *Gynostemma gibbosum*, has hypoglycemic, antioxidant, and antitumor effects. After Gyp-17 treatment, the apoptosis of Müller cells is reduced and the damaged retinal structure is improved. In a study of the underlying mechanism, it was found that the contents of Beclin-1, Bcl-2, Atg5, and LC3-II in Müller cells decreased the expression of Bax, Caspase-3, and P62. Meanwhile, Gyp-17 exerts antioxidant effects, increasing the expression of superoxide dismutase (SOD) and glutathione peroxidase (GSH-px). Hence, Gyp-17 can play a protective role against Müller cells by enhancing autophagy and reducing oxidation (Luo et al., 2021).

Berberine inhibits HG-induced apoptosis in Müller cells, the mechanism of which is related to promoting autophagy through activation of the AMPK/mTOR pathway. In BBR-treated Müller cells, p-AMPK levels are increased and p-mTOR levels are decreased, along with increased expression of Beclin-1, LC3II, and Bcl-2 and decreased expression of Bax and caspase-3. After the addition of AMPK inhibitors, BBR could not exert a therapeutic effect on Müller cells (Chen H. et al., 2018).

#### 4.2.3 Natural products interfere with mitophagy to alleviate retinal damage

Upregulation of the PINK1/Parkin pathway by NGR1 enhances mitochondrial autophagy, resulting in an increase in the LC3-II/I ratio and a decrease in p62, ROS, and VEGF expression. This ultimately protects db/db mice and Müller cells from HG-induced damage. When PINK1 is knocked out, NGR1 is unable to play a normal role in cellular protection (Zhou P. et al., 2019).

Plumbagin is a naphthoquinone obtained from *Plumbago zeylanica* with antitumor and antiproliferative activities. As a complement to traditional vertebrates, *D. melanogaster* has the advantage of being easy to culture and reproduce. Recent studies have found that genes of human diseases such as diabetes and autism can be found in *Drosophila*. Plumbagin can reduce apoptosis to improve the visual decline and repair the eye structure of *D. melanogaster* with HG. Molecular studies have shown that intervention with plumbagin causes decreased expression of caspase-3 and p62 and increased expression of LC3, along with activation of autophagy and accumulation of a large number of autophagic vesicles. Plumbagin can also repair mitochondrial damage and relieve ER stress. The downregulation of ATF4 can reduce mitochondrial-ER stress and folded protein, inhibit redox imbalance, and decrease apoptosis. It has been proven that plumbagin can increase mitochondrial activity and decrease ATF4 level. At the same time, the levels of ROS and Nrf2 can be reduced to further alleviate the cell damage caused by oxidative stress (Catalani et al., 2023).

### 4.3 Natural products and DCM

**FIGURE 6:**
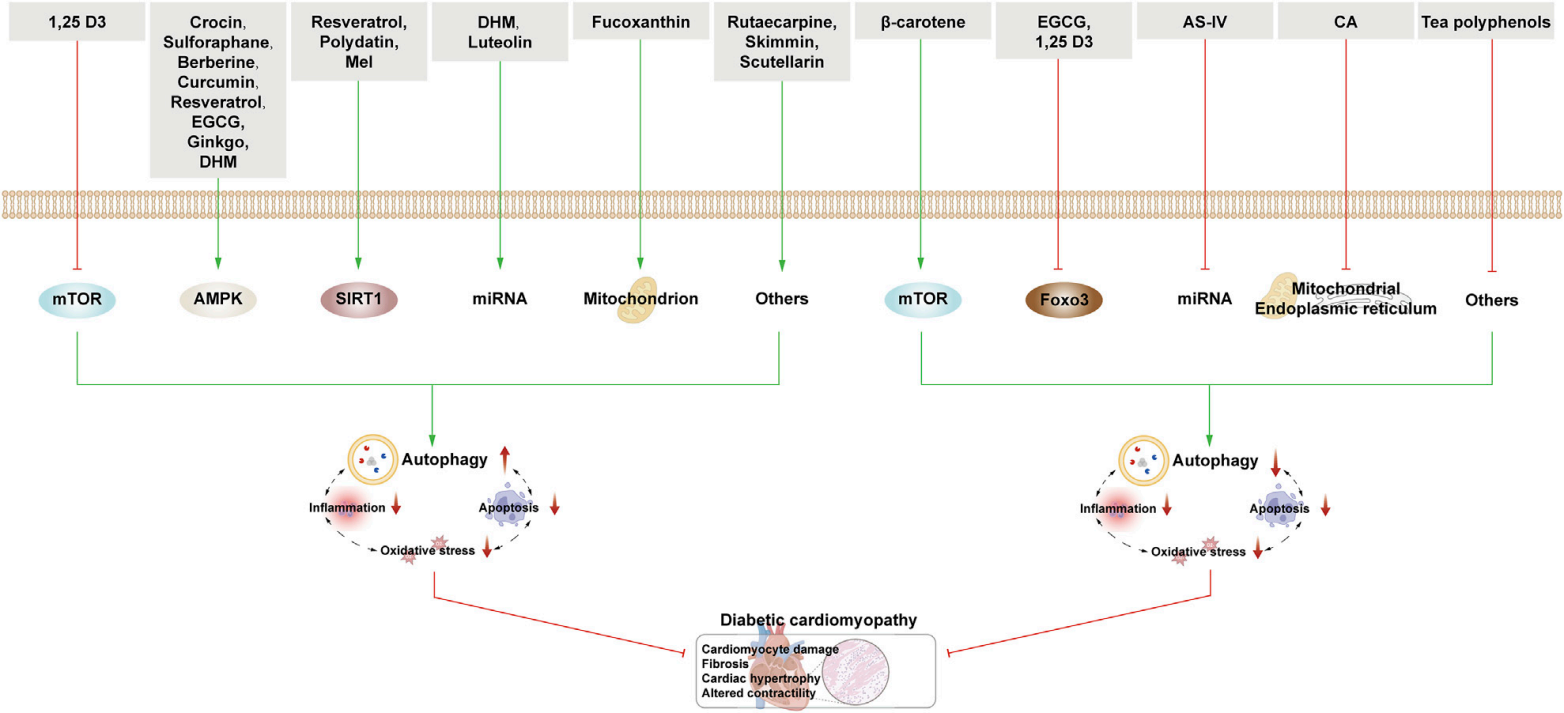
NPs target autophagy to fight DCM. Green arrows indicate downstream cellular events; red lines indicate inhibition. In DCM, the activation of autophagy has a dual effect on cardiomyocytes. NPs activate autophagy through various nutrient-sensing pathways, miRNAs, and other mechanisms. Interestingly, related NPs also inhibit autophagy via mTOR, FOXO3, miRNA, and other pathways.

This section examines the targeting of cardiomyocyte autophagy by NPs to combat DCM. The regulation of autophagy is bidirectional and influenced by nutrient sensing, oxidative stress, ER stress, inflammation, and apoptosis. There is also an interplay between autophagy and the aforementioned processes (Figure 6; Table 3).

**TABLE 3:** NPs to target autophagy as a means of combating DCM.

Name	Sources	*In vitro*/*In vivo*	Model	Dose and duration	Correlated target	Mechanisms	Activation/Inhibition of autophagy	Reference
Scutellarin	Skullcap	*In vivo*	Male SD rats	100,200 mg/kg	Beclin1, LC3-II	↑Beclin1,LC3-II ↓Bax,caspase-3	Activation	Su et al. (2022b)
				8 weeks				
Tea polyphenols	Tea	*In vivo*	Male SD rats	400 mg/kg	Beclin1, LC3-II	↓Beclin-1,LC3II/I ↑p62/SQSTM1	Inhibition	Zhou et al. (2018)
				8 weeks				
1,25-Dihydroxyvitamin-D3	vitamin D	*In vitro*	H9c2 cells	25,50,100, 200,400 nM 24h	β-catenin/TCF4/GSK-3β/mTOR	↓β-catenin/TCF4 ↓GSK-3β/mTOR	Activation	Wei et al. (2017a)
		*In vivo*	Male SD rats	150 ng/kg 8 weeks		↑Beclin-1,LC3II/I		
						↓p62/SQSTM1		
β-carotene	carrot	*In vitro*	H9c2 cells	40 μM 24h	PI3K/AKT/mTOR	↑PI3K/AKT/↓mTOR ↓LC3II ↓ ROS	Inhibition	Zhao et al. (2020)
						↓Bax/Bcl-2		
Crocin	Crocus sativus	*In vitro*	Cardiac myocytes	1.10 μM 3h	Hsp70/AMPK	↑Hsp70/AMPK	Activation	Feidantsis et al. (2018)
		*In vivo*	Male Wistar rats	10,20 mg/kg		↑Beclin1, LC3II ↓p62/SQSTM1 ↓Bax/Bcl-2 ↓caspase-3		
				2 weeks				
Sulforaphane	Broccoli	*In vivo*	C57BL/6J mice	0.5 mg/kg 4 months	LKB1/AMPK	↑Nrf2/HO-1 ↓4-HNE-LKB1 ↑LKB1, AMPK ↑SIRT1,LC3-Ⅱ	Activation	Zhang et al. (2014)
Berberine	Coptis chinensis	*In vitro*	H9c2 cells	100 nM 24h	AMPK/mTOR, GSK-3β, PGC-1α	↑AMPK ↓mTOR,GSK-3β ↑PGC-1α ↑Beclin-1, LC3 ↓Drp1 ↑Mfn1	Activation	Hang et al. (2018)
Curcumin	Turmeric	*In vitro*	H9c2 cells	10 μM 24h	AMPK/JNK1/mTOR	↑AMPK/JNK1 ↓mTOR	Activation	Yao et al. (2018)
		*In vivo*	C57BL/6J mice	200 mg/kg 3 months		↓Bcl-2/Beclin-1		
						↑Beclin-1, LC3II/I		
Resveratrol	Grape leaves	*In vitro*	H9c2 cells	25 μM 24–36h	AMPK/JNK1/mTOR	↑AMPK/JNK1 ↓mTOR	Activation	Xu et al. (2018)
						↓p70S6K1,4EBP1		
						↓Bcl-2/Beclin-1		
						↑Beclin-1, LC3II/I		
Resveratrol	Grape leaves	*In vitro*	H9c2 cells	10,25 μM 24h	AMPK/mTOR	↑AMPK/mTOR ↑Beclin-1, LC3 ↓Cx43	Activation	Wang et al. (2017a)
Epigallocatechin-3- gallate	Green tea	*In vivo*	Male SD rats	40,80 mg/kg 8 weeks	AMPK/mTOR, TGF-β/MMPs	↑AMPK/mTOR ↓TGF-β/MMPs ↑Beclin-1, LC3 ↓ECM	Activation	Jia et al. (2022)
Ginkgo biloba extract	Ginkgo biloba	*In vivo*	Male SD rats	100,200 mg/kg 15 weeks	AMPK/mTOR	↑AMPK/mTOR ↑Beclin-1,LC3II/I ↓p62	Activation	Yang et al. (2023)
Resveratrol	Grape leaves	*In vitro*	H9c2 cells	25 μM 12h	SIRT1/FOXO1/Rab7	↑SIRT1/Rab7 ↓Ac-FOXO1	Activation	Wang et al. (2014)
		*In vivo*	C57BL/6J mice	60,300 mg/kg 12 weeks		↑LC3II/I ↓p62		
						↓caspase-3		
Polydatin	Polygonum cuspidatum	*In vitro*	Neonatal mouse ventricular cardiomy-ocytes	10 μM 36h	SIRT3	↑SIRT3 ↑Beclin1,LC3Ⅱ ↑Atg5 ↓p62 ↑ATP, CS	Activation	Zhang et al. (2017b)
		*In vivo*	Constructed the Sirt3 knockout mice	7.5 mg/kg 4 weeks				
Melatonin	Pineal body	*In vitro*	Neonatal mouse ventricular cardiomy-ocytes	100 μM 12h	Mst1/Sirt3	↓p-Mst1/Mst1 ↑Sirt3 ↑p-AMPK/AMPK ↑p-ULK1/ULK1 ↑Beclin1,LC3Ⅱ ↑Atg5 ↓p62 ↑ATP, CS ↓ROS	Activation	Zhang et al. (2017a)
		*In vivo*	C57BL/6J mice	20 mg/kg 4 weeks				
Trehalose	Yeast	*In vivo*	Adult Akt2 knockout mice	1 mg/g 2 mouths	p38 MAPK/FOXO1	↑p38 MAPK	Activation	Wang and Ren (2016)
						↓Ac-FOXO1 ↑Beclin-1,LC3II/I ↑Atg5		
Epigallocatechin-3- gallate	Green tea	*In vivo*	Diabetic GK rats	100 mg/kg 12 weeks	FOXOS	↓FOXOs ↓Atg5,7,12 ↓LC3II/I ↓ ROS ↓Drp1	Inhibition	Liu et al. (2014b)
Epigallocatechin-3- gallate	Green tea	*In vitro*	H9c2 cells	20 μM 24h	Cytoplasmic acetylation of FoxO1	↓FOXO1 ↓Atg5,7,12 ↓LC3II/I ↓ ROS	Inhibition	Liu et al. (2014a)
1,25 D3	Vitamin D	*In vitro*	H9c2 cells	0.1,1.20 μM	1,25D3/VDR	↑1,25D3/VDR ↓FOXO1nuclear translocation ↓Beclin-1,LC3II/I ↓Bax/Bcl-2	Inhibition	Guo et al. (2020b)
				24h				
		*In vivo*	Zucker diabetic fatty rats	320U/kg 7weeks				
AS-IV	Astragalus	*In vitro*	H9c2 cells	12.5,25,50, 100 μM	miR-34a/Bcl2, pAKT/Bcl2	↓miR-34a ↑Bcl-2/Beclin-1	Inhibition	Zhu et al. (2019)
				48h		↓Beclin-1 ↓AKT/mTOR		
						↓LC3II/I ↑p62		
DHM	vine tea	*In vivo*	Male Wistar rats	100 mg/kg 2 weeks	miR-34a	↓miR-34a ↑Beclin-1,LC3II/I ↓Bax,caspase-3 ↑Bcl-2,p53	Activation	Ni et al. (2020)
DHM	vine tea	*In vivo*	Male C57BL/6 mice	100 mg/kg	AMPK/ULK1	↑AMPK/ULK1	Activation	Wu et al. (2017)
				14 weeks		↑Beclin-1,LC3II/I		
						↑SOD,GSH-Px		
						↓TNF-α, IL-6		
						↓Bax ↑Bcl-2		
Luteolin	Honeysuckle chrysanthemums	*In vivo*	Male SD rats	50,100,400 mg/kg 4 weeks	JNK/c-Jun, miR-221	↓p-JNK,p-c-Jun ↓miR-221 ↑LC3II/I ↓p62	Activation	Xiao et al. (2023)
Chlorogenic acid	Honeysuckle	*In vitro*	H9c2 cells	10,30 μM 48h	PERK, IRE1α, ATF6α Sec62, RTN3	↓PERK,IRE1α ↓ATF6α ↓GRP78 ↓CHOP ↓Sec62,RTN3	Inhibition	Preetha Rani et al. (2022)
		*In vivo*	Male Wistar rats	5.10 mg/kg 2mouths				
Fucoxanthin	Macroalgae	*In vitro*	H9c2 cells	1 μM 48h	Bnip3/Nix	↑Bnip3/Nix ↑Nrf2 ↓MMP ↓ROS ↑SOD1	Activation	Zheng et al. (2022)
		*In vivo*	Male SD rats	200 mg/kg 12 weeks	Nrf2			
Rutaecarpine	Evodia rutaecarpa	*In vitro*	H9c2 cells	0.1,1,10 μM 1h	TRPV1	↑TRPV1 ↑Beclin-1,LC3II/I ↓p62 ↓ROS ↓Bax,caspase-3 ↑Bcl-2	Activation	Song et al. (2023)
Skimmin	Skimmia	*In vitro*	Primary neonatal cardio- myocyte	2.10 μM	Beclin1, LC3-II/ROS/NLRP3	↑Beclin-1,LC3II/I ↓p62 ↓ROS ↓IL-1β,TNF-α ↓caspase-1	Activation	Liang et al. (2021)
				48h				
		*In vivo*	Male SD rats	15,30 mg/kg 16 weeks				

In DCM rats, scutellarin enhances autophagy to upregulate the expression of Beclin-1 and LC3-II and downregulate the expression of caspase-3, caspase-12, Bax, and Cyt-C to alleviate the symptoms of DCM (Su Y. et al., 2022).

Tea polyphenols represent various phenolic compounds found in tea, exhibiting antioxidant and hypoglycemic effects. Post-treatment, DCM rats manifest reduced blood glucose and lipids, improved cardiomyocyte arrangement, and enhanced cardiac function. Notably, levels of Beclin-1 and LC3-II/I are decreased, while p62/SQSTM1 levels increase, suggesting that tea polyphenols potentially inhibit autophagy, exerting therapeutic effects on DCM (Zhou et al., 2018). Catechins, such as EGCG, are the most important tea polyphenols and can regulate autophagy in a variety of ways to protect cardiac cells.

The above two kinds of NPs reflect that the activation or inhibition of autophagy can be effective in the treatment of DCM, and the reason for this may be related to the severity of cardiac tissue lesions. The subsequent section delineates the diverse mechanisms through which autophagy regulation by natural products aids in DCM treatment.

#### 4.3.1 Natural products mediate autophagy through the mTOR pathway to protect heart tissue

1,25-Dihydroxyvitamin-D3 (1,25 D3), the active form of vitamin D, acts in conjunction with the vitamin D receptor (VDR) to mitigate cardiac fibrosis and enhance cardiac function. Mechanistically, 1,25 D3 inhibits the mTOR pathway by curtailing β-catenin/T-cell factor/lymphoid enhancer factor (TCF4)/GSK-3β, thereby boosting LC3B-II/I levels and Beclin-1 expression, ultimately fostering autophagy (Wei H. et al., 2017).

β-carotene (BC), a vitamin A precursor, belongs to the carotenoid family. In AGEs-induced H2c9 cells, BC can improve cardiomyocyte function and inhibit cardiomyocyte apoptosis. The mechanism involves BC-mediated inhibition of autophagy through activation of the PI3K/Akt/mTOR signaling pathway, thereby reducing ER stress and ROS production, downregulating LC3II/I and pro-apoptotic protein Bax expression, and increasing anti-apoptotic protein Bcl-2 expression (Zhao et al., 2020).

#### 4.3.2 Natural products mediate autophagy through the AMPK pathway to protect heart tissue

Crocin, a water-soluble carotenoid from *Crocus sativus*, is clinically employed for cardiovascular and central nervous system diseases. Crocin activates Heat Shock Protein 70 (Hsp70) in DCM rats, stimulating AMPK, which in turn increases Beclin-1 and LC3II/I levels, while reducing p62/SQSTM1, Bax/Bcl-2, and caspase-3 levels, thereby fortifying heart cell protection through enhanced autophagy (Feidantsis et al., 2018).

Sulforaphane, a sulfur-containing compound extracted from cruciferous plants, especially broccoli, is the most common antioxidant. It can inhibit cardiac lipid accumulation and ameliorate cardiac inflammation, oxidative stress, and fibrosis. As a strong activator of Nrf2, sulforaphane upregulates Nrf2, decreases 4-HNE expression and 4-HNE-LKB1 conjugates, increases LKB1 and subsequent AMPK activity, and upregulates SIRT1 and LC3-II expression. Thus, sulforaphane exerts its therapeutic effect by activating autophagy through the LKB1/AMPK pathway (Zhang et al., 2014).

BBR can activate AMPK to stimulate mitochondrial biogenesis and restore autophagic flux to alleviate HG-induced cardiac hypertrophy and restore myocardial function. By restoring autophagic flux through AMPK/mTOR, BBR enhances Beclin-1, LC3, and Atg5 levels. The process of mitochondrial fission is controlled by dynamic-related protein 1 (Drp1), and GSK-3β activation is positively correlated with Drp1 expression. AMPK activated by BBR inhibits GSK-3β, which in turn inhibits Drp1 from attenuating mitochondrial fission. AMPK can also increase the expression of PGC-1a (an important transcription factor responsible for mitogenesis) to stimulate mitosis, thereby generating new mitochondria. BBR also promotes mitophagy to remove damaged mitochondria and the expression of the mitochondria-related fusion protein Mitofusion 1 (Mfn1), which jointly regulates mitochondrial homeostasis and promotes the recovery of myocardial cells (Hang et al., 2018).

Curcumin can regulate autophagy through the mTOR pathway to protect podocytes, and can also activate autophagy through the JNK1/AMPK/mTOR pathway to protect cardiomyocytes and improve cardiomyocyte function. Curcumin activates JNK1 to induce Bcl-2 phosphorylation, destroys Bcl-2/Beclin1 structure, and releases a large amount of Beclin1 to promote autophagy. At the same time, the activation of AMPK pathway and inhibition of mTOR pathway in rat cardiomyocytes (H9c2) cells were also found in the study, which further activated autophagy (Yao et al., 2018).

The mechanism of JNK1-induced dissociation of Bcl-2 and Beclin-1 to promote autophagy recovery has also been observed in RSV-mediated mouse cardiomyocytes. In addition, RSV can also inhibit mTOR and its downstream effectors p70S6K1 and 4EBP1 through AMPK/JNK1 activation, restoring autophagy levels (Xu et al., 2018). Moreover, connexin 43 (Cx43) has been found to be the major connexin in ventricular cardiomyocytes, and its expression is upregulated under HG conditions; thus, it may be closely related to cardiac dysfunction in diabetic rats. RSV can increase autophagy flux and decrease the high expression of Cx43 induced by HG through AMPK/mTOR, which plays a role in DCM treatment (Wang GY. et al., 2017).

EGCG can play an anti-fibrosis role in the heart of DCM rats. Transforming growth factor-β (TGF-β)/matrix metalloproteins (MMPs) signaling pathway can regulate the excessive deposition of extracellular matrix (ECM) components, and then develop into myocardial fibrosis. EGCG has been proved to activate autophagy through the AMPK/mTOR pathway, increase the expression of Beclin-1 and LC3, and inhibit the TGF-β/MMPs pathway, thereby playing a role in anti-fibrosis and improvement of myocardial hypertrophy and injury (Jia et al., 2022).

*Ginkgo biloba* extract has been shown to regulate the AMPK/mTOR pathway, activate autophagy, and improve cardiac function by decreasing myocardial pathological changes (Yang et al., 2023).

#### 4.3.3 Natural products mediate autophagy through the SIRT family to protect heart tissue

RSV can regulate autophagy through the AMPK/mTOR pathway as described above. Here, the focus will be on the activation of autophagy by RSV through the SIRT1 pathway for the treatment of DCM. The upregulation of p62, downregulation of LC3II/I, increased expression of FOXO1 and caspase-3, and inhibition of SIRT1 activity can be observed in DCM mice and excessive oxidative stress- and HG-induced H9c2 cells. After adding RSV, the above trend is reversed, and SIRT1 deacetylates FOXO1. Rab7 is the key factor for autophagy maturation and its fusion with lysosomes. Deacetylated FOXO1 activates the expression of Rab7 and increases autophagy flux. Therefore, RSV regulates autophagy flux through the SIRT1/FOXO1/Rab7 axis to prevent DCM (Wang et al., 2014).

Mammalian STE20-like kinase 1 (Mst1) inhibits autophagy by suppressing the activity of the PI3K complex. SIRT3 activates autophagy in a pathway similar to that of SIRT1. However, SIRT3 acts downstream of Mst1, upregulating both autophagy and filamentous autophagy only when Mst1 inhibition is present.

Polydatin, a natural extract of *Silybum marianum*, enhances mitochondrial function and cardiomyocyte protection by activating autophagy through SIRT3. Its effectiveness diminishes in SIRT3-deficient mice, emphasizing SIRT3’s role in activating autophagy, improving mitochondrial bioenergetics, and protecting damaged cardiomyocytes (Zhang et al., 2017b).

Mel activates autophagy and restores mitochondrial function by activating the Mst1/Sirt3 pathway or the Parkin translocation, so as to alleviate cardiac insufficiency. The treatment with melatonin resulted in a reduction of mitochondrial damage and apoptosis. This was demonstrated by a decrease in ROS and caspase-3, as well as an increase in ATP and Bcl-2. The underlying mechanism is that after Mel intervention, the p-Mst1/Mst1 ratio is decreased and Sirt3, the ratio of p-AMPK/AMPK to p-ULK1/ULK1 ratio, Beclin-1, Atg5, and LC3-II expression are elevated, and p62 expression is decreased, indicating that autophagy is enhanced. However, Mel could not exert its therapeutic effect when Mst1 was knocked out (Zhang et al., 2017a). According to Wang et al., Mel promotes mitophagy and maintains mitochondrial homeostasis for the treatment of DCM in DCM mice. This is likely because Mel inhibits the phosphorylation of Mst1, promotes the translocation of Parkin to the mitochondria, and increases the expression of PINK1 (Wang S. et al., 2018). Moreover, Mel’s activation of the SIRT1/Nrf2 pathway mitigates As-induced cardiomyocyte apoptosis (Yarmohammadi et al., 2023). Recently, it has also been shown that there is a close relationship between Mel and insulin secretion, so it may be of great significance to treat DCM in the future through the autophagy regulation mechanism of Mel (Dewanjee et al., 2021).

#### 4.3.4 Natural products mediate autophagy through the FOXO family to protect heart tissue

Trehalose, a stable natural disaccharide prevalent in various organisms, functions as an independent autophagy inducer of mTOR. It modulates p38 MAPK and Foxo1 dephosphorylation-related autophagy; upregulates Atg5, Beclin-1, and LC3II/I expression; and reduces p62 levels. This mitigates ER stress, rectifying myocardial contraction defects and apoptosis (Wang and Ren, 2016). A study conducted in 2020 suggested that alginate activates autophagy levels in T2DM mice and alleviates DCM (Choi et al., 2020).

EGCG mitigates autophagy levels through FOXO1, lowering ROS production and oxidative stress, and preserving mitochondrial homeostasis, thereby safeguarding HG-induced H9c2 cells. Liu et al., 2014b reported increased ROS levels, Drp1 expression, Atg7, Atg12-Atg5, and LC3-II/LC3-I ratio in Goto-Kakizaki (GK) rat cardiomyocytes. Further study found that the expression of FOXOs was increased, but the mTOR signal was not expressed, suggesting that FOXOs may regulate the enhancement of cardiac autophagy in GK rats. The addition of EGCG decreased the expression of FOXOs, reversed the above trend, and rescued myocardial cells. Subsequently, they proposed that cytopathic acetylation of FOXO1 in the cytoplasm is the key to mediating autophagy, and EGCG can reduce acetylated foxo1, thus reducing autophagy (Liu et al., 2014a).

1,25 D3, in tandem with VDR, hinders the nuclear translocation of FOXO1, negatively impacting FOXO1 gene transcription to inhibit autophagy activity, which protects cardiac tissues. In Zucker diabetic fatty rats, the ratio of LC3II/I to Bax/Bcl-2 and the levels of Beclin-1 and caspase-3 are upregulated. These effects are inhibited after adding 1,25 D3 or FOXO1 transcriptional activity inhibitors (Guo X. et al., 2020).

#### 4.3.5 Natural products mediate autophagy through other means to protect heart tissue

The study found that the pro-aging miRNA, miR-34a, is overactivated in the hearts of diabetic patients and mice, leading to DCM. Under the premise of HG induction and overexpression of miR-34a, LC3II/I increases and p62 decreases in mouse cardiomyocytes. Overexpression of miR-34a inhibits Bcl-2 expression, reducing Bcl-2’s binding to Beclin-1, elevating Beclin-1 expression. At the same time, the expression of AKT is inhibited, and inhibition of PI3K/AKT in turn inhibits the downstream mTOR pathway, further promoting autophagy. However, after the addition of AS-IV, the above process is reversed and the expression of miR-34 is inhibited, indicating that AS-IV inhibits autophagy and oxidative stress through miR-34a/Bcl-2 and pAKT/Bcl2-2 pathways, thus protecting myocardial cells (Zhu et al., 2019).

Dihydromyricetin (DHM) ameliorates DCM by reducing miR-34a expression, promoting autophagy, maintaining mitochondrial homeostasis, and diminishing myocardial cell apoptosis (Ni et al., 2020). Wu et al. suggested that DHM activates AMPK, promotes autophagy, improves mitochondrial function, reduces oxidative stress and inflammation in HG-induced DCM mice, thereby stabilizing myocardial cell function (Wu et al., 2017). Research on the effects of AS-IV and DHM on miR-34a usedmiR-34a inhibitors and miR-34a agonists to confirm the authors’ views. However, the results were not comprehensive in revealing the specific mechanism of miR-34a regulating autophagy. Considering the expression analysis of miR-34a in the diabetic heart, it may be a promising target for the treatment of DCM. Extensive research is needed to further reveal the mechanism of miR-34a in the treatment of DCM.

Luteolin is a flavonoid compound widely found in honeysuckles and wild chrysanthemums. Luteolin inhibits p-JNK and p-c-Jun in DCM rats thereby, suppressing miR-221 expression. Studies confirm miR-221’s autophagy-inhibiting role, signifying luteolin’s autophagy promotion via JNK/c-Jun and miR-221 pathways, improving diabetic myocardial structure and reducing myocardial fibrosis (Xiao et al., 2023).

Chlorogenic acid (CA), mainly from honeysuckle, has anti-inflammatory, antimicrobial, and antioxidant effects. CA has been shown to protect cardiomyocytes by reducing ER stress and ER-mediated autophagy. High expression of ER stress marker GRP78 is found in HG-induced cardiomyocytes. The addition of CA reduced the expression of GRP78, CHOP, and caspase-12, as well as the expression of Sec62 and RTN3, which stimulates ER autophagy. The mechanism is related to the reduction of ER stress-mediated autophagy by CA-regulated IRE1α, ATF6α, and PERK/eIF2α (Preetha Rani et al., 2022). Besides CA, Astragalus polysaccharides, ginsenoside Rg1, and matrine also mitigate DCM by inhibiting ER stress (Yu et al., 2016; Hou et al., 2019; Sun et al., 2019).

Fucoxanthin is a carotenoid compound that widely exists in algae, especially macroalgae, and it has anti-inflammatory and antioxidant effects. Under the induction of HG, the mitochondrial membrane potential (MMP) of H9c2 cells decreases and the mitochondrial morphology changes, resulting in mitochondrial damage and myocardial hypertrophy. Fucoxanthin can reverse these processes. Fucoxanthin regulates the aggregation of Bnip3 or Nix to mitochondria, which, as mitochondrial outer membrane proteins, can bind with LC3 to regulate mitophagy, improve the degradation of damaged mitochondria, and maintain mitochondrial homeostasis. In addition, Fucoxanthin promotes the nuclear translocation of Nrf2, further reduces ROS and lowers the level of oxidative stress. Taken together, fucoxanthin promotes mitophagy through Bnip3/Nix and upregulates Nrf2 to reduce oxidative stress, thus protecting HG-induced cardiomyocytes (Zheng et al., 2022).

Rutaecarpine, an alkaloid from *Evodia rutaecarpa*, is clinically used for the treatment of tardive dyskinesia and hypertension. Through TRPV1-mediated autophagy activation, rutaecarpine increases Beclin-1 and LC3-II/I levels and decreases p62 expression. It also reduces the expression of oxidative stress-related proteins, decreases ROS to upregulate Bcl-2, and downregulates Bax and caspase-3 to inhibit HG-induced apoptosis of cardiomyocytes, to improve cell viability (Song et al., 2023).

Skimmin, a natural coumarin compound derived from many plants and citrus fruits, has good anti-inflammatory and immunomodulatory activities. Skimmin treatment results in increased Beclin-1 and LC3-II/I and decreased p62; furthermore, it activates autophagy and eliminates mitochondria to reduce ROS content and inhibits NLRP3 inflammatory corpuscles, thus decreasing TNFα, IL-6, IL-1β, and caspase-1. Therefore, the protective mechanism of skimmin on myocardium may be related to autophagy-ROS-NLRP3 inflammatory corpuscle pathway in the heart tissue (Liang et al., 2021).

### 4.4 Natural products and DPN

**FIGURE 7:**
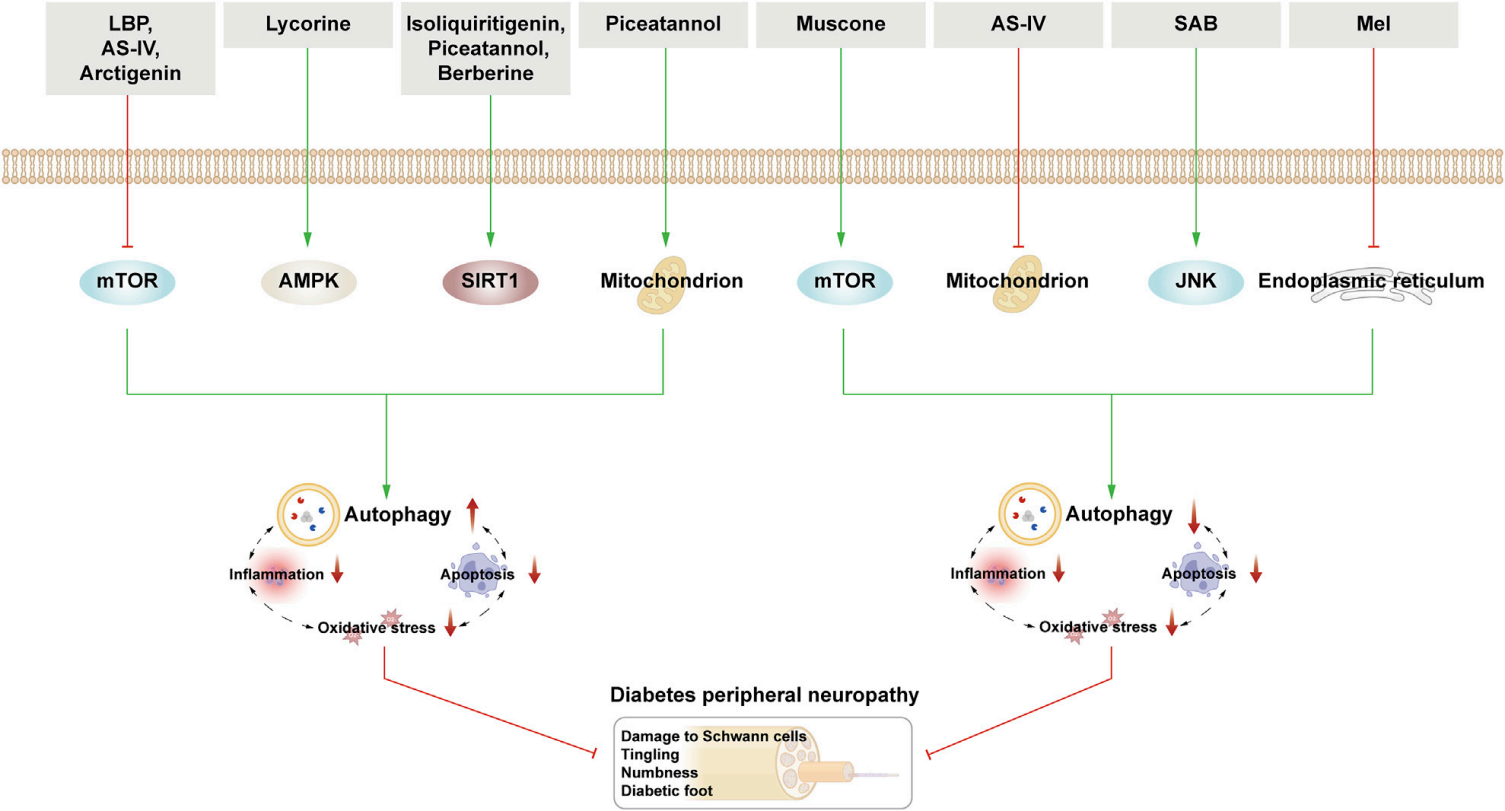
NPs target autophagy to fight DPN. Green arrows indicate downstream cellular events; red lines indicate inhibition. In DPN, the activation of autophagy has a dual effect on cardiomyocytes. NPs activate autophagy through various nutrient-sensing pathways. Related NPs also inhibit autophagy via mTOR, JNK, and other ER stress.

This section examines the targeting of cardiomyocyte autophagy by NPs to treat DPN. The regulation of autophagy is bidirectional and influenced by nutrient sensing, oxidative stress, ER stress, inflammation, and apoptosis. There is also an interplay between autophagy and the aforementioned processes (Figure 7; Table 4).

**TABLE 4:** NPs to target autophagy as a means of combating DPN.

Name	Sources	*In vitro*/*In vivo*	Model	Dose and duration	Correlated target	Mechanisms	Activation/Inhibition of autophagy	Reference
Quercetin	Apple	*In vitro*	Schwanncells	25 μM 24h	Beclin1, LC3	↑Beclin-1,LC3	Activation	Qu et al. (2014)
		*In vitro*	RSC96 cells	25 μM 24h				
LBP	Lycium barbarum	*In vivo*	Male SD rats	500 mg/kg 12 weeks	mTOR/p70S6K	↓mTOR/p70S6K ↑Beclin-1,LC3II/I ↓p62	Activation	Liu et al. (2018)
AS-IV	Astragalus	*In vitro*	RSC96 cells	0.5,10,15,20 μM 48h	miR-155, PI3K/AKT/mTOR	↑miR-155 ↓PI3K/AKT/mTOR ↑Beclin-1, LC3 ↓p62	Activation	Yin et al. (2021)
		*In vivo*	Adult GK rats	20,40,80 mg/kg				
				6 weeks				
Arctigenin	Arctium lappa	*In vivo*	Swiss albino male mice	25,50 mg/kg	AMPK/mTOR, PI3K/AKT/mTOR	↑AMPK ↓PI3K/AKT/mTOR ↑Beclin-1,LC3II/I ↓Bax,caspase-3 ↓GSH ↑Bcl-2,SOD	Activation	Medras et al. (2023)
				3 weeks				
Muscone	Musk	*In vitro*	RSC96 cells	0.1,1,10,50 μM 48h	AKT/mTOR	↑AKT/mTOR ↓Beclin-1, LC3II/I ↑P62 ↓Bax,caspase-3	Inhibition	Dong et al. (2019a)
Lycorine	Lycoris radiata Herb	*In vitro*	RSC96 cells	0.96,1.44, 2.87,5.74 μM 48h	AMPK/MMP9	↑MPK ↓MMP9 ↑Beclin-1, LC3-II	Activation	Yuan et al. (2022)
		*In vivo*	C57BL/6J mice	20 mg/kg 3 weeks		↓p62		
						↑Atg3		
Isoliquiriti- genin	Glycyrrhiza uralensis Fisch	*In vitro*	Mouse N2A cell	2.5.5 μM 6h	SIRT1/AMPK/mTOR	↑SIRT1/AMPK ↓mTOR ↑FOXO3, Nrf2 ↑Beclin-1,LC3-II ↑PGC-1α,TFAM	Activation	Yerra et al. (2017)
		*In vivo*	Male SD rats	10,20 mg/kg 2 weeks				
Berberine	Coptis chinensis	*In vitro*	Mouse N2A cell	5.10 μM 6h	SIRT1/AMPK/mTOR	↑SIRT1/AMPK ↓mTOR ↑FOXO3, Nrf2 ↑Beclin-1, LC3II ↑PGC-1α,TFAM	Activation	Yerra et al. (2018)
		*In vivo*	Male SD rats	50,100 mg/kg 2 weeks				
Piceatannol	Grape leaves	*In vitro*	Mouse N2A cell	5.10 μM 24h	SIRT1/PINK/Parkin	↑SIRT1 ↑PINK/Parkin ↑Nrf2, SOD2 ↑PGC-1α	Activation	Khan et al. (2023)
		*In vivo*	Male SD rats	10,20 mg/kg 2 weeks				
AS-IV	Astragalus	*In vitro*	RSC96 cells	50 μM 72h	PINK/Parkin	↓PINK/Parkin ↓LC3 ↓ROS	Inhibition	Wei et al. (2022)
Salvianolic acid B	Danshen	*In vitro*	RSC96 cells	0.1,1,10 μM	JNK	↓JNK ↓Beclin-1,LC3II/I ↑P62 ↑Bcl-2 ↓caspase-3	Inhibition	Wang et al. (2019b)
				72h				
Melatonin	Pineal body	*In vitro*	SH-SY 5Y cells	10 μM	miR-214-3p/caspase-1, miR-214-3p/ATG12	↑miR-214-3p ↓caspase-1 ↓Atg12 ↓Beclin-1,LC3-II ↓NLRP3,GSDMD	Inhibition	Che et al. (2020)
				24h				
		*In vivo*	Male KM mice	10 mg/kg 8 weeks				
Melatonin	Pineal body	*In vitro*	RT4-D6P2T rat Schwann cell	1,5,10 μM	PERK/eIF2α/ATF4/CHOP	↓PERK,eIF2-α ↓ATF4, ATF6 ↓CHOP ↓LC3	Inhibition	Salem et al. (2023)
				24h				

In 2014, Qu et al., 2014 showed that quercetin augmented Beclin-1 and LC3 expression and enhanced autophagy, subsequently protecting cell viability and structure of SCs induced by HG. Although this early experimental study delved into DPN treatment via autophagy modulation using NPs, Qu et al. did not extensively explore autophagy regulation mechanisms, possibly constrained by that period’s limitations. Subsequent research has verified that NP mechanisms govern autophagy regulation, aiming for safer and more effective DPN treatments.

#### 4.4.1 Natural products mediate autophagy through mTOR pathway to protect neurons

*Lycium barbarum* polysaccharides (LBP) alleviate DNP rat hyperalgesia and improve nerve myelination structural damage induced by HG. The mechanism may be related to inhibition of the mTOR/p70S6K pathway by LBP, thereby stimulating autophagy, increasing Beclin-1 and LC3-II levels, and reducing p62 expression (Liu et al., 2018).

AS-IV modulates autophagy to alleviate DKD and DCM. *In vivo* and *in vitro* DPN models showcase AS-IV promoting autophagy, attenuating peripheral nerve myelin damage, and enhancing DPN rat neurological function. AS-IV upregulates miR-155 expression, hindering the PI3K/Akt/mTOR pathway, elevating Beclin-1 and LC3 levels, fostering autophagy (Yin et al., 2021).

Arctigenin, extracted from *Arctium lappa*, exhibits anti-inflammatory and antiviral effects. Arctiin enhances AMPK expression, inhibits PI3K/AKT/mTOR, promotes autophagy, upregulates Beclin-1 and LC3II/I, and downregulates pro-apoptotic and oxidative stress factors, thus mitigating neuronal function and HG-induced structural damage (Medras et al., 2023).

Muscone, derived from musk, is often used to treat sciatica and angina pectoris. HG-induced Rat Schwann Cell (RSC96) interventions revealed increased AKT, mTOR, and p62 expressions, with decreased Beclin-1 and LC3II/I levels. Muscone inhibits autophagy via the AKT/mTOR pathway activation, safeguarding RSC 96 cell viability and reducing apoptosis (Dong J. et al., 2019).

#### 4.4.2 Natural products mediate autophagy through AMPK pathway to protect neurons

Lycorine from Lycoris radiata shows anti-inflammatory and analgesic properties. The expression of Beclin-1, LCE-II, and Atg3 was reduced in HG-cultured RSC 96 cells and DPN rats. The addition of lithopone alleviates the mitochondrial and ER dysfunction, while phosphorylating AMPK and inhibiting the expression of MMP9, thus reversing the above trend, promoting SCs autophagy and protecting SCs (Yuan et al., 2022).

#### 4.4.3 Natural products mediate autophagy through the SIRT1 pathway to protect neurons

Recent studies have shown that NPs mitigate neuronal damage and neuroinflammation in STZ-induced rats and HG-induced neuroblastoma (N2A) cells. The likely mechanism is that NPs promote the expression of FOXO3a and Nrf2 and its downstream enzyme antioxidant enzyme NQO1 through SIRT1 activation, which improves the antioxidant capacity and reduces the ROS concentration. Activation of the SIRT1 pathway also promotes mitochondrial transcription factor A (TFAM) expression and deacetylation of PGC-1α to protect mitochondrial biogenesis.

Isoliquiritigenin, a flavonoid from licorice, exhibits anti-inflammatory and antioxidant effects. Piceatannol, a polyphenolic compound, has similar effects to its precursor—RSV, and can thus act as a strong activator of SIRT1 to affect autophagy. Isoglycyrrhizin, piceatannol, and BBR can all activate SIRT1 to regulate the above mechanisms for the treatment of DPN. SIRT1 activation by isoglycyrrhizin and BBR upregulates AMPK, inhibits mTOR, and activates autophagy (Yerra et al., 2017; Yerra et al., 2018). Piceatannol-induced SIRT1 activation elevates PINK1 and PARKIN, activating mitochondrial autophagy, further safeguarding SCs and mitochondrial biogenesis (Khan et al., 2023).

#### 4.4.4 Natural products protect neurons by inhibiting autophagy

AS-IV protects neuronal cells by activating autophagy through inhibition of the PI3K/Akt/mTOR pathway. Here, AS-IV alleviates oxidative stress and inhibits excessive mitophagy to protect SCs. HG-induced AS-IV in SCs can inhibit ROS production, downregulate the expression of LC3, PINK, and Parkin, and improve mitochondrial structure and function, exerting a therapeutic effect on DPN (Wei et al., 2022).

Salvianolic acid B, derived from *Salvia divinorum*, has anti-inflammatory, antifibrotic, and antitumor effects. Salvianolic acid B decreases the expression of P-JNK, caspase-3, caspase-9, Beclin-1, and the ratio of LC3II/I, and increases the expression of p62 and Bcl-2 in HG-induced RSC96 cells, confirming that salvianolic acid B can inhibit HG-induced autophagy and apoptosis by inhibiting JNK to protect SCs (Wang QQ. et al., 2019).

It has been shown that Mel inhibits autophagy and neuronal pyroptosis by regulating the miR-214-3p/caspase-1 and miR-214-3p/ATG12 axes, to play a role in neuronal protection. NLRP3 inflammasomes can activate caspase-1 to cleave the N-segment domain of gasdermin D, which is a key step in inducing pyroptosis. The downregulation of NLRP3, caspase-1, and GSDMD-n has been observed after Mel treatment of HG-induced nerve cells. This is likely because Mel upregulates the level of miR-214-3p and downregulates the level of caspase-1, and miR-214-3p can, in turn, target and regulate caspase-1; hence, Mel can inhibit neuronal pyroptosis. MiR-214-3p can also bind to Atg12, inhibit the expression of Atg12, and reduce the levels of LC3-II and Beclin-1, thus inhibiting autophagy. Therefore, Mel can regulate autophagy through miR-214-3p to treat DPN (Che et al., 2020).

Experiments have also shown that the expression of ATF4, ATF6, PERK, eIF2-α, CHOP, and LC3 in HG-induced SCs is decreased after Mel treatment. Thus, Mel enhances cell viability by inhibiting ER-mediated autophagy through the PERK-eIF2α-ATF4-CHOP pathway (Salem et al., 2023).

## 5 Novel drug delivery systems—nanotechnology

NPs, despite their therapeutic potential, often face limitations such as poor bioavailability. Conventional administration routes, susceptibility to first-pass effects, and enzymatic degradation in the gastrointestinal tract all reduce their efficacy (Yuan et al., 2023). Therefore, it is essential to continually enhance the drug delivery system and improve the targeting ability of drugs. Nanotechnology in medicine precisely targets the action sites and improves the efficacy of drug delivery. Nanocarriers like polymers, nanoparticles, nanoemulsions, and nanomicelles refine drug delivery, enhance drug targeting, release control, and extend the metabolic life of the drug for maximal therapeutic effect (Hao et al., 2020). Encapsulating scutellarin in nanoparticles augments its bioavailability, positively affecting STZ-induced rat retinal structure as compared to free scutellarin. Furthermore, free scutellarin is also bitter, which can reduce patient compliance, but nanoparticles can mask this unpleasant taste (Wang J. et al., 2017). Compared to free quercetin, quercetin nanoparticle complexes, prolong drug circulation and release time, hence alleviating DKD rat proteinuria and hyperglycemia symptoms (Tong et al., 2017). Clinical trials have found that nano-curcumin significantly enhances curcumin bioavailability, reduces glycated hemoglobin and fasting blood glucose levels (Asadi et al., 2019). The application of nanotechnology has been widely demonstrated to improve the efficacy of targeted therapies for NPs, reduce side effects, and improve patient compliance. This could be very useful to improve the efficacy of NPs for DMC through autophagy.

## 6 Limitations

With continued advancements in modern research, autophagy is understood to play an important role in the occurrence of DMC, but the specific regulatory role of NPs in the occurrence and development of DMC through autophagy has not yet been fully clarified. Specifically, potential biomarkers related to autophagy, coordination between autophagy-related regulatory factors, and the specific state of autophagy *in vivo* have not been clearly elucidated. Although this review objectively discusses the studies related to the treatment of DMC by NPs through autophagy, the literature selection was inevitably subjective or incomplete. In addition, most of the existing animal and cell experimental studies are based on the lack of some strong clinical research evidence. There are also shortcomings in traditional drug delivery routes, which limit the direct application of NPs in clinical practice. Future studies should focus on exploring the related mechanisms of NPs regulation of autophagy in DMC patients and develop more accurate biomarkers of autophagy mechanism and technical tools to detect autophagy status. We should actively explore new drug delivery routes, consider nanotechnology as the starting point, develop new autophagy regulatory drugs, and increase clinical research to better apply them to DMC patients.

## 7 Conclusion and prospects

DMC is a prevalent metabolic disease characterized by hyperglycemia. Autophagy—a crucial metabolic pathway governing bodily energy balance—holds substantial relevance to DMC. Recent investigations underscore the potential of various NPs in restoring impaired autophagy to treat DMC. The relationship between autophagy and DMC is intricate, entailing a complex interplay with nutrient perception, oxidative stress, ER stress, inflammation, and apoptosis, often being interconnected among themselves. NPs exhibiting multitarget properties hold promise in modulating diverse pathways, affecting multiple cell types, and fostering beneficial effects like anti-inflammatory, antioxidant, and anti-apoptotic actions, while reinstating autophagy. Consequently, they offer unique advantages in managing DMC. For instance, RSV activates autophagy in podocytes, retinal capillary endothelial cells, and cardiomyocytes via the AMPK, SIRT1, and miRNA pathways, concurrently reducing oxidative stress and inflammation. Similarly, BBR activates autophagy and inhibits apoptosis in DMC cells through AMPK, SIRT1, and mitochondria-associated proteins.

It is noteworthy that autophagy exhibits a dual role in DMC progression. Its initiation or inhibition does not straightforwardly correlate with cellular protection or harm. While upregulation of autophagic flux often ameliorates DMC-related symptoms, there is a growing interest in inhibiting overactive autophagy *in vivo*. Melatonin regulates autophagy in DKD, DR, and DCM, but inhibits it in DPN. EGCG activates autophagy in Müller cells and cardiomyocytes, but inhibits it in cardiomyocytes via FOXO1. Astragaloside exhibits activation and inhibition of autophagy in the kidney and heart, respectively. In neurons, astragaloside activates autophagy through mTOR and inhibits mitophagy. This duality in autophagy’s effects may relate to disease type, disease stage, experimental model variability, or autophagy assessment methods. Unraveling autophagy’s precise role in different DMC stages and understanding its regulation depth remain critical for advancing disease treatment. While natural compounds present accessibility and safety advantages over conventional drugs, extensive long-term research is paramount.

This approach also has some drawbacks. Current experimental studies predominantly rely on *in vivo* or *in vitro* models, with limited clinical data available. Conducting more clinical studies to establish drug safety and efficacy is therefore essential. Moreover, overcoming traditional drug delivery constraints and improving pharmacokinetic and pharmacodynamic properties should be the focal points for future research.

## Figures and Tables

**Table:** 

**DMC**	Diabetic microangiopathy
**DKD**	Diabetic kidney disease
**DR**	Diabetic retinopathy
**DCM**	Diabetic cardiomyopathy
**DPN**	Diabetic peripheral neuropathy
**NPs**	Natural products
**MTs**	Microtubules
**CMA**	Chaperone-mediated autophagy
**mTOR**	mammalian target of rapamycin
**LC3**	Light chain3
**MAPK**	Mitogen activated protein kinase
**AMP**	Adenosine 5‘-monophosphate
**AMPK**	Adenosine 5‘-monophosphate-activated protein kinase
**TSC 1/2**	Tuberous Sclerosis 1/2
**ROS**	Reactive oxygen species
**ER**	Endoplasmic reticulum
**Atg**	Autophagy-related gene
**PE**	Phosphatidylethanolamine
**PI3K**	phosphatidylinositol -3- kinase
**PI3P**	phosphatidylinositol 3-phosphate
**SIRT1**	Silent Information Regulator 1
**JNK**	c-Jun N-terminal kinase
**Nrf2**	Nuclear factor erythroid 2 related factor 2
**Keap1**	Kelch-like ech-associated protein 1
**PINK1**	PTEN induced kinase 1
**LKB1**	Liver kinase B1
**ARE**	Antioxidant response element
**T2DM**	Type 2 diabetes mellitus
**IR**	insulin resistance
**UPRS**	Unfolded protein response
**ERAD**	Endoplasmic reticulateddegradation
**PERK**	PKR-like eukaryotic initiation factor 2αkinase
**IRE1α**	Inositol-requiring enzyme 1α
**ATF6α**	Activating transcription factor6α
**elF2α**	Eukaryotic translation initiation factor 2α
**mTORC1**	mTOR complex I
**TNF-α**	Tumor necrosis factor-α
**NF-κB**	Nuclear factor-κB
**AGEs**	Advanced glycation end products
**TFEB**	Transcription factor EB
**MOMP**	Mitochondrial outer membrane permeabilization
**Cyt c**	Cytochrome c
**AIF**	Apoptosis inducing factor
**3- MA**	3- methyladenine
**CQ**	Chloroquine
**GFR**	Glomerular filtration rate
**RAAS**	Renin-angiotensin-aldosterone-system
**STZ**	Streptozotocin
**miR-486**	MicroRNA-486
**HG**	High glucose
**RTECs**	Renal tubular epithelial cells
**VEGF**	vascular endothelial growth factor
**BRB**	blood-retinal barrier
**AGEs**	Advanced glycation end products
**RPE**	Retinal pigment epithelium
**ARPE-19**	Human retinal pigment epithelial
**LMP**	Lysosomal membrane permeability
**HMGB1**	High mobility group box 1
**RGCs**	Retinal ganglion cells
**HBSP**	Helix B surface peptide
**SCs**	Schwann cells
**TSPO**	Translocator protein
**EMT**	Epithelial mesenchymal transition
**MPC5**	Mouse podocytes clone 5
**TG**	Tripterygium glycoside
**RSV**	Resveratrol
**SERCA2b**	Sarcoendoplasmic reticulum Ca2+ ATPase 2b
**AS-IV**	Astragaloside IV
**HO-1**	Heme oxygenase-1
**FA**	Ferulic acid
**NRK-52E**	Normal Rat Kidney-52E
**HMCs**	Human mesangial cells
**DHM**	Dihydromyricetin
**CP**	Cyclocarya paliurus
**CPT**	Cyclocarya paliurus enriches triterpenic acids
**HK-2**	Human renal tubular epithelial
**TGF-bRI**	Transforming growth factor-b type I receptor
**RAGE**	Receptor for advanced glycation endproducts
**DIO**	Diosgenin
**Mel**	Melatonin
**HRMECs**	Human retinal microvascular endothelial cells
**HGY**	HG and hypoxia
**AA**	Arjunolic acid
**PGC-1α**	Peroxisome proliferator activated receptor-gamma coactivator-1α
**LBP**	Lycium barbarum Polysaccharides
**EGCG**	Epigallocatechin-3-gallate
**Gyp-17**	Gypenoside XVII
**SOD**	Superoxide dismutase
**GSH-px**	Glutathione peroxidase
**NGR1**	Notoginsenoside R1
**1,25D3**	1,25-Dihydroxyvitamin-D3
**VDR**	Vitamin D receptor
**BC**	β-carotene
**HOG**	Highly oxidised, glycated
**Hsp70**	Heat Shock Proteins70
**GSK-3β**	Glycogen synthase kinase-3β
**Drp1**	Dynamic-related protein 1
**Mfn1**	Mitofusion 1
**H9c2**	Rat cardiomyocytes
**CX43**	Connexin 43
**TGF-β**	Transforming growth factor-β
**MMPs**	Matrix metalloproteins
**ECM**	Extracellular matrix
**Mst1**	Mammalian Ste20-like kinase 1
**GK**	Goto-Kakazaki
**CA**	Chlorogenic acid
**MMP**	Mitochondrial membrane potential
**RSC96**	Rat Schwann Cell
**MMP9**	Matrix metalloproteinase 9
**N2A**	Neuroblastoma
**TFAM**	Mitochondrial transcription factor A

## References

[B1] Adornetto A. Gesualdo C. Laganà M. L. Trotta M. C. Rossi S. Russo R. (2021). Autophagy: a novel pharmacological target in diabetic retinopathy. Front. Pharmacol. 12, 695267. 10.3389/fphar.2021.695267 34234681 PMC8256993

[B2] Adriaenssens E. Ferrari L. Martens S. (2022). Orchestration of selective autophagy by cargo receptors. Curr. Biol. 32 (24), R1357–r1371. 10.1016/j.cub.2022.11.002 36538890

[B3] Alers S. Löffler A. S. Wesselborg S. Stork B. (2012). Role of AMPK-mTOR-Ulk1/2 in the regulation of autophagy: cross talk, shortcuts, and feedbacks. Mol. Cell Biol. 32 (1), 2–11. 10.1128/MCB.06159-11 22025673 PMC3255710

[B4] Al Mamun A. Ara Mimi A. Wu Y. Zaeem M. Abdul Aziz M. Aktar Suchi S. (2021). Pyroptosis in diabetic nephropathy. Clin. Chim. Acta 523, 131–143. 10.1016/j.cca.2021.09.003 34529985

[B5] Ardeleanu V. Toma A. Pafili K. Papanas N. Motofei I. Diaconu C. C. (2020). Current pharmacological treatment of painful diabetic neuropathy: a narrative review. Med. Kaunas. 56 (1), 25. 10.3390/medicina56010025 PMC7022869 31936646

[B6] Asadi S. Gholami M. S. Siassi F. Qorbani M. Khamoshian K. Sotoudeh G. (2019). Nano curcumin supplementation reduced the severity of diabetic sensorimotor polyneuropathy in patients with type 2 diabetes mellitus: a randomized double-blind placebo-controlled clinical trial. Complement. Ther. Med. 43, 253–260. 10.1016/j.ctim.2019.02.014 30935539

[B7] Bharath L. P. Rockhold J. D. Conway R. (2021). Selective autophagy in hyperglycemia-induced microvascular and macrovascular diseases. Cells 10 (8), 2114. 10.3390/cells10082114 34440882 PMC8392047

[B8] Biasizzo M. Kopitar-Jerala N. (2020). Interplay between NLRP3 inflammasome and autophagy. Front. Immunol. 11, 591803. 10.3389/fimmu.2020.591803 33163006 PMC7583715

[B9] Carlsson S. R. Simonsen A. (2015). Membrane dynamics in autophagosome biogenesis. J. Cell Sci. 128 (2), 193–205. 10.1242/jcs.141036 25568151

[B10] Catalani E. Del Quondam S. Brunetti K. Cherubini A. Bongiorni S. Taddei A. R. (2023). Neuroprotective role of plumbagin on eye damage induced by high-sucrose diet in adult fruit fly *Drosophila melanogaster* . Biomed. Pharmacother. 166, 115298. 10.1016/j.biopha.2023.115298 37597318

[B11] Che H. Li H. Li Y. Wang Y. Q. Yang Z. Y. Wang R. L. (2020). Melatonin exerts neuroprotective effects by inhibiting neuronal pyroptosis and autophagy in STZ-induced diabetic mice. Faseb J. 34 (10), 14042–14054. 10.1096/fj.202001328R 32910484

[B12] Chen D. D. Xu R. Zhou J. Y. Chen J. Q. Wang L. Liu X. S. (2019a). Cordyceps militaris polysaccharides exerted protective effects on diabetic nephropathy in mice via regulation of autophagy. Food Funct. 10 (8), 5102–5114. 10.1039/c9fo00957d 31363726

[B13] Chen F. Sun Z. Zhu X. Ma Y. (2018a). Astilbin inhibits high glucose-induced autophagy and apoptosis through the PI3K/Akt pathway in human proximal tubular epithelial cells. Biomed. Pharmacother. 106, 1175–1181. 10.1016/j.biopha.2018.07.072 30119185

[B14] Chen H. Ji Y. Yan X. Su G. Chen L. Xiao J. (2018b). Berberine attenuates apoptosis in rat retinal Müller cells stimulated with high glucose via enhancing autophagy and the AMPK/mTOR signaling. Biomed. Pharmacother. 108, 1201–1207. 10.1016/j.biopha.2018.09.140 30372821

[B15] Chen J. Zhao D. Zhu M. Zhang M. Hou X. Ding W. (2017). Paeoniflorin ameliorates AGEs-induced mesangial cell injury through inhibiting RAGE/mTOR/autophagy pathway. Biomed. Pharmacother. 89, 1362–1369. 10.1016/j.biopha.2017.03.016 28320103

[B16] Chen Y. Liu Q. Shan Z. Mi W. Zhao Y. Li M. (2019b). Catalpol ameliorates podocyte injury by stabilizing cytoskeleton and enhancing autophagy in diabetic nephropathy. Front. Pharmacol. 10, 1477. 10.3389/fphar.2019.01477 31920663 PMC6914850

[B17] Chen Y. Yu L. (2018). Development of research into autophagic lysosome reformation. Mol. Cells 41 (1), 45–49. 10.14348/molcells.2018.2265 29370688 PMC5792712

[B18] Cheng Z. (2019). The FoxO-autophagy Axis in health and disease. Trends Endocrinol. Metab. 30 (9), 658–671. 10.1016/j.tem.2019.07.009 31443842

[B19] Choi S. J. Kim S. Lee W. S. Kim D. W. Kim C. S. Oh S. H. (2023). Autophagy dysfunction in a diabetic peripheral neuropathy model. Plast. Reconstr. Surg. 151 (2), 355–364. 10.1097/PRS.0000000000009844 36355029

[B20] Choi S. K. Kwon Y. Byeon S. Lee Y. H. (2020). Stimulation of autophagy improves vascular function in the mesenteric arteries of type 2 diabetic mice. Exp. Physiol. 105 (1), 192–200. 10.1113/EP087737 31736185

[B21] Cui D. Qu R. Liu D. Xiong X. Liang T. Zhao Y. (2021). The cross talk between p53 and mTOR pathways in response to physiological and genotoxic stresses. Front. Cell Dev. Biol. 9, 775507. 10.3389/fcell.2021.775507 34869377 PMC8638743

[B22] D'arcy M. S. (2019). Cell death: a review of the major forms of apoptosis, necrosis and autophagy. Cell Biol. Int. 43 (6), 582–592. 10.1002/cbin.11137 30958602

[B23] Dehdashtian E. Mehrzadi S. Yousefi B. Hosseinzadeh A. Reiter R. J. Safa M. (2018). Diabetic retinopathy pathogenesis and the ameliorating effects of melatonin; involvement of autophagy, inflammation and oxidative stress. Life Sci. 193, 20–33. 10.1016/j.lfs.2017.12.001 29203148

[B24] Deretic V. (2021). Autophagy in inflammation, infection, and immunometabolism. Immunity 54 (3), 437–453. 10.1016/j.immuni.2021.01.018 33691134 PMC8026106

[B25] Dewanjee S. Vallamkondu J. Kalra R. S. John A. Reddy P. H. Kandimalla R. (2021). Autophagy in the diabetic heart: a potential pharmacotherapeutic target in diabetic cardiomyopathy. Ageing Res. Rev. 68, 101338. 10.1016/j.arr.2021.101338 33838320

[B26] Dikic I. Elazar Z. (2018). Mechanism and medical implications of mammalian autophagy. Nat. Rev. Mol. Cell Biol. 19 (6), 349–364. 10.1038/s41580-018-0003-4 29618831

[B27] Dong J. Li H. Bai Y. Wu C. (2019a). Muscone ameliorates diabetic peripheral neuropathy through activating AKT/mTOR signalling pathway. J. Pharm. Pharmacol. 71 (11), 1706–1713. 10.1111/jphp.13157 31468549

[B28] Dong Y. Chen H. Gao J. Liu Y. Li J. Wang J. (2019b). Molecular machinery and interplay of apoptosis and autophagy in coronary heart disease. J. Mol. Cell Cardiol. 136, 27–41. 10.1016/j.yjmcc.2019.09.001 31505198

[B29] Dooley H. C. Razi M. Polson H. E. Girardin S. E. Wilson M. I. Tooze S. A. (2014). WIPI2 links LC3 conjugation with PI3P, autophagosome formation, and pathogen clearance by recruiting Atg12-5-16L1. Mol. Cell 55 (2), 238–252. 10.1016/j.molcel.2014.05.021 24954904 PMC4104028

[B234] Dorotea D. Jiang S. Pak E. S. Son J. B. Choi H. G. Ahn S. M. (2022). Pan-Src kinase inhibitor treatment attenuates diabetic kidney injury via inhibition of Fyn kinase-mediated endoplasmic reticulum stress. Exp. Mol. Med. 54 (8), 1086–1097. 10.1038/s12276-022-00810-3 35918533 PMC9440146

[B30] Dusabimana T. Park E. J. Je J. Jeong K. Yun S. P. Kim H. J. (2021). Geniposide improves diabetic nephropathy by enhancing ULK1-mediated autophagy and reducing oxidative stress through AMPK activation. Int. J. Mol. Sci. 22 (4), 1651. 10.3390/ijms22041651 33562139 PMC7915505

[B31] Erekat N. S. (2022). Programmed cell death in diabetic nephropathy: a review of apoptosis, autophagy, and necroptosis. Med. Sci. Monit. 28, e937766. 10.12659/MSM.937766 35989481 PMC9462523

[B32] Ezzat S. M. Abdallah H. M. I. Yassen N. N. Radwan R. A. Mostafa E. S. Salama M. M. (2021). Phenolics from Physalis peruviana fruits ameliorate streptozotocin-induced diabetes and diabetic nephropathy in rats via induction of autophagy and apoptosis regression. Biomed. Pharmacother. 142, 111948. 10.1016/j.biopha.2021.111948 34385108

[B33] Feidantsis K. Mellidis K. Galatou E. Sinakos Z. Lazou A. (2018). Treatment with crocin improves cardiac dysfunction by normalizing autophagy and inhibiting apoptosis in STZ-induced diabetic cardiomyopathy. Nutr. Metab. Cardiovasc Dis. 28 (9), 952–961. 10.1016/j.numecd.2018.06.005 30017436

[B34] Feng L. Liang L. Zhang S. Yang J. Yue Y. Zhang X. (2022). HMGB1 downregulation in retinal pigment epithelial cells protects against diabetic retinopathy through the autophagy-lysosome pathway. Autophagy 18 (2), 320–339. 10.1080/15548627.2021.1926655 34024230 PMC8942416

[B35] Ferdous A. Battiprolu P. K. Ni Y. G. Rothermel B. A. Hill J. A. (2010). FoxO, autophagy, and cardiac remodeling. J. Cardiovasc Transl. Res. 3 (4), 355–364. 10.1007/s12265-010-9200-z 20577843 PMC2994100

[B36] Fu D. Yu J. Y. Connell A. R. Yang S. Hookham M. B. Mcleese R. (2016). Beneficial effects of berberine on oxidized LDL-induced cytotoxicity to human retinal müller cells. Invest. Ophthalmol. Vis. Sci. 57 (7), 3369–3379. 10.1167/iovs.16-19291 27367504 PMC4961062

[B37] Funderburk S. F. Wang Q. J. Yue Z. (2010). The Beclin 1-VPS34 complex--at the crossroads of autophagy and beyond. Trends Cell Biol. 20 (6), 355–362. 10.1016/j.tcb.2010.03.002 20356743 PMC3781210

[B38] Ganesan R. Hos N. J. Gutierrez S. Fischer J. Stepek J. M. Daglidu E. (2017). Salmonella Typhimurium disrupts Sirt1/AMPK checkpoint control of mTOR to impair autophagy. PLoS Pathog. 13 (2), e1006227. 10.1371/journal.ppat.1006227 28192515 PMC5325604

[B39] Gao N. Ma B. Jia H. Hao C. Jin T. Liu X. (2023). Translocator protein alleviates allodynia and improves Schwann cell function against diabetic peripheral neuropathy via activation of the Nrf2-dependent antioxidant system and promoting autophagy. Diabet. Med. 40 (6), e15090. 10.1111/dme.15090 37013248

[B40] Gautam S. Zhang L. Arnaoutova I. Lee C. Mansfield B. C. Chou J. Y. (2020). The signaling pathways implicated in impairment of hepatic autophagy in glycogen storage disease type Ia. Hum. Mol. Genet. 29 (5), 834–844. 10.1093/hmg/ddaa007 31961433 PMC7104680

[B41] Glick D. Barth S. Macleod K. F. (2010). Autophagy: cellular and molecular mechanisms. J. Pathol. 221 (1), 3–12. 10.1002/path.2697 20225336 PMC2990190

[B42] Gong Q. Wang H. Yu P. Qian T. Xu X. (2021). Protective or harmful: the dual roles of autophagy in diabetic retinopathy. Front. Med. (Lausanne) 8, 644121. 10.3389/fmed.2021.644121 33842506 PMC8026897

[B43] Gonzalez C. D. Carro Negueruela M. P. Nicora Santamarina C. Resnik R. Vaccaro M. I. (2021). Autophagy dysregulation in diabetic kidney disease: from pathophysiology to pharmacological interventions. Cells 10 (9), 2497. 10.3390/cells10092497 34572148 PMC8469825

[B44] Gowd V. Kang Q. Wang Q. Wang Q. Chen F. Cheng K. W. (2020). Resveratrol: evidence for its nephroprotective effect in diabetic nephropathy. Adv. Nutr. 11 (6), 1555–1568. 10.1093/advances/nmaa075 32577714 PMC7666903

[B45] Graef M. Friedman J. R. Graham C. Babu M. Nunnari J. (2013). ER exit sites are physical and functional core autophagosome biogenesis components. Mol. Biol. Cell 24 (18), 2918–2931. 10.1091/mbc.E13-07-0381 23904270 PMC3771953

[B46] Gross S. P. Vershinin M. Shubeita G. T. (2007). Cargo transport: two motors are sometimes better than one. Curr. Biol. 17 (12), R478–R486. 10.1016/j.cub.2007.04.025 17580082

[B47] Guo H. Wang Y. Zhang X. Zang Y. Zhang Y. Wang L. (2017). Astragaloside IV protects against podocyte injury via SERCA2-dependent ER stress reduction and AMPKα-regulated autophagy induction in streptozotocin-induced diabetic nephropathy. Sci. Rep. 7 (1), 6852. 10.1038/s41598-017-07061-7 28761152 PMC5537362

[B48] Guo L. Tan K. Luo Q. Bai X. (2020a). Dihydromyricetin promotes autophagy and attenuates renal interstitial fibrosis by regulating miR-155-5p/PTEN signaling in diabetic nephropathy. Bosn. J. Basic Med. Sci. 20 (3), 372–380. 10.17305/bjbms.2019.4410 31668144 PMC7416184

[B49] Guo X. Lin H. Liu J. Wang D. Li D. Jiang C. (2020b). 1,25-Dihydroxyvitamin D attenuates diabetic cardiac autophagy and damage by vitamin D receptor-mediated suppression of FoxO1 translocation. J. Nutr. Biochem. 80, 108380. 10.1016/j.jnutbio.2020.108380 32299030

[B50] Habshi T. Shelke V. Kale A. Anders H. J. Gaikwad A. B. (2023). Role of endoplasmic reticulum stress and autophagy in the transition from acute kidney injury to chronic kidney disease. J. Cell Physiol. 238 (1), 82–93. 10.1002/jcp.30918 36409755

[B51] Han S. Li X. Liang X. Zhou L. (2019). HOXA9 transcriptionally promotes apoptosis and represses autophagy by targeting NF-κB in cutaneous squamous cell carcinoma. Cells 8 (11), 1360. 10.3390/cells8111360 31683603 PMC6912505

[B52] Hang W. He B. Chen J. Xia L. Wen B. Liang T. (2018). Berberine ameliorates high glucose-induced cardiomyocyte injury via AMPK signaling activation to stimulate mitochondrial biogenesis and restore autophagic flux. Front. Pharmacol. 9, 1121. 10.3389/fphar.2018.01121 30337876 PMC6178920

[B53] Hao R. Sun B. Yang L. Ma C. Li S. (2020). RVG29-modified microRNA-loaded nanoparticles improve ischemic brain injury by nasal delivery. Drug Deliv. 27 (1), 772–781. 10.1080/10717544.2020.1760960 32400219 PMC7269067

[B54] Hao Y. Gao X. (2022). Diosgenin protects retinal pigment epithelial cells from inflammatory damage and oxidative stress induced by high glucose by activating AMPK/Nrf2/HO-1 pathway. Immun. Inflamm. Dis. 10 (12), e698. 10.1002/iid3.698 36444632 PMC9667204

[B55] Hinchy E. C. Gruszczyk A. V. Willows R. Navaratnam N. Hall A. R. Bates G. (2018). Mitochondria-derived ROS activate AMP-activated protein kinase (AMPK) indirectly. J. Biol. Chem. 293 (44), 17208–17217. 10.1074/jbc.RA118.002579 30232152 PMC6222118

[B56] Hou H. Zhang Q. Dong H. Ge Z. (2019). Matrine improves diabetic cardiomyopathy through TGF-β-induced protein kinase RNA-like endoplasmic reticulum kinase signaling pathway. J. Cell Biochem. 120 (8), 13573–13582. 10.1002/jcb.28632 30938856

[B57] Huang G. Zou B. Lv J. Li T. Huai G. Xiang S. (2017a). Notoginsenoside R1 attenuates glucose-induced podocyte injury via the inhibition of apoptosis and the activation of autophagy through the PI3K/Akt/mTOR signaling pathway. Int. J. Mol. Med. 39 (3), 559–568. 10.3892/ijmm.2017.2864 28112381 PMC5360354

[B58] Huang S. S. Ding D. F. Chen S. Dong C. L. Ye X. L. Yuan Y. G. (2017b). Resveratrol protects podocytes against apoptosis via stimulation of autophagy in a mouse model of diabetic nephropathy. Sci. Rep. 7, 45692. 10.1038/srep45692 28374806 PMC5379482

[B59] Hubert V. Weiss S. Rees A. J. Kain R. (2022). Modulating chaperone-mediated autophagy and its clinical applications in cancer. Cells 11 (16), 2562. 10.3390/cells11162562 36010638 PMC9406970

[B60] Inoki K. Ouyang H. Zhu T. Lindvall C. Wang Y. Zhang X. (2006). TSC2 integrates Wnt and energy signals via a coordinated phosphorylation by AMPK and GSK3 to regulate cell growth. Cell 126 (5), 955–968. 10.1016/j.cell.2006.06.055 16959574

[B61] Jia G. Hill M. A. Sowers J. R. (2018). Diabetic cardiomyopathy: an update of mechanisms contributing to this clinical entity. Circ. Res. 122 (4), 624–638. 10.1161/CIRCRESAHA.117.311586 29449364 PMC5819359

[B62] Jia Q. Yang R. Mehmood S. Li Y. (2022). Epigallocatechin-3-gallate attenuates myocardial fibrosis in diabetic rats by activating autophagy. Exp. Biol. Med. (Maywood) 247 (17), 1591–1600. 10.1177/15353702221110646 35833541 PMC9554167

[B63] Jin J. Shi Y. Gong J. Zhao L. Li Y. He Q. (2019). Exosome secreted from adipose-derived stem cells attenuates diabetic nephropathy by promoting autophagy flux and inhibiting apoptosis in podocyte. Stem Cell Res. Ther. 10 (1), 95. 10.1186/s13287-019-1177-1 30876481 PMC6419838

[B64] Jin Y. Liu S. Ma Q. Xiao D. Chen L. (2017). Berberine enhances the AMPK activation and autophagy and mitigates high glucose-induced apoptosis of mouse podocytes. Eur. J. Pharmacol. 794, 106–114. 10.1016/j.ejphar.2016.11.037 27887947

[B65] Jing K. Lim K. (2012). Why is autophagy important in human diseases? Exp. Mol. Med. 44 (2), 69–72. 10.3858/emm.2012.44.2.028 22257881 PMC3296814

[B66] Jung H. S. Lee M. S. (2010). Role of autophagy in diabetes and mitochondria. Ann. N. Y. Acad. Sci. 1201, 79–83. 10.1111/j.1749-6632.2010.05614.x 20649543

[B67] Jung S. Jeong H. Yu S. W. (2020). Autophagy as a decisive process for cell death. Exp. Mol. Med. 52 (6), 921–930. 10.1038/s12276-020-0455-4 32591647 PMC7338414

[B68] Kanamori H. Naruse G. Yoshida A. Minatoguchi S. Watanabe T. Kawaguchi T. (2019). Metformin enhances autophagy and provides cardioprotection in δ-sarcoglycan deficiency-induced dilated cardiomyopathy. Circ. Heart Fail 12 (4), e005418. 10.1161/CIRCHEARTFAILURE.118.005418 30922066

[B69] Kanamori H. Takemura G. Goto K. Tsujimoto A. Mikami A. Ogino A. (2015). Autophagic adaptations in diabetic cardiomyopathy differ between type 1 and type 2 diabetes. Autophagy 11 (7), 1146–1160. 10.1080/15548627.2015.1051295 26042865 PMC4590644

[B70] Kast D. J. Dominguez R. (2017). The cytoskeleton-autophagy connection. Curr. Biol. 27 (8), R318–R326. 10.1016/j.cub.2017.02.061 28441569 PMC5444402

[B71] Kaushik S. Cuervo A. M. (2018). The coming of age of chaperone-mediated autophagy. Nat. Rev. Mol. Cell Biol. 19 (6), 365–381. 10.1038/s41580-018-0001-6 29626215 PMC6399518

[B72] Khan I. Preeti K. Kumar R. Kumar Khatri D. Bala Singh S. (2023). Piceatannol promotes neuroprotection by inducing mitophagy and mitobiogenesis in the experimental diabetic peripheral neuropathy and hyperglycemia-induced neurotoxicity. Int. Immunopharmacol. 116, 109793. 10.1016/j.intimp.2023.109793 36731149

[B73] Kim J. H. Choi T. G. Park S. Yun H. R. Nguyen N. N. Y. Jo Y. H. (2018). Mitochondrial ROS-derived PTEN oxidation activates PI3K pathway for mTOR-induced myogenic autophagy. Cell Death Differ. 25 (11), 1921–1937. 10.1038/s41418-018-0165-9 30042494 PMC6219511

[B74] Kimura S. Noda T. Yoshimori T. (2008). Dynein-dependent movement of autophagosomes mediates efficient encounters with lysosomes. Cell Struct. Funct. 33 (1), 109–122. 10.1247/csf.08005 18388399

[B75] Kimura T. Isaka Y. Yoshimori T. (2017). Autophagy and kidney inflammation. Autophagy 13 (6), 997–1003. 10.1080/15548627.2017.1309485 28441075 PMC5486362

[B76] Kitada M. Ogura Y. Suzuki T. Sen S. Lee S. M. Kanasaki K. (2016). A very-low-protein diet ameliorates advanced diabetic nephropathy through autophagy induction by suppression of the mTORC1 pathway in Wistar fatty rats, an animal model of type 2 diabetes and obesity. Diabetologia 59 (6), 1307–1317. 10.1007/s00125-016-3925-4 27020449

[B77] Kobayashi S. Liang Q. (2015). Autophagy and mitophagy in diabetic cardiomyopathy. Biochim. Biophys. Acta 1852 (2), 252–261. 10.1016/j.bbadis.2014.05.020 24882754

[B78] Kobayashi S. Xu X. Chen K. Liang Q. (2012). Suppression of autophagy is protective in high glucose-induced cardiomyocyte injury. Autophagy 8 (4), 577–592. 10.4161/auto.18980 22498478 PMC3405845

[B79] Komalla V. Sheikholeslami B. Li G. Bokshi B. Chan Y. L. Ung A. (2020). Impact of A Cargo-Less liposomal formulation on dietary obesity-related metabolic disorders in mice. Int. J. Mol. Sci. 21 (20), 7640. 10.3390/ijms21207640 33076522 PMC7589567

[B80] Kong Z. Lv W. Wang Y. Huang Y. Che K. Nan H. (2023a). Sinensetin ameliorates high glucose-induced diabetic nephropathy via enhancing autophagy *in vitro* and *in vivo* . J. Biochem. Mol. Toxicol. 37 (10), e23445. 10.1002/jbt.23445 37393522

[B81] Kong Z. Xiao M. Wang B. Zhang W. Che K. Lv W. (2023b). Renoprotective effect of isoorientin in diabetic nephropathy via activating autophagy and inhibiting the PI3K-AKT-TSC2-mTOR pathway. Am. J. Chin. Med. 51 (5), 1269–1291. 10.1142/S0192415X23500581 37335208

[B82] Korolchuk V. I. Rubinsztein D. C. (2011). Regulation of autophagy by lysosomal positioning. Autophagy 7 (8), 927–928. 10.4161/auto.7.8.15862 21521941 PMC3149695

[B83] Korolchuk V. I. Saiki S. Lichtenberg M. Siddiqi F. H. Roberts E. A. Imarisio S. (2011). Lysosomal positioning coordinates cellular nutrient responses. Nat. Cell Biol. 13 (4), 453–460. 10.1038/ncb2204 21394080 PMC3071334

[B84] Kume S. Koya D. (2015). Autophagy: a novel therapeutic target for diabetic nephropathy. Diabetes Metab. J. 39 (6), 451–460. 10.4093/dmj.2015.39.6.451 26706914 PMC4696980

[B85] Lapaquette P. Guzzo J. Bretillon L. Bringer M. A. (2015). Cellular and molecular connections between autophagy and inflammation. Mediat. Inflamm. 2015, 398483. 10.1155/2015/398483 PMC4499609 26221063

[B86] Lee I. H. (2019). Mechanisms and disease implications of sirtuin-mediated autophagic regulation. Exp. Mol. Med. 51 (9), 1–11. 10.1038/s12276-019-0302-7 PMC6802627 31492861

[B87] Lee M. J. Park J. S. Jo S. B. Joe Y. A. (2023). Enhancing anti-cancer therapy with selective autophagy inhibitors by targeting protective autophagy. Biomol. Ther. Seoul. 31 (1), 1–15. 10.4062/biomolther.2022.153 36579459 PMC9810440

[B88] Lee T. I. Bai K. J. Chen Y. C. Lee T. W. Chung C. C. Tsai W. C. (2018). Histone deacetylase inhibition of cardiac autophagy in rats on a high-fat diet with low-dose streptozotocin-induced type 2 diabetes mellitus. Mol. Med. Rep. 17 (1), 594–601. 10.3892/mmr.2017.7905 29115461

[B89] Leventhal J. S. Wyatt C. M. Ross M. J. (2017). Recycling to discover something new: the role of autophagy in kidney disease. Kidney Int. 91 (1), 4–6. 10.1016/j.kint.2016.11.004 28003091

[B90] Li C. Guan X. M. Wang R. Y. Xie Y. S. Zhou H. Ni W. J. (2020a). Berberine mitigates high glucose-induced podocyte apoptosis by modulating autophagy via the mTOR/P70S6K/4EBP1 pathway. Life Sci. 243, 117277. 10.1016/j.lfs.2020.117277 31926252

[B91] Li J. Chen X. Kang R. Zeh H. Klionsky D. J. Tang D. (2021a). Regulation and function of autophagy in pancreatic cancer. Autophagy 17 (11), 3275–3296. 10.1080/15548627.2020.1847462 33161807 PMC8632104

[B92] Li J. Yu S. Ying J. Shi T. Wang P. (2017a). Resveratrol prevents ROS-induced apoptosis in high glucose-treated retinal capillary endothelial cells via the activation of AMPK/Sirt1/PGC-1α pathway. Oxid. Med. Cell Longev. 2017, 7584691. 10.1155/2017/7584691 29213353 PMC5682085

[B93] Li L. Chen J. Zhou Y. Zhang J. Chen L. (2023). Artesunate alleviates diabetic retinopathy by activating autophagy via the regulation of AMPK/SIRT1 pathway. Arch. Physiol. Biochem. 129 (4), 943–950. 10.1080/13813455.2021.1887266 33661722

[B94] Li L. Tan J. Miao Y. Lei P. Zhang Q. (2015). ROS and autophagy: interactions and molecular regulatory mechanisms. Cell Mol. Neurobiol. 35 (5), 615–621. 10.1007/s10571-015-0166-x 25722131 PMC11486209

[B95] Li R. Chen L. Yao G. M. Yan H. L. Wang L. (2021b). Effects of quercetin on diabetic retinopathy and its association with NLRP3 inflammasome and autophagy. Int. J. Ophthalmol. 14 (1), 42–49. 10.18240/ijo.2021.01.06 33469482 PMC7790678

[B96] Li R. Li H. Zhang Q. (2022). Procyanidin protects human retinal pigment epithelial cells from high glucose by inhibiting autophagy. Environ. Toxicol. 37 (2), 201–211. 10.1002/tox.23389 34636125

[B97] Li X. Zhu Q. Zheng R. Yan J. Wei M. Fan Y. (2020b). Puerarin attenuates diabetic nephropathy by promoting autophagy in podocytes. Front. Physiol. 11, 73. 10.3389/fphys.2020.00073 32116781 PMC7033627

[B98] Li X. Y. Wang S. S. Han Z. Han F. Chang Y. P. Yang Y. (2017b). Triptolide restores autophagy to alleviate diabetic renal fibrosis through the miR-141-3p/PTEN/Akt/mTOR pathway. Mol. Ther. Nucleic Acids 9, 48–56. 10.1016/j.omtn.2017.08.011 29246323 PMC5602517

[B99] Li X. Z. Jiang H. Xu L. Liu Y. Q. Tang J. W. Shi J. S. (2021c). Sarsasapogenin restores podocyte autophagy in diabetic nephropathy by targeting GSK3β signaling pathway. Biochem. Pharmacol. 192, 114675. 10.1016/j.bcp.2021.114675 34252407

[B100] Liang R. K. Zhao Y. Y. Shi M. L. Zhang G. Zhao Y. J. Zhang B. G. (2021). Skimmin protects diabetic cardiomyopathy in streptozotocin-induced diabetic rats. Kaohsiung J. Med. Sci. 37 (2), 136–144. 10.1002/kjm2.12305 33128488 PMC11896253

[B101] Liao M. F. Lu K. T. Hsu J. L. Lee C. H. Cheng M. Y. Ro L. S. (2022). The role of autophagy and apoptosis in neuropathic pain formation. Int. J. Mol. Sci. 23 (5), 2685. 10.3390/ijms23052685 35269822 PMC8910267

[B102] Lin C. Zhang M. Zhang Y. Yang K. Hu J. Si R. (2017). Helix B surface peptide attenuates diabetic cardiomyopathy via AMPK-dependent autophagy. Biochem. Biophys. Res. Commun. 482 (4), 665–671. 10.1016/j.bbrc.2016.11.091 27865838

[B103] Lin W. Xu G. (2019). Autophagy: a role in the apoptosis, survival, inflammation, and development of the retina. Ophthalmic Res. 61 (2), 65–72. 10.1159/000487486 29694961

[B104] Liu H. Wang Q. Shi G. Yang W. Zhang Y. Chen W. (2021). Emodin ameliorates renal damage and podocyte injury in a rat model of diabetic nephropathy via regulating AMPK/mTOR-Mediated autophagy signaling pathway. Diabetes Metab. Syndr. Obes. 14, 1253–1266. 10.2147/DMSO.S299375 33776462 PMC7987270

[B105] Liu J. Tang Y. Feng Z. Hou C. Wang H. Yan J. (2014a). Acetylated FoxO1 mediates high-glucose induced autophagy in H9c2 cardiomyoblasts: regulation by a polyphenol -(-)-epigallocatechin-3-gallate. Metabolism 63 (10), 1314–1323. 10.1016/j.metabol.2014.06.012 25062567

[B106] Liu J. Tang Y. Feng Z. Liu J. Liu J. Long J. (2014b). (-)-Epigallocatechin-3-gallate attenuated myocardial mitochondrial dysfunction and autophagy in diabetic Goto-Kakizaki rats. Free Radic. Res. 48 (8), 898–906. 10.3109/10715762.2014.920955 24797301

[B107] Liu L. Bai F. Song H. Xiao R. Wang Y. Yang H. (2022a). Upregulation of TIPE1 in tubular epithelial cell aggravates diabetic nephropathy by disrupting PHB2 mediated mitophagy. Redox Biol. 50, 102260. 10.1016/j.redox.2022.102260 35152003 PMC8844679

[B108] Liu P. Zhu W. Wang Y. Ma G. Zhao H. Li P. (2023a). Chinese herbal medicine and its active compounds in attenuating renal injury *via* regulating autophagy in diabetic kidney disease. Front. Endocrinol. (Lausanne) 14, 1142805. 10.3389/fendo.2023.1142805 36942026 PMC10023817

[B109] Liu S. Y. Chen L. Li X. C. Hu Q. K. He L. J. (2018). Lycium barbarum polysaccharide protects diabetic peripheral neuropathy by enhancing autophagy via mTOR/p70S6K inhibition in Streptozotocin-induced diabetic rats. J. Chem. Neuroanat. 89, 37–42. 10.1016/j.jchemneu.2017.12.011 29294366

[B110] Liu T. Jin Q. Yang L. Mao H. Ma F. Wang Y. (2023b). Regulation of autophagy by natural polyphenols in the treatment of diabetic kidney disease: therapeutic potential and mechanism. Front. Endocrinol. (Lausanne) 14, 1142276. 10.3389/fendo.2023.1142276 37635982 PMC10448531

[B111] Liu W. J. Gan Y. Huang W. F. Wu H. L. Zhang X. Q. Zheng H. J. (2019). Lysosome restoration to activate podocyte autophagy: a new therapeutic strategy for diabetic kidney disease. Cell Death Dis. 10 (11), 806. 10.1038/s41419-019-2002-6 31649253 PMC6813305

[B112] Liu X. Q. Jiang L. Li Y. Y. Huang Y. B. Hu X. R. Zhu W. (2022b). Wogonin protects glomerular podocytes by targeting Bcl-2-mediated autophagy and apoptosis in diabetic kidney disease. Acta Pharmacol. Sin. 43 (1), 96–110. 10.1038/s41401-021-00721-5 34253875 PMC8724322

[B113] Lopes De Faria J. M. Duarte D. A. Montemurro C. Papadimitriou A. Consonni S. R. Lopes De Faria J. B. (2016). Defective autophagy in diabetic retinopathy. Invest. Ophthalmol. Vis. Sci. 57 (10), 4356–4366. 10.1167/iovs.16-19197 27564518

[B114] Lu Y. Li Z. Zhang S. Zhang T. Liu Y. Zhang L. (2023). Cellular mitophagy: mechanism, roles in diseases and small molecule pharmacological regulation. Theranostics 13 (2), 736–766. 10.7150/thno.79876 36632220 PMC9830443

[B115] Lu Y. Liu Y. Li H. Wang X. Wu W. Gao L. (2015). Effect and mechanisms of zinc supplementation in protecting against diabetic cardiomyopathy in a rat model of type 2 diabetes. Bosn. J. Basic Med. Sci. 15 (1), 14–20. 10.17305/bjbms.2015.63 PMC4365671 25725139

[B116] Luo Y. Dong X. Lu S. Gao Y. Sun G. Sun X. (2021). Gypenoside XVII alleviates early diabetic retinopathy by regulating Müller cell apoptosis and autophagy in db/db mice. Eur. J. Pharmacol. 895, 173893. 10.1016/j.ejphar.2021.173893 33493483

[B117] Ma C. Zhang D. Ma Q. Liu Y. Yang Y. (2021). Arbutin inhibits inflammation and apoptosis by enhancing autophagy via SIRT1. Adv. Clin. Exp. Med. 30 (5), 535–544. 10.17219/acem/133493 33974755

[B118] Ma L. Fu R. Duan Z. Lu J. Gao J. Tian L. (2016). Sirt1 is essential for resveratrol enhancement of hypoxia-induced autophagy in the type 2 diabetic nephropathy rat. Pathol. Res. Pract. 212 (4), 310–318. 10.1016/j.prp.2016.02.001 26872534

[B119] Ma R. He Y. Fang Q. Xie G. Qi M. (2022). Ferulic acid ameliorates renal injury via improving autophagy to inhibit inflammation in diabetic nephropathy mice. Biomed. Pharmacother. 153, 113424. 10.1016/j.biopha.2022.113424 36076545

[B120] Ma Z. Li L. Livingston M. J. Zhang D. Mi Q. Zhang M. (2020). p53/microRNA-214/ULK1 axis impairs renal tubular autophagy in diabetic kidney disease. J. Clin. Invest. 130 (9), 5011–5026. 10.1172/JCI135536 32804155 PMC7456229

[B121] Madrakhimov S. B. Yang J. Y. Kim J. H. Han J. W. Park T. K. (2021). mTOR-dependent dysregulation of autophagy contributes to the retinal ganglion cell loss in streptozotocin-induced diabetic retinopathy. Cell Commun. Signal 19 (1), 29. 10.1186/s12964-020-00698-4 33637094 PMC7913405

[B122] Maiuri M. C. Zalckvar E. Kimchi A. Kroemer G. (2007). Self-eating and self-killing: crosstalk between autophagy and apoptosis. Nat. Rev. Mol. Cell Biol. 8 (9), 741–752. 10.1038/nrm2239 17717517

[B123] Mariño G. Niso-Santano M. Baehrecke E. H. Kroemer G. (2014). Self-consumption: the interplay of autophagy and apoptosis. Nat. Rev. Mol. Cell Biol. 15 (2), 81–94. 10.1038/nrm3735 24401948 PMC3970201

[B124] Matboli M. Ibrahim D. Hasanin A. H. Hassan M. K. Habib E. K. Bekhet M. M. (2021). Epigenetic modulation of autophagy genes linked to diabetic nephropathy by administration of isorhamnetin in Type 2 diabetes mellitus rats. Epigenomics 13 (3), 187–202. 10.2217/epi-2020-0353 33406900

[B126] Medras Z. J. H. Mostafa Y. M. Ahmed A. a. M. El-Sayed N. M. (2023). Arctigenin improves neuropathy via ameliorating apoptosis and modulating autophagy in streptozotocin-induced diabetic mice. CNS Neurosci. Ther. 29 (10), 3068–3080. 10.1111/cns.14249 37170684 PMC10493658

[B127] Mizushima N. Komatsu M. (2011). Autophagy: renovation of cells and tissues. Cell 147 (4), 728–741. 10.1016/j.cell.2011.10.026 22078875

[B128] Morishita H. Kaizuka T. Hama Y. Mizushima N. (2017). A new probe to measure autophagic flux *in vitro* and *in vivo* . Autophagy 13 (4), 757–758. 10.1080/15548627.2016.1278094 28121224 PMC5388228

[B129] Nellaiappan K. Preeti K. Khatri D. K. Singh S. B. (2022). Diabetic complications: an update on pathobiology and therapeutic strategies. Curr. Diabetes Rev. 18 (1), e030821192146. 10.2174/1573399817666210309104203 33745424

[B130] Newman D. J. Cragg G. M. (2016). Natural products as sources of new drugs from 1981 to 2014. J. Nat. Prod. 79 (3), 629–661. 10.1021/acs.jnatprod.5b01055 26852623

[B131] Newman D. J. Cragg G. M. (2020). Natural products as sources of new drugs over the nearly four decades from 01/1981 to 09/2019. J. Nat. Prod. 83 (3), 770–803. 10.1021/acs.jnatprod.9b01285 32162523

[B132] Ni T. Lin N. Lu W. Sun Z. Lin H. Chi J. (2020). Dihydromyricetin prevents diabetic cardiomyopathy via miR-34a suppression by activating autophagy. Cardiovasc Drugs Ther. 34 (3), 291–301. 10.1007/s10557-020-06968-0 32212062

[B133] Ou M. Huang R. Yang C. Gui B. Luo Q. Zhao J. (2021). Chromosome-level genome assemblies of Channa argusandChanna maculata and comparative analysis of their temperature adaptability. Gigascience 10 (10), giab070. 10.1093/gigascience/giab070 34673930 PMC8529964

[B134] Oza M. J. Laddha A. P. Gaikwad A. B. Mulay S. R. Kulkarni Y. A. (2021). Role of dietary modifications in the management of type 2 diabetic complications. Pharmacol. Res. 168, 105602. 10.1016/j.phrs.2021.105602 33838293

[B135] Park S. Lim Y. Lee D. Elvira R. Lee J. M. Lee M. R. (2018). Modulation of protein synthesis by eIF2α phosphorylation protects cell from Heat stress-mediated apoptosis. Cells 7 (12), 254. 10.3390/cells7120254 30544621 PMC6316477

[B136] Parzych K. R. Klionsky D. J. (2014). An overview of autophagy: morphology, mechanism, and regulation. Antioxid. Redox Signal 20 (3), 460–473. 10.1089/ars.2013.5371 23725295 PMC3894687

[B137] Peng H. Han W. Ma B. Dai S. Long J. Zhou S. (2022). Autophagy and senescence of rat retinal precursor cells under high glucose. Front. Endocrinol. (Lausanne) 13, 1047642. 10.3389/fendo.2022.1047642 36686430 PMC9846177

[B138] Pohl C. Dikic I. (2019). Cellular quality control by the ubiquitin-proteasome system and autophagy. Science 366 (6467), 818–822. 10.1126/science.aax3769 31727826

[B139] Preetha Rani M. R. Salin Raj P. Nair A. Ranjith S. Rajankutty K. Raghu K. G. (2022). *In vitro* and *in vivo* studies reveal the beneficial effects of chlorogenic acid against ER stress mediated ER-phagy and associated apoptosis in the heart of diabetic rat. Chem. Biol. Interact. 351, 109755. 10.1016/j.cbi.2021.109755 34801538

[B140] Puthanveetil P. Wan A. Rodrigues B. (2013). FoxO1 is crucial for sustaining cardiomyocyte metabolism and cell survival. Cardiovasc Res. 97 (3), 393–403. 10.1093/cvr/cvs426 23263330

[B141] Qu L. Liang X. Gu B. Liu W. (2014). Quercetin alleviates high glucose-induced Schwann cell damage by autophagy. Neural Regen. Res. 9 (12), 1195–1203. 10.4103/1673-5374.135328 25206782 PMC4146282

[B142] Russo R. Berliocchi L. Adornetto A. Amantea D. Nucci C. Tassorelli C. (2013). In search of new targets for retinal neuroprotection: is there a role for autophagy? Curr. Opin. Pharmacol. 13 (1), 72–77. 10.1016/j.coph.2012.09.004 23036350

[B143] Russo S. B. Baicu C. F. Van Laer A. Geng T. Kasiganesan H. Zile M. R. (2012). Ceramide synthase 5 mediates lipid-induced autophagy and hypertrophy in cardiomyocytes. J. Clin. Invest. 122 (11), 3919–3930. 10.1172/JCI63888 23023704 PMC3484448

[B144] Salem H. M. A. Chok K. C. Koh R. Y. Ng P. Y. Tiong Y. L. Chye S. M. (2023). Melatonin ameliorates high glucose-induced autophagy in Schwann cells. Int. J. Biochem. Mol. Biol. 14 (3), 25–31. 37456910 PMC10349298

[B145] Sanchez-Wandelmer J. Ktistakis N. T. Reggiori F. (2015). ERES: sites for autophagosome biogenesis and maturation? J. Cell Sci. 128 (2), 185–192. 10.1242/jcs.158758 25568152

[B146] Scherz-Shouval R. Shvets E. Fass E. Shorer H. Gil L. Elazar Z. (2007). Reactive oxygen species are essential for autophagy and specifically regulate the activity of Atg4. Embo J. 26 (7), 1749–1760. 10.1038/sj.emboj.7601623 17347651 PMC1847657

[B147] Schiborn C. Schulze M. B. (2022). Precision prognostics for the development of complications in diabetes. Diabetologia 65 (11), 1867–1882. 10.1007/s00125-022-05731-4 35727346 PMC9522742

[B148] Schuck S. (2020). Microautophagy - distinct molecular mechanisms handle cargoes of many sizes. J. Cell Sci. 133 (17), jcs246322. 10.1242/jcs.246322 32907930

[B149] Senft D. Ronai Z. A. (2015). UPR, autophagy, and mitochondria crosstalk underlies the ER stress response. Trends Biochem. Sci. 40 (3), 141–148. 10.1016/j.tibs.2015.01.002 25656104 PMC4340752

[B150] Sheng H. Zhang D. Zhang J. Zhang Y. Lu Z. Mao W. (2022). Kaempferol attenuated diabetic nephropathy by reducing apoptosis and promoting autophagy through AMPK/mTOR pathways. Front. Med. (Lausanne) 9, 986825. 10.3389/fmed.2022.986825 36530875 PMC9748551

[B151] Sherkhane B. Yerra V. G. Sharma A. Kumar K. A. Chayanika G. Kumar A. V. (2023). Nephroprotective potential of syringic acid in experimental diabetic nephropathy: focus on oxidative stress and autophagy. Indian J. Pharmacol. 55 (1), 34–42. 10.4103/ijp.ijp_671_22 36960519 PMC10204897

[B152] Shi Y. Gao Y. Wang T. Wang X. He J. Xu J. (2020). Ginsenoside Rg1 alleviates podocyte EMT passage by regulating AKT/GSK3 β/β-Catenin pathway by restoring autophagic activity. Evid. Based Complement. Altern. Med. 2020, 1903627. 10.1155/2020/1903627 PMC7011395 32082395

[B153] Shibutani S. T. Yoshimori T. (2014). A current perspective of autophagosome biogenesis. Cell Res. 24 (1), 58–68. 10.1038/cr.2013.159 24296784 PMC3879706

[B154] Singh A. D. Kulkarni Y. A. (2022). Vascular adhesion protein-1 and microvascular diabetic complications. Pharmacol. Rep. 74 (1), 40–46. 10.1007/s43440-021-00343-y 35001320

[B155] Song A. Zhang C. Meng X. (2021a). Mechanism and application of metformin in kidney diseases: an update. Biomed. Pharmacother. 138, 111454. 10.1016/j.biopha.2021.111454 33714781

[B156] Song C. Gong Z. Ji Y. (2023). Rutaecarpine ameliorates cardiomyocyte injury induced by high glucose by promoting TRPV1-mediated autophagy. Bratisl. Lek. Listy 124 (9), 699–706. 10.4149/BLL_2023_107 37635668

[B157] Song S. Bao S. Zhang C. Zhang J. Lv J. Li X. (2021b). Stimulation of AMPK prevents diabetes-induced photoreceptor cell degeneration. Oxid. Med. Cell Longev. 2021, 5587340. 10.1155/2021/5587340 34093959 PMC8140850

[B158] Su P. P. Liu D. W. Zhou S. J. Chen H. Wu X. M. Liu Z. S. (2022a). Down-regulation of Risa improves podocyte injury by enhancing autophagy in diabetic nephropathy. Mil. Med. Res. 9 (1), 23. 10.1186/s40779-022-00385-0 35614465 PMC9134699

[B159] Su Y. Fan X. Li S. Li Z. Tian M. Li S. (2022b). Scutellarin improves type 2 diabetic cardiomyopathy by regulating cardiomyocyte autophagy and apoptosis. Dis. Markers 2022, 3058354. 10.1155/2022/3058354 35571612 PMC9106511

[B160] Sun S. Yang S. An N. Wang G. Xu Q. Liu J. (2019). Astragalus polysaccharides inhibits cardiomyocyte apoptosis during diabetic cardiomyopathy via the endoplasmic reticulum stress pathway. J. Ethnopharmacol. 238, 111857. 10.1016/j.jep.2019.111857 30959142

[B161] Tan Y. Zhang Z. Zheng C. Wintergerst K. A. Keller B. B. Cai L. (2020). Mechanisms of diabetic cardiomyopathy and potential therapeutic strategies: preclinical and clinical evidence. Nat. Rev. Cardiol. 17 (9), 585–607. 10.1038/s41569-020-0339-2 32080423 PMC7849055

[B162] Tang H. Yang M. Liu Y. Zhu X. Liu S. Liu H. (2022). Melatonin alleviates renal injury by activating mitophagy in diabetic nephropathy. Front. Endocrinol. (Lausanne) 13, 889729. 10.3389/fendo.2022.889729 35992101 PMC9388821

[B163] Tao M. Zheng D. Liang X. Wu D. Hu K. Jin J. (2021). Tripterygium glycoside suppresses epithelial-to-mesenchymal transition of diabetic kidney disease podocytes by targeting autophagy through the mTOR/Twist1 pathway. Mol. Med. Rep. 24 (2), 592. 10.3892/mmr.2021.12231 34165172 PMC8222798

[B164] Tong F. Liu S. Yan B. Li X. Ruan S. Yang S. (2017). Quercetin nanoparticle complex attenuated diabetic nephropathy via regulating the expression level of ICAM-1 on endothelium. Int. J. Nanomedicine 12, 7799–7813. 10.2147/IJN.S146978 29123394 PMC5661459

[B165] Tooze S. A. (2013). Current views on the source of the autophagosome membrane. Essays Biochem. 55, 29–38. 10.1042/bse0550029 24070469

[B166] Tu Q. Li Y. Jin J. Jiang X. Ren Y. He Q. (2019). Curcumin alleviates diabetic nephropathy via inhibiting podocyte mesenchymal transdifferentiation and inducing autophagy in rats and MPC5 cells. Pharm. Biol. 57 (1), 778–786. 10.1080/13880209.2019.1688843 31741405 PMC6882478

[B167] Vargas J. N. S. Hamasaki M. Kawabata T. Youle R. J. Yoshimori T. (2023). The mechanisms and roles of selective autophagy in mammals. Nat. Rev. Mol. Cell Biol. 24 (3), 167–185. 10.1038/s41580-022-00542-2 36302887

[B168] Wang B. Yang Q. Sun Y. Y. Xing Y. F. Wang Y. B. Lu X. T. (2014). Resveratrol-enhanced autophagic flux ameliorates myocardial oxidative stress injury in diabetic mice. J. Cell Mol. Med. 18 (8), 1599–1611. 10.1111/jcmm.12312 24889822 PMC4190906

[B169] Wang G. Y. Bi Y. G. Liu X. D. Han J. F. Wei M. Zhang Q. Y. (2017a). Upregulation of connexin 43 and apoptosis-associated protein expression by high glucose in H9c2 cells was improved by resveratrol via the autophagy signaling pathway. Mol. Med. Rep. 16 (3), 3262–3268. 10.3892/mmr.2017.6953 28713934 PMC5547968

[B170] Wang J. Tan J. Luo J. Huang P. Zhou W. Chen L. (2017b). Enhancement of scutellarin oral delivery efficacy by vitamin B12-modified amphiphilic chitosan derivatives to treat type II diabetes induced-retinopathy. J. Nanobiotechnology 15 (1), 18. 10.1186/s12951-017-0251-z 28249594 PMC5333415

[B171] Wang L. Chopp M. Szalad A. Lu X. Zhang Y. Wang X. (2020a). Exosomes derived from Schwann cells ameliorate peripheral neuropathy in type 2 diabetic mice. Diabetes 69 (4), 749–759. 10.2337/db19-0432 31915154 PMC7085247

[B172] Wang L. Klionsky D. J. Shen H. M. (2023). The emerging mechanisms and functions of microautophagy. Nat. Rev. Mol. Cell Biol. 24 (3), 186–203. 10.1038/s41580-022-00529-z 36097284

[B173] Wang L. Sun X. Zhu M. Du J. Xu J. Qin X. (2019a). Epigallocatechin-3-gallate stimulates autophagy and reduces apoptosis levels in retinal Müller cells under high-glucose conditions. Exp. Cell Res. 380 (2), 149–158. 10.1016/j.yexcr.2019.04.014 30998948

[B174] Wang M. Xie M. Yu S. Shang P. Zhang C. Han X. (2021). Lipin1 alleviates autophagy disorder in sciatic nerve and improves diabetic peripheral neuropathy. Mol. Neurobiol. 58 (11), 6049–6061. 10.1007/s12035-021-02540-5 34435332

[B175] Wang Q. Ren J. (2016). mTOR-Independent autophagy inducer trehalose rescues against insulin resistance-induced myocardial contractile anomalies: role of p38 MAPK and Foxo1. Pharmacol. Res. 111, 357–373. 10.1016/j.phrs.2016.06.024 27363949 PMC5026602

[B176] Wang Q. Q. Zhai C. Wahafu A. Zhu Y. T. Liu Y. H. Sun L. Q. (2019b). Salvianolic acid B inhibits the development of diabetic peripheral neuropathy by suppressing autophagy and apoptosis. J. Pharm. Pharmacol. 71 (3), 417–428. 10.1111/jphp.13044 30537209

[B177] Wang S. Zhao Z. Feng X. Cheng Z. Xiong Z. Wang T. (2018a). Melatonin activates Parkin translocation and rescues the impaired mitophagy activity of diabetic cardiomyopathy through Mst1 inhibition. J. Cell Mol. Med. 22 (10), 5132–5144. 10.1111/jcmm.13802 30063115 PMC6156356

[B178] Wang X. Gao L. Lin H. Song J. Wang J. Yin Y. (2018b). Mangiferin prevents diabetic nephropathy progression and protects podocyte function via autophagy in diabetic rat glomeruli. Eur. J. Pharmacol. 824, 170–178. 10.1016/j.ejphar.2018.02.009 29444469

[B179] Wang X. Gao Y. Tian N. Wang T. Shi Y. Xu J. (2019c). Astragaloside IV inhibits glucose-induced epithelial-mesenchymal transition of podocytes through autophagy enhancement via the SIRT-NF-κB p65 axis. Sci. Rep. 9 (1), 323. 10.1038/s41598-018-36911-1 30674969 PMC6344540

[B180] Wang Y. Liu X. Zhu L. Li W. Li Z. Lu X. (2020b). PG545 alleviates diabetic retinopathy by promoting retinal Müller cell autophagy to inhibit the inflammatory response. Biochem. Biophys. Res. Commun. 531 (4), 452–458. 10.1016/j.bbrc.2020.07.134 32800548

[B181] Webb A. E. Brunet A. (2014). FOXO transcription factors: key regulators of cellular quality control. Trends Biochem. Sci. 39 (4), 159–169. 10.1016/j.tibs.2014.02.003 24630600 PMC4021867

[B182] Wei H. Qu H. Wang H. Ji B. Ding Y. Liu D. (2017a). 1,25-Dihydroxyvitamin-D3 prevents the development of diabetic cardiomyopathy in type 1 diabetic rats by enhancing autophagy via inhibiting the β-catenin/TCF4/GSK-3β/mTOR pathway. J. Steroid Biochem. Mol. Biol. 168, 71–90. 10.1016/j.jsbmb.2017.02.007 28216152

[B183] Wei X. Zheng Y. Ai Y. Li B. (2022). Regulatory effects of astragaloside IV on hyperglycemia-induced mitophagy in Schwann cells. Evid. Based Complement. Altern. Med. 2022, 7864308. 10.1155/2022/7864308 PMC8767404 35069769

[B184] Wei Y. Gao J. Qin L. Xu Y. Shi H. Qu L. (2017b). Curcumin suppresses AGEs induced apoptosis in tubular epithelial cells via protective autophagy. Exp. Ther. Med. 14 (6), 6052–6058. 10.3892/etm.2017.5314 29285156 PMC5740722

[B185] Wible D. J. Bratton S. B. (2018). Reciprocity in ROS and autophagic signaling. Curr. Opin. Toxicol. 7, 28–36. 10.1016/j.cotox.2017.10.006 29457143 PMC5810588

[B186] Wong C. H. Iskandar K. B. Yadav S. K. Hirpara J. L. Loh T. Pervaiz S. (2010). Simultaneous induction of non-canonical autophagy and apoptosis in cancer cells by ROS-dependent ERK and JNK activation. PLoS One 5 (4), e9996. 10.1371/journal.pone.0009996 20368806 PMC2848860

[B187] Wu B. Lin J. Luo J. Han D. Fan M. Guo T. (2017). Dihydromyricetin protects against diabetic cardiomyopathy in streptozotocin-induced diabetic mice. Biomed. Res. Int. 2017, 3764370. 10.1155/2017/3764370 28421194 PMC5379084

[B188] Wu F. Li S. Zhang N. Huang W. Li X. Wang M. (2018). Hispidulin alleviates high-glucose-induced podocyte injury by regulating protective autophagy. Biomed. Pharmacother. 104, 307–314. 10.1016/j.biopha.2018.05.017 29775899

[B189] Xiao C. Chen M. Y. Han Y. P. Liu L. J. Yan J. L. Qian L. B. (2023). The protection of luteolin against diabetic cardiomyopathy in rats is related to reversing JNK-suppressed autophagy. Food Funct. 14 (6), 2740–2749. 10.1039/d2fo03871d 36852907

[B190] Xiao T. Guan X. Nie L. Wang S. Sun L. He T. (2014). Rapamycin promotes podocyte autophagy and ameliorates renal injury in diabetic mice. Mol. Cell Biochem. 394 (1-2), 145–154. 10.1007/s11010-014-2090-7 24850187

[B191] Xie M. Morales C. R. Lavandero S. Hill J. A. (2011). Tuning flux: autophagy as a target of heart disease therapy. Curr. Opin. Cardiol. 26 (3), 216–222. 10.1097/HCO.0b013e328345980a 21415729 PMC3607640

[B192] Xu J. Liu L. Q. Xu L. L. Xing Y. Ye S. (2020a). Metformin alleviates renal injury in diabetic rats by inducing Sirt1/FoxO1 autophagic signal axis. Clin. Exp. Pharmacol. Physiol. 47 (4), 599–608. 10.1111/1440-1681.13226 31821581

[B193] Xu K. Liu X. F. Ke Z. Q. Yao Q. Guo S. Liu C. (2018). Resveratrol modulates apoptosis and autophagy induced by high glucose and palmitate in cardiac cells. Cell Physiol. Biochem. 46 (5), 2031–2040. 10.1159/000489442 29723857

[B194] Xu X. Chen B. Huang Q. Wu Y. Liang T. (2020b). The effects of puerarin on autophagy through regulating of the PERK/eIF2α/ATF4 signaling pathway influences renal function in diabetic nephropathy. Diabetes Metab. Syndr. Obes. 13, 2583–2592. 10.2147/DMSO.S256457 32765037 PMC7381766

[B195] Xu X. Kobayashi S. Chen K. Timm D. Volden P. Huang Y. (2013). Diminished autophagy limits cardiac injury in mouse models of type 1 diabetes. J. Biol. Chem. 288 (25), 18077–18092. 10.1074/jbc.M113.474650 23658055 PMC3689952

[B196] Xu X. H. Ding D. F. Yong H. J. Dong C. L. You N. Ye X. L. (2017). Resveratrol transcriptionally regulates miRNA-18a-5p expression ameliorating diabetic nephropathy via increasing autophagy. Eur. Rev. Med. Pharmacol. Sci. 21 (21), 4952–4965. 29164562

[B197] Yang L. Tian L. Zhang Z. Zhou X. Ji X. Liu F. (2020). Cannabinoid receptor 1/miR-30b-5p Axis governs macrophage NLRP3 expression and inflammasome activation in liver inflammatory disease. Mol. Ther. Nucleic Acids 20, 725–738. 10.1016/j.omtn.2020.04.010 32408051 PMC7225604

[B198] Yang S. Xia C. Li S. Du L. Zhang L. Hu Y. (2014). Mitochondrial dysfunction driven by the LRRK2-mediated pathway is associated with loss of Purkinje cells and motor coordination deficits in diabetic rat model. Cell Death Dis. 5 (5), e1217. 10.1038/cddis.2014.184 24810053 PMC4047887

[B199] Yang X. Zhao X. Liu Y. Liu Y. Liu L. An Z. (2023). Ginkgo biloba extract protects against diabetic cardiomyopathy by restoring autophagy via adenosine monophosphate-activated protein kinase/mammalian target of the rapamycin pathway modulation. Phytother. Res. 37 (4), 1377–1390. 10.1002/ptr.7746 36751963

[B200] Yang Z. Tan T. E. Shao Y. Wong T. Y. Li X. (2022). Classification of diabetic retinopathy: past, present and future. Front. Endocrinol. (Lausanne) 13, 1079217. 10.3389/fendo.2022.1079217 36589807 PMC9800497

[B201] Yao Q. Ke Z. Q. Guo S. Yang X. S. Zhang F. X. Liu X. F. (2018). Curcumin protects against diabetic cardiomyopathy by promoting autophagy and alleviating apoptosis. J. Mol. Cell Cardiol. 124, 26–34. 10.1016/j.yjmcc.2018.10.004 30292723

[B202] Yarmohammadi F. Barangi S. Aghaee-Bakhtiari S. H. Hosseinzadeh H. Moosavi Z. Reiter R. J. (2023). Melatonin ameliorates arsenic-induced cardiotoxicity through the regulation of the Sirt1/Nrf2 pathway in rats. Biofactors 49 (3), 620–635. 10.1002/biof.1934 36609811

[B203] Yerra V. G. Kalvala A. K. Kumar A. (2017). Isoliquiritigenin reduces oxidative damage and alleviates mitochondrial impairment by SIRT1 activation in experimental diabetic neuropathy. J. Nutr. Biochem. 47, 41–52. 10.1016/j.jnutbio.2017.05.001 28528294

[B204] Yerra V. G. Kalvala A. K. Sherkhane B. Areti A. Kumar A. (2018). Adenosine monophosphate-activated protein kinase modulation by berberine attenuates mitochondrial deficits and redox imbalance in experimental diabetic neuropathy. Neuropharmacology 131, 256–270. 10.1016/j.neuropharm.2017.12.029 29273519

[B205] Yin Y. Qu H. Yang Q. Fang Z. Gao R. (2021). Astragaloside IV alleviates Schwann cell injury in diabetic peripheral neuropathy by regulating microRNA-155-mediated autophagy. Phytomedicine 92, 153749. 10.1016/j.phymed.2021.153749 34601220

[B206] Yong J. Johnson J. D. Arvan P. Han J. Kaufman R. J. (2021). Therapeutic opportunities for pancreatic β-cell ER stress in diabetes mellitus. Nat. Rev. Endocrinol. 17 (8), 455–467. 10.1038/s41574-021-00510-4 34163039 PMC8765009

[B207] Yu H. Zhen J. Yang Y. Gu J. Wu S. Liu Q. (2016). Ginsenoside Rg1 ameliorates diabetic cardiomyopathy by inhibiting endoplasmic reticulum stress-induced apoptosis in a streptozotocin-induced diabetes rat model. J. Cell Mol. Med. 20 (4), 623–631. 10.1111/jcmm.12739 26869403 PMC5125941

[B208] Yu J. Ke L. Zhou J. Ding C. Yang H. Yan D. (2023). Stachydrine relieved the inflammation and promoted the autophagy in diabetes retinopathy through activating the AMPK/SIRT1 signaling pathway. Diabetes Metab. Syndr. Obes. 16, 2593–2604. 10.2147/DMSO.S420253 37649589 PMC10464895

[B209] Yuan Q. Zhang X. Wei W. Zhao J. Wu Y. Zhao S. (2022). Lycorine improves peripheral nerve function by promoting Schwann cell autophagy via AMPK pathway activation and MMP9 downregulation in diabetic peripheral neuropathy. Pharmacol. Res. 175, 105985. 10.1016/j.phrs.2021.105985 34863821

[B210] Yuan S. Ma T. Zhang Y.-N. Wang N. Baloch Z. Ma K. (2023). Novel drug delivery strategies for antidepressant active ingredients from natural medicinal plants: the state of the art. J. Nanobiotechnology 21 (1), 391. 10.1186/s12951-023-02159-9 37884969 PMC10604811

[B211] Yuan X. Xiao Y. C. Zhang G. P. Hou N. Wu X. Q. Chen W. L. (2016). Chloroquine improves left ventricle diastolic function in streptozotocin-induced diabetic mice. Drug Des. Devel Ther. 10, 2729–2737. 10.2147/DDDT.S111253 PMC5012595 27621594

[B212] Zhan H. Jin J. Liang S. Zhao L. Gong J. He Q. (2019). Tripterygium glycoside protects diabetic kidney disease mouse serum-induced podocyte injury by upregulating autophagy and downregulating β-arrestin-1. Histol. Histopathol. 34 (8), 943–952. 10.14670/HH-18-097 30839094

[B213] Zhan X. Yan C. Chen Y. Wei X. Xiao J. Deng L. (2018). Celastrol antagonizes high glucose-evoked podocyte injury, inflammation and insulin resistance by restoring the HO-1-mediated autophagy pathway. Mol. Immunol. 104, 61–68. 10.1016/j.molimm.2018.10.021 30439604

[B214] Zhang M. Lin J. Wang S. Cheng Z. Hu J. Wang T. (2017a). Melatonin protects against diabetic cardiomyopathy through Mst1/Sirt3 signaling. J. Pineal Res. 63 (2). 10.1111/jpi.12418 28480597

[B215] Zhang M. Wang S. Cheng Z. Xiong Z. Lv J. Yang Z. (2017b). Polydatin ameliorates diabetic cardiomyopathy via Sirt3 activation. Biochem. Biophys. Res. Commun. 493 (3), 1280–1287. 10.1016/j.bbrc.2017.09.151 28965951

[B216] Zhang P. Fang J. Zhang J. Ding S. Gan D. (2020). Curcumin inhibited podocyte cell apoptosis and accelerated cell autophagy in diabetic nephropathy via regulating Beclin1/UVRAG/Bcl2. Diabetes Metab. Syndr. Obes. 13, 641–652. 10.2147/DMSO.S237451 32184643 PMC7060797

[B217] Zhang X. You L. Y. Zhang Z. Y. Jiang D. X. Qiu Y. Ruan Y. P. (2022a). Integrating pharmacological evaluation and computational identification for deciphering the action mechanism of Yunpi-Huoxue-Sanjie formula alleviates diabetic cardiomyopathy. Front. Pharmacol. 13, 957829. 10.3389/fphar.2022.957829 36147338 PMC9487204

[B218] Zhang X. X. Ji Y. L. Zhu L. P. Wang Z. H. Fang C. Q. Jiang C. H. (2022b). Arjunolic acid from Cyclocarya paliurus ameliorates diabetic retinopathy through AMPK/mTOR/HO-1 regulated autophagy pathway. J. Ethnopharmacol. 284, 114772. 10.1016/j.jep.2021.114772 34688801

[B219] Zhang X. X. Jiang C. H. Liu Y. Lou D. X. Huang Y. P. Gao M. (2019). Cyclocarya paliurus triterpenic acids fraction attenuates kidney injury via AMPK-mTOR-regulated autophagy pathway in diabetic rats. Phytomedicine 64, 153060. 10.1016/j.phymed.2019.153060 31401495

[B220] Zhang X. X. Liu Y. Xu S. S. Yang R. Jiang C. H. Zhu L. P. (2022c). Asiatic acid from Cyclocarya paliurus regulates the autophagy-lysosome system via directly inhibiting TGF-β type I receptor and ameliorates diabetic nephropathy fibrosis. Food Funct. 13 (10), 5536–5546. 10.1039/d1fo02445k 35531774

[B221] Zhang Z. Wang S. Zhou S. Yan X. Wang Y. Chen J. (2014). Sulforaphane prevents the development of cardiomyopathy in type 2 diabetic mice probably by reversing oxidative stress-induced inhibition of LKB1/AMPK pathway. J. Mol. Cell Cardiol. 77, 42–52. 10.1016/j.yjmcc.2014.09.022 25268649

[B222] Zhao G. Zhang X. Wang H. Chen Z. (2020). Beta carotene protects H9c2 cardiomyocytes from advanced glycation end product-induced endoplasmic reticulum stress, apoptosis, and autophagy via the PI3K/Akt/mTOR signaling pathway. Ann. Transl. Med. 8 (10), 647. 10.21037/atm-20-3768 32566584 PMC7290636

[B223] Zhao K. Li Y. Qiu Y. Huang R. Lin M. Chen L. (2022). Norkurarinone and isoxanthohumol inhibit high glucose and hypoxia-induced angiogenesis via improving oxidative stress and regulating autophagy in human retinal microvascular endothelial cells. Biochem. Biophys. Res. Commun. 634, 20–29. 10.1016/j.bbrc.2022.09.095 36228541

[B224] Zheng D. Chen L. Li G. Jin L. Wei Q. Liu Z. (2022). Fucoxanthin ameliorated myocardial fibrosis in STZ-induced diabetic rats and cell hypertrophy in HG-induced H9c2 cells by alleviating oxidative stress and restoring mitophagy. Food Funct. 13 (18), 9559–9575. 10.1039/d2fo01761j 35997158

[B225] Zhong Y. Jin R. Luo R. Liu J. Ren L. Zhang Y. (2023). Diosgenin targets CaMKK2 to alleviate type II diabetic nephropathy through improving autophagy, mitophagy and mitochondrial dynamics. Nutrients 15 (16), 3554. 10.3390/nu15163554 37630743 PMC10459415

[B226] Zhong Y. Liu J. Sun D. Guo T. Yao Y. Xia X. (2022a). Dioscin relieves diabetic nephropathy via suppressing oxidative stress and apoptosis, and improving mitochondrial quality and quantity control. Food Funct. 13 (6), 3660–3673. 10.1039/d1fo02733f 35262539

[B227] Zhong Y. Luo R. Liu Q. Zhu J. Lei M. Liang X. (2022b). Jujuboside A ameliorates high fat diet and streptozotocin induced diabetic nephropathy via suppressing oxidative stress, apoptosis, and enhancing autophagy. Food Chem. Toxicol. 159, 112697. 10.1016/j.fct.2021.112697 34826549

[B228] Zhou G. Hu R. K. Xia G. C. Yan S. H. Ren Q. L. Zhao J. (2019a). Tyrosine nitrations impaired intracellular trafficking of FSHR to the cell surface and FSH-induced Akt-FoxO3a signaling in human granulosa cells. Aging (Albany NY) 11 (10), 3094–3116. 10.18632/aging.101964 31097679 PMC6555443

[B229] Zhou H. Chen Y. Huang S. W. Hu P. F. Tang L. J. (2018). Regulation of autophagy by tea polyphenols in diabetic cardiomyopathy. J. Zhejiang Univ. Sci. B 19 (5), 333–341. 10.1631/jzus.B1700415 29732743 PMC5962509

[B230] Zhou H. Ding S. Sun C. Fu J. Yang D. Wang X. (2021). Lycium barbarum extracts extend lifespan and alleviate proteotoxicity in *Caenorhabditis elegans* . Front. Nutr. 8, 815947. 10.3389/fnut.2021.815947 35096951 PMC8790518

[B231] Zhou P. Xie W. Meng X. Zhai Y. Dong X. Zhang X. (2019b). Notoginsenoside R1 ameliorates diabetic retinopathy through PINK1-dependent activation of mitophagy. Cells 8 (3), 213. 10.3390/cells8030213 30832367 PMC6468581

[B232] Zhu Y. Qian X. Li J. Lin X. Luo J. Huang J. (2019). Astragaloside-IV protects H9C2(2-1) cardiomyocytes from high glucose-induced injury via miR-34a-mediated autophagy pathway. Artif. Cells Nanomed Biotechnol. 47 (1), 4172–4181. 10.1080/21691401.2019.1687492 31713440

[B233] Zhu Y. Xia X. He Q. Xiao Q. A. Wang D. Huang M. (2023). Diabetes-associated neutrophil NETosis: pathogenesis and interventional target of diabetic complications. Front. Endocrinol. (Lausanne) 14, 1202463. 10.3389/fendo.2023.1202463 37600700 PMC10435749

